# Heart-Type Fatty Acid Binding Protein Binds Long-Chain Acylcarnitines and Protects against Lipotoxicity

**DOI:** 10.3390/ijms24065528

**Published:** 2023-03-14

**Authors:** Diana Zelencova-Gopejenko, Melita Videja, Aiga Grandane, Linda Pudnika-Okinčica, Anda Sipola, Karlis Vilks, Maija Dambrova, Kristaps Jaudzems, Edgars Liepinsh

**Affiliations:** 1 Department of Physical Organic Chemistry, Latvian Institute of Organic Synthesis, Aizkraukles 21, LV-1006 Riga, Latvia; 2 Faculty of Materials Science and Applied Chemistry, Riga Technical University, Paula Valdena 3, LV-1048 Riga, Latvia; 3 Laboratory of Pharmaceutical Pharmacology, Latvian Institute of Organic Synthesis, Aizkraukles 21, LV-1006 Riga, Latvia; 4 Faculty of Pharmacy, Rīga Stradinš University, Dzirciema 16, LV-1007 Riga, Latvia; 5 Organic Synthesis Group, Latvian Institute of Organic Synthesis, Aizkraukles 21, LV-1006 Riga, Latvia; 6 Laboratory of Membrane Active Compounds and β-Diketones, Latvian Institute of Organic Synthesis, Aizkraukles 21, LV-1006 Riga, Latvia

**Keywords:** long-chain acylcarnitines, palmitoylcarnitine, fatty acids, palmitate, heart-type fatty acid-binding protein, FABP3, isothermal titration calorimetry, nuclear magnetic resonance

## Abstract

Heart-type fatty-acid binding protein (FABP3) is an essential cytosolic lipid transport protein found in cardiomyocytes. FABP3 binds fatty acids (FAs) reversibly and with high affinity. Acylcarnitines (ACs) are an esterified form of FAs that play an important role in cellular energy metabolism. However, an increased concentration of ACs can exert detrimental effects on cardiac mitochondria and lead to severe cardiac damage. In the present study, we evaluated the ability of FABP3 to bind long-chain ACs (LCACs) and protect cells from their harmful effects. We characterized the novel binding mechanism between FABP3 and LCACs by a cytotoxicity assay, nuclear magnetic resonance, and isothermal titration calorimetry. Our data demonstrate that FABP3 is capable of binding both FAs and LCACs as well as decreasing the cytotoxicity of LCACs. Our findings reveal that LCACs and FAs compete for the binding site of FABP3. Thus, the protective mechanism of FABP3 is found to be concentration dependent.

## 1. Introduction

Fatty acids (FAs) are essential components of many biological processes, functioning as the mitochondrial energy source and intra- and extracellular signaling molecules [1,2]. They also play a crucial role in the pathogenesis of various diseases, such as cancer [3], inflammation [4], and metabolic diseases [5,6]. Homeostasis of FAs in living cells depends on the balance between their availability and elimination; as a result, the transport mechanism becomes especially important. The relatively weak solubility of FAs and the cytotoxicity of their free forms are resolved by the intracellular lipid transporters fatty acid-binding proteins (FABPs) [1,7,8]. Thus, FABPs regulate intracellular functions, such as synthesis of membrane phospholipids, lipid metabolism, and mitochondrial β-oxidation [1,9].

**Figure 1:**
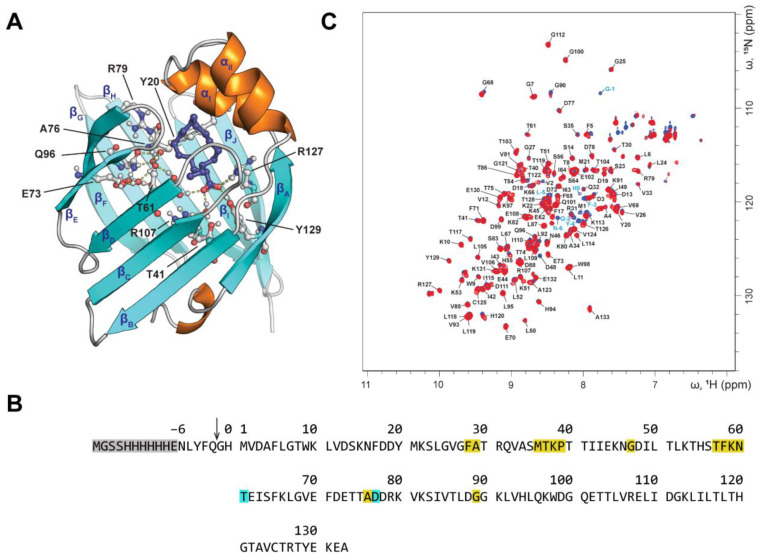
(**A**) Structure of heart-type fatty acid-binding protein (FABP3, PDB ID 3WVM [15]) in complex with palmitate (C16:0). Protein structure is represented as ribbons and colored according to the secondary structure elements as follows: α-helixes shown in orange, β-sheets in cyan, and loops in gray. C16:0 is shown as dark blue sticks, while residues involved in ligand and water cluster binding are shown as light gray balls and sticks. Water molecules (red spheres) and hydrogen bonds (yellow dashed lines) are shown. (**B**) Human FABP3 amino acid sequence. Residues colored in yellow were not assigned from the NMR spectra either for the apo-form or for the holo-form, while residues in cyan were not assigned only for complexes of FABP3 with acylcarnitines (ACs). Crosspeaks for residues colored in gray were not observed in any 2D ^1^H-^15^N HSQC spectra. The black arrow points to the position of the TEV cleavage site. (**C**) Assigned 2D ^1^H-^15^N HSQC spectra of human apo-FABP3 in 20 mM K_2_HPO_4_/KH_2_PO_4_, 50 mM KCl buffer pH 7.6 (KPi). His-tagged FABP3 (noncleaved) is shown in blue, and cleaved FABP3 is shown in red. Backbone amide resonances are denoted as one letter symbol and residue number according to the sequence in Figure 1B. Labels for the residues from the His-tag are shown in light blue. Side chain amide resonances were not assigned. Residues are numbered according to UniProt ID P05413.

FABPs are small 14–15 kDa globular proteins that belong to the family of lipid-binding proteins. Currently, at least ten tissue-specific isoforms of FABPs are known that share 20–70% amino acid sequence homology and highly similar tertiary structures [10,11]. The structure of FABPs consists of two α-helixes and ten β-strands, which form a β-barrel with the binding site inside (Figure 1A). Heart-type FABP (hereafter FABP3) is among the ubiquitous FABP isoforms. It can be found in various tissues aside from the heart, including kidney, skeletal and smooth muscle, brain, aorta, placenta, testis, ovary, lung, mammary epithelial cells, and stomach tissues [1,8,11,12]. On the other hand, FABP3 is the only representative of the FABP family found in the myocardium and red skeletal muscle [13]. Experiments with FABP3 knock-out (KO) mice have shown that by turning off the *FABP3* gene, uptake, and oxidation of long-chain FAs (LCFAs) were reduced significantly by 45–65%, while the oxidation of glucose increased by 80% in cardiomyocytes. The expression levels of the other types of FABPs did not change; thus, no compensation mechanism occurs. Moreover, FABP3 KO mice showed severe exercise intolerance and localized cardiac hypertrophy during aging [13,14]. These data highlight the importance of FABP3 in maintaining LCFA metabolism in the heart.

Under normal physiological conditions for the fed–fasted cycle, plasma concentrations as well as the tissue contents of FAs and their metabolic intermediates acylcarnitines (ACs) vary significantly, reaching several fold higher levels in a fasted state than in a fed state [16,17]. The long-chain AC (LCAC) concentration in the fasted state reaches a level that is potentially toxic to cells in the heart [18,19,20,21,22]. However, the detrimental effects ascribed to LCACs at 10–20 μM concentrations are not observed in the fasted state. Therefore, it is hypothesized that cells are protected from LCACs because they are bound to FABPs [21,23]. It was experimentally demonstrated that the cytosolic fraction from the hearts of fed rats protects mitochondria against LCAC-induced damage, confirming that the cell cytosol contains proteins that could be responsible for the handling of LCACs [21]. However, the evidence was indirect and detailed studies are still necessary for the determination of AC and FABP interactions.

Within the framework of this research, we focused on mechanistic studies of LCACs, which are important metabolic intermediates in the regulation of cellular energy metabolism [24,25,26]. It is well known that acyl-coenzyme A (acyl-CoA) is bound to acyl-CoA-binding protein (ACBP) with high affinity. However, the binding or interaction partner for LCACs remains unknown. We proposed that FABP3 binds both FAs and ACs and protects cells from lipotoxicity. We used cytotoxicity assays, nuclear magnetic resonance (NMR), and isothermal titration calorimetry (ITC) methods to characterize FA and AC binding to FABP3 and to investigate its protective effect.

## 2. Results

### 2.1. LCAC Cytotoxicity Assay

**Figure 2:**
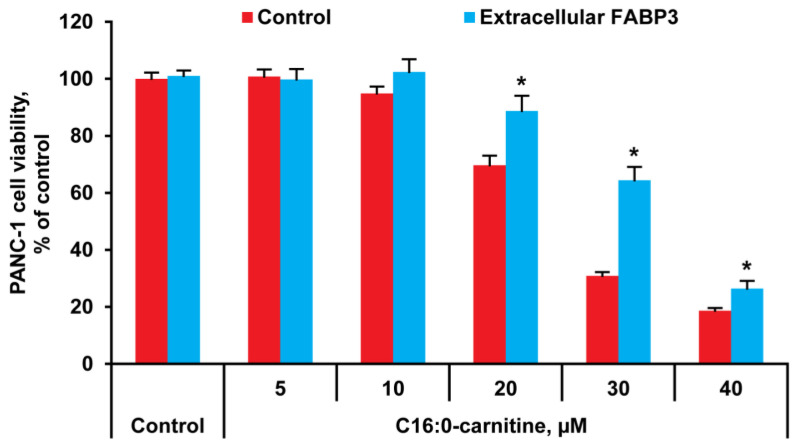
Toxicity of C16:0-carnitine in PANC-1 cells after 4 h of incubation in the presence or in the absence of 60 μM heart-type fatty acid-binding protein (FABP3) in the cell media. Data are shown as the mean ± SEM of 3 independent experiments in at least 6 technical replicates. * indicates a significant difference compared to the cells not subjected to FABP3 treatment at the respective C16:0-carnitine concentration.

To evaluate the protective potential of FABP3 on LCAC-induced toxicity, a cell viability assay was performed in PANC-1 cells. Figure 2 shows that C16:0-carnitine is toxic to PANC-1 cells and reduces cell viability in a concentration-dependent manner with an LC_50_ of approximately 25 μM. However, the addition of FABP3 to the cell media preserved cell viability and we observed an increase in cell viability by 20% on average. Nevertheless, the cytoprotective properties were less pronounced at the highest tested concentration of C16:0-carnitine.

**Figure 3:**
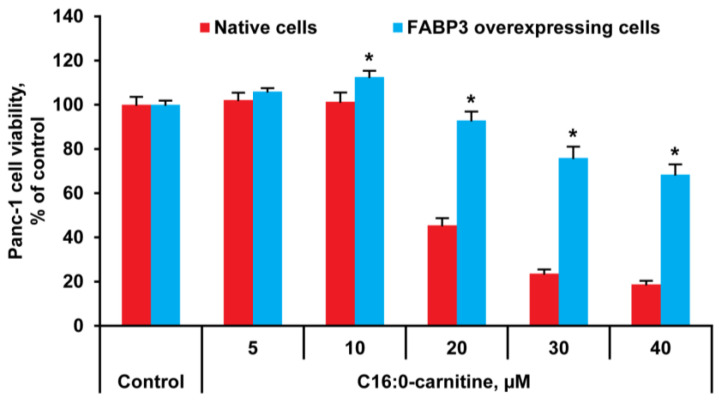
Toxicity of C16:0-carnitine in native and heart-type fatty acid-binding protein (FABP3)-overexpressing PANC-1 cells after 4 h of incubation. Data are shown as the mean ± SEM of 3 independent experiments in at least 6 technical replicates. * indicates a significant difference compared to the native cells at the respective concentration of C16:0-carnitine.

The cytotoxic effects of C16:0-carnitine were effectively diminished with FABP3 overexpression in PANC-1 cells. Figure 3 indicates that even at 40 μM C16:0-carnitine concentration, cells transfected with the FABP3 plasmid retained nearly 70% viability compared to that of native cells, in which the cell viability was below 20%.

### 2.2. FA and AC Binding Studies by NMR Spectroscopy

His-tagged FABP3 consisted of the extra eighteen *N*-terminal residues (Figure 1B, numbered backward from 0 to −17), seven of which (L-6–H0) were assigned. To test whether the His-tag interacts with the protein structure, we acquired two 2D ^1^H-^15^N HSQC spectra for noncleaved and cleaved apo-FABP3. The superposition of the spectra (Figure 1C) shows that the crosspeaks of the residues from L-6 to H0 disappeared and the crosspeaks for three residues in the *N*-terminal part (M1, V2, and F5) slightly shifted after the His-tag was successfully removed. All other chemical shifts remained unchanged. Subsequent binding studies also showed that the His-tag did not impact the conformation of the protein–ligand complex.

The 2D ^1^H-^15^N HSQC spectra for the delipidated and refolded apo-FABP3 with its saturated holo-form obtained from *E. coli* were compared and significant chemical shift perturbations (CSPs) for various crosspeaks were clearly observed (Figure S1A). The addition of C16:0 to apo-FABP3 also caused a large rearrangement of the crosspeaks (Figure S1B), resulting in a spectrum highly similar to that obtained for saturated FABP3. Thus, the functionality of FABP3 was not affected by the refolding process, and the same protein structure was obtained after the protein was resaturated with LCFA. A total of 120 and 117 residues were assigned from 133 backbone amides (excluding His-tag) for apo-FABP3/FABP3-FAs and FABP3-ACs, respectively. Unassigned residues (F28, A29, V33, M36, T37, K38, P39, G47, T57, F58, K59, N60, T61, A76, D77, and G89) correspond to the flexible side chains of the ligand entry portal between α_II_–β_B_, β_C_–β_D_, and β_E_–β_F_ stands (Figure 1A) that underwent fast NH exchange at pH 7.6. The backbone assignment for FABP3-FAs (Figure 1C) was in good agreement with previously published data [27,28].

**Figure 4:**
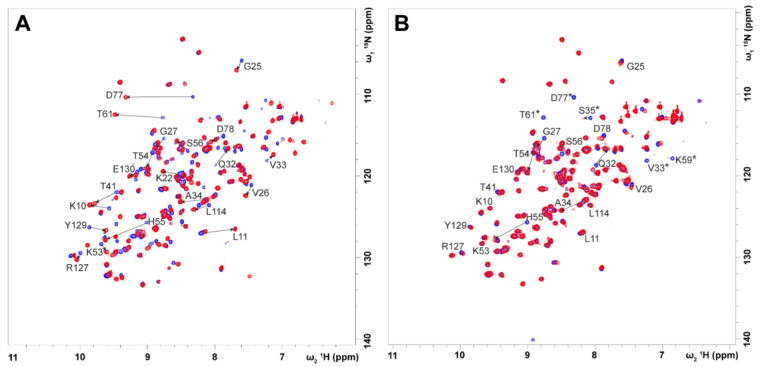
Superposition of the 2D ^1^H-^15^N HSQC spectra for (**A**) heart-type fatty acid-binding protein (FABP3)-C18:1(n-9)*t* and (**B**) FABP3-C18:1(n-9)*t*-carnitine complex in 20 mM K_2_HPO_4_/KH_2_PO_4_, 50 mM KCl buffer pH 7.6 (KPi). The spectrum of apo-FABP3—in blue, spectrum of the FABP3-ligand complex—in red. Residues with chemical shift perturbations (CSPs) larger than the mean plus one standard deviation are assigned and black arrows show the shift of the corresponding crosspeak. * marks the crosspeaks that have disappeared upon binding of the acylcarnitines (ACs).

The overlays of the 2D ^1^H-^15^N HSQC spectra for apo-FABP3 and 18 protein–lipid complexes (holo-FABP3) were analyzed (Figure 4 and Figures S2–S19). All analyzed FAs (Figures S2–S10), C16:0-CoA (Figure S11), and several LCACs [C14:0-, C18:1(n-9)*c*/*t*-, and C20:5(n-3)*c*-carnitines] (Figures S12–S15) produced strong CSPs indicative of binding to FABP3. Saturated medium-chain ACs, such as C8:0- and C12:0-carnitines, did not bind to the protein under experimental conditions (Figures S16 and S17). Saturated LCAC, C16:0-carnitine, that was used in the cell assay did not show any significant CSPs, although the signals of G27, V33, H55, K59, T61, and D77 were significantly reduced in intensity (Figure S18). This result may indicate that the C16:0-carnitine micelles bind to the ligand entry portal. On the other hand, *l*-carnitine (hereafter carnitine) was found to neither bind nor interact with FABP3 (Figure S19).

**Figure 5:**
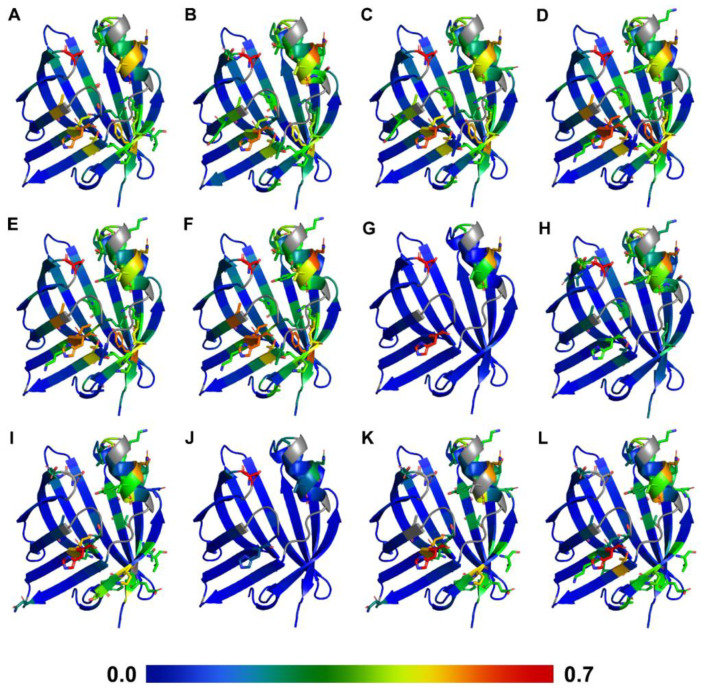
Mapping of the chemical shift perturbations (CSPs) caused by binding of (**A**) C8:0, (**B**) C12:0, (**C**) C14:0, (**D**) C16:0, (**E**) C18:1(n-9)*c*, (**F**) C20:5(n-3)*c*, (**G**) C8:0-carnitine, (**H**) C12:0-carnitine, (**I**) C14:0-carnitine, (**J**) C16:0-carnitine, (**K**) C18:1(n-9)*c*-carnitine, or (**L**) C20:5(n-3)*c*-carnitine onto the heart-type fatty acid-binding protein (FABP3) structure. The CSPs are color-coded, in which red indicates larger shifts, while blue indicates no changes in the averaged *δ_H_* and *δ_N_* NMR chemical shifts. Unassigned or disappeared residues are colored in gray. FABP3 structure is taken from PDB ID 3WVM.

The assigned chemical shifts of the backbone amides were used for the CSP calculation, Δ*δ*, by Equation (1) to map the ligand binding site. The resulting histograms of the Δ*δ* values produced by the FAs and corresponding ACs are summarized in Figures S20 and S21, respectively. Figure 4 presents an example of the rearrangement of the crosspeaks with residues undergoing the most significant CSPs identified. The CSPs obtained for the 12 compounds, FAs, C8:0, C12:0, C14:0, C16:0, C18:1(n-9)*c*, C20:5(n-3)*c*, and ACs of the corresponding length, are mapped on the FABP3 structure in Figure 5, identifying the ligand binding site for each compound. In the case of all FAs, CSPs larger than the mean plus one standard deviation were observed for residues K10, L11, V26, G27, Q32, V33, T41, T54, H55, T61, D77, and Y129 (Figure 4A and Figure S20). S56 showed CSPs + mean only for the FAs with C18 and C20 atoms, while A34 showed CSPs for all FAs except for C8:0 and C20:5(n-3)*c*. Several residues Y20, T30, L114, L116, and R127 located within 4.5 Å from the ligand [29] also showed significant CSPs. Additionally, we observed no difference between the binding of the *cis* and *trans* isomers of the monounsaturated FAs, C18:1(n-9) (Figures S8 and S9). These data are in good agreement with the crystallographic studies [29].

Binding of the ACs caused much smaller changes in the crosspeak positions of FABP3 (Figure S21) in comparison to the matching FAs. In the case of medium-chain ACs (C8:0- and C12:0-carnitines), changes in the chemical shifts were insignificant, while LCACs such as C14:0-, C18:1(n-9)*c*/*t*-, and C20:5(n-3)*c*-carnitines caused noticeable changes in the spectra pointing to the ligand binding event (Figure 4B). Crosspeaks for five residues, V33, S35, K59, T61, and D77, disappeared from the HSQC spectra. The remaining residues with significant CSPs upon LCAC binding were K10, G27, Q32, A34, T54, and H55 (Figure S21C,E–G). Moderate changes in the chemical shifts were also observed for T128 and E130. However, R127 and Y129, which participate in the formation of hydrogen bonds with the carboxyl group of FAs, did not show any CSPs upon binding of LCACs. The largest CSPs were observed for the residues Q32, A34, H55, and D77 belonging to the α_II_ helix or flexible chain regions that are located on top of the ligand entry portal (Figure 1A). A comparison of the data for the *cis* and *trans* isomers of the monounsaturated LCACs also showed no discrepancies in the CSPs, similar to C18:1(n-9)*c*/*t* LCFAs. Likewise, C16:0-CoA was found to bind to FABP3 (Figure S11). However, the CSP pattern caused by its binding (Figure S21H) was more similar to that of LCFAs than that of LCACs. No residues disappeared, while the crosspeaks of the same 12 residues were shifted. Thus, our findings indicate two different binding mechanisms for LCFAs and LCACs. To better characterize the differences in the lipid-binding modes, ITC experiments as well as Δ*C_p_* determination and competitive binding assays were performed.

### 2.3. Thermodynamics of LCFA Binding to FABP3

It is well known that LCFAs are highly insoluble in water. The recent methodology suggests that FAs are solubilized by the preparation of the mixed liposomes with DMPC [15]. Despite good solubilization and cytoplasm-mimicking conditions, this approach cannot be used to determine the precise binding thermodynamics of FAs to FABP3 due to the prevalent heat effects from liposome rearrangement. Moreover, this approach was not suitable for the efficient solubilization of LCACs. Therefore, we developed an ITC methodology to evaluate the binding thermodynamics of LCFAs in aqueous buffers with the solubility-enhancing additive Triton™ X-100 (TX) at 0.1–0.5% (*v*/*v*) concentrations. TX was potent to efficiently solubilize LCFAs, such as C16:0 and C18:0, up to 500 μM concentration and increases their critical micelle concentrations (CMC) [30]. To ensure that TX neither interacts with FABP3 nor affects ligand binding, additional NMR experiments were performed. As shown in the 2D ^1^H-^15^N HSQC spectra (Figure S22A), TX did not interact with the FABP3 core structure. Minimal changes were observed for residues G27, T30, Q32, V33, H55, T61, D77, and D78, which belong to the highly flexible ligand entry portal (Figure 1A, α_II_-helix and loops between β_C_–β_D_ and β_E_–β_F_ strands). Nevertheless, TX did not affect the binding of FAs (Figure S22B).

Several FAs, such as C8:0, C10:0, and C12:0, were soluble enough to perform ITC titration experiments without any buffer supplements. These FAs were used as controls to evaluate the solubilization efficiency of the TX additive and its impact on the thermodynamic parameters in the ITC assay (Table 1, Figures S23–S28). These experiments revealed that the addition of the detergent changes enthalpic, Δ*H*, and entropic, −*T*Δ*S*, contributions to the binding affinity. As shown in Table 1, TX caused a decrease in Δ*H* by 2.1–5.9 kcal∙mol^−1^ and an increase in −*T*Δ*S* of a similar magnitude (i.e., enthalpy-entropy compensation mechanism [31,32]). Nevertheless, the binding mechanism remains enthalpy-driven in both cases, while entropy was found to be either close to zero or binding-unfavorable (positive).

The remaining LCFAs [C14:0, C16:0, C18:0, C18:1(n-9)*c*/*t*, and C20:5(n-3)*c*] were titrated in the presence of 0.1% (*v*/*v*) TX additive. The results are summarized in Table 1 and Figures S29–S34. Here, the same enthalpy-driven binding mechanism was observed. However, in comparison to the shorter FAs, both enthalpic and entropic components decreased (by absolute values). Thus, we can assume that these ligands caused binding-favorable desolvation and conformational changes in the binding site of FABP3 triggered by ligand binding via a U-shape conformation. C8:0 was found to be the weakest binder, while C16:0 was the strongest binder. Increasing the chain length further to C18:0 reduced *K_D_* by five fold. No discrepancy was found between the binding of the *cis* and *trans* monounsaturated LCFAs [C18:1(n-9)*c*/*t*]. The polyunsaturated C20:5(n-3)*c* exhibited a *K_D_* similar to the much shorter C8:0. A large negative Δ*H* was compensated by the binding-unfavorable −*T*Δ*S* of a similar magnitude. Most likely, such contribution of the thermodynamic parameters is related to the more rigid structure of C20:5(n-3)*c* that causes large conformational changes in the binding site of FABP3.

### 2.4. Thermodynamics of LCAC Binding to FABP3

Despite the successful solubilization of LCFAs, the addition of the detergent completely blocked the binding of C16:0-CoA and ACs. Thus, thermodynamic data for these compounds were determined in 20 mM K_2_HPO_4_/KH_2_PO_4_, 50 mM KCl buffer pH 7.6 (hereafter KPi) without any supplements. The results are summarized in Table 1 and Figures S35–S39. Due to solubility and micellization issues, binding thermodynamics were not determined for saturated LCACs, such as C16:0-carnitine (Figure S40), while well-soluble medium-chain ACs (C8:0- and C12:0-carnitines) showed no binding to FABP3 under experimental conditions (Figures S41 and S42), similar to the previously obtained NMR data (Figures S16 and S17). The determined *K_D_* values for LCACs were 1–2 orders of magnitude higher than those for LCFAs, indicating less potent binding. In addition, FABP3 bound C16:0-CoA but with an affinity that was significantly lower than that toward ACBP. LCACs as well as C16:0-CoA showed similar binding profiles with both binding-favorable enthalpy and entropy. However, Δ*H* was approximately two fold smaller (by absolute value) than that of the corresponding LCFAs, pointing to the formation of the suboptimal contact network within the protein–ligand complex.

**Figure 6:**
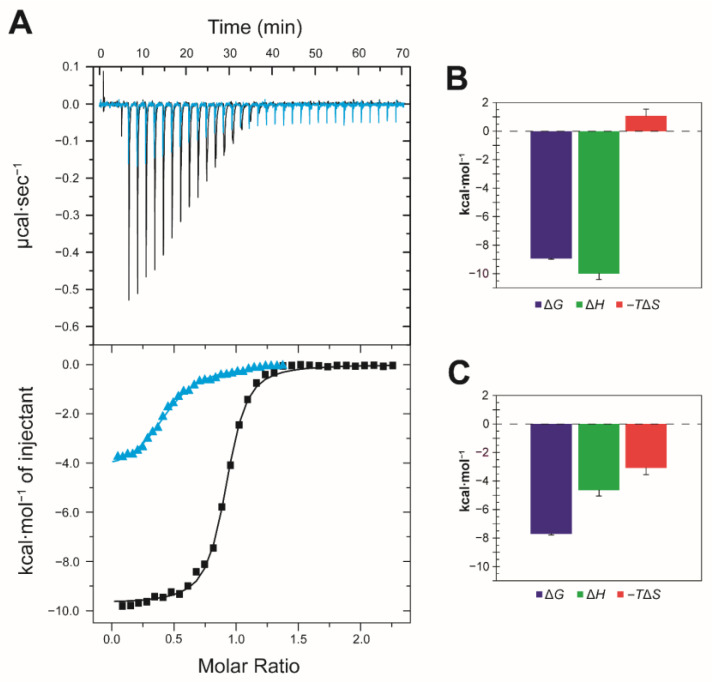
(**A**) Superposition of the ITC titration curves of the heart-type fatty acid-binding protein (FABP3) interaction with C18:1(n-9)*c* in black and C18:1(n-9)*c*-carnitine in cyan. Both experiments were performed in 20 mM K_2_HPO_4_/KH_2_PO_4_, 50 mM KCl buffer pH 7.6 (KPi) at 25 °C. Graphical representation of the thermodynamic binding parameters of the FABP3 interaction with (**B**) C18:1(n-9)*c* and (**C**) C18:1(n-9)*c*-carnitine.

Comparison of the thermodynamic data of LCFAs and monounsaturated LCACs binding to FABP3 revealed a shift in the stoichiometry, *N*, from 1.0 to 0.5, as shown in Figure 6. This indicates that C18:1(n-9)*c*-carnitine, C18:1(n-9)*t*-carnitine, and possibly C14:0-carnitine facilitate the binding of two molecules of FABP3 per ligand molecule. This stoichiometry was maintained even with varying concentrations of both protein and ligand as well as their ratio. On the other hand, C20:5(n-3)*c*-carnitine and C16:0-CoA still bind with a stoichiometry of one to one. To characterize the binding mechanism of monounsaturated LCACs, changes in the binding heat capacity, Δ*C_p_*, and competitive binding experiments were performed.

### 2.5. Determination of Changes in the Heat Capacity for LCFA and LCAC Binding to FABP3

**Figure 7:**
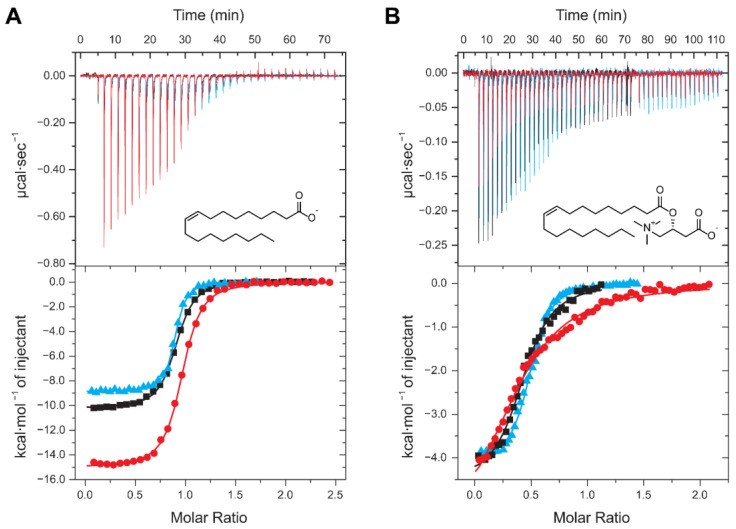
Superposition of the ITC thermograms for heart-type fatty acid-binding protein (FABP3) titrated with (**A**) C18:1(n-9)*c* and (**B**) C18:1(n-9)*c*-carnitine at three different temperatures: 16 (cyan), 25 (black), and 37 °C (red).

Heat capacity changes, Δ*C_p_*, are important indicators of the structural changes during ligand-to-protein binding in various environmental conditions and may reflect changes in the static and dynamic properties of the protein [33,34]. Moreover, it may represent interactions with extra supplements, such as salts and detergents, and larger processes, such as changes in protein folding, solvent-accessible surface area (SASA), or protonation states within the active site [35,36]. Therefore, the changes in the heat capacity were determined for the binding of four FAs [C12:0, C18:1(n-9)*c*, C18:1(n-9)*t*, and C20:5(n-3)*c*] and three corresponding LCACs [C18:1(n-9)*c*-, C18:1(n-9)*t*-, and C20:5(n-3)*c*-carnitines] to FABP3. C12:0 binding was determined in two KPi buffers, both without and with the TX additive, to evaluate the detergent effect on the protein structure as well. Thermodynamic parameters for the remaining LCFAs were determined in the KPi + TX buffer, while LCACs were tested in the KPi buffer without any additive. Figure 7 represents an example of the overlays of the ITC experiments for C18:1(n-9)*c* and the corresponding LCAC performed at the following temperatures: 16, 25, and 37 °C.

**Figure 8:**
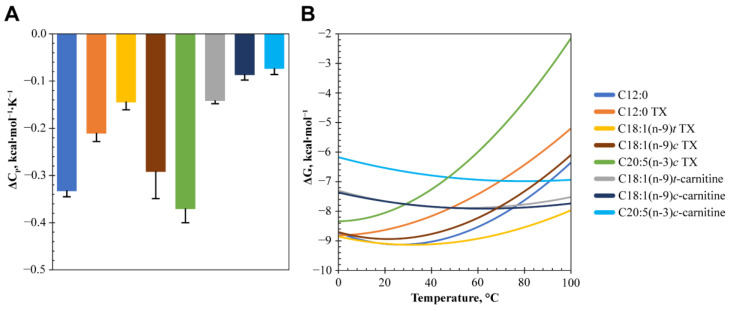
Heat capacity change, Δ*C_p_*, and temperature dependence of the binding free energy, Δ*G*. (**A**) Δ*C_p_* values for four fatty acids (FAs) and corresponding acylcarnitines (ACs) binding to heart-type fatty acid-binding protein (FABP3). (**B**) Δ*G* of FA or AC binding to FABP3 within the temperature range from 0 to 100 °C.

The resulting temperature dependencies of the binding enthalpies, Δ*H*, are represented in Figure S43. Through linear regression analysis, we determined Δ*C_p_* values with coefficients of determination, *R^2^*, in the range from 0.964 to 0.998, and relatively small experimental errors were obtained (Figure 8A and Table 2). Based on the reference thermodynamic data (determined at 25 °C) and Δ*C_p_* values and solving Equations (S1)–(S3), the dependence of the binding free energy, Δ*G*, on the temperature was evaluated (Figure 8B). Δ*G* remains stable at common experimental temperatures from 25 to 37 °C (deviations < 0.5 kcal∙mol^−1^). All three unsaturated LCACs retain small variability in Δ*G* (< 0.8 kcal∙mol^−1^) within the whole 100 °C interval, while LCFAs display ΔΔ*G* in the range from 1.2 to 6.2 kcal∙mol^−1^.

ITC experiments on C12:0 binding to FABP3 in KPi buffer both without and with 0.1% (*v*/*v*) TX additive produced Δ*C_p_* values of −0.33 and −0.22 kcal∙mol^−1^∙K^−1^, respectively. The determined values differ by 0.11 kcal∙mol^−1^∙K^−1^ and both are similar to the Δ*C_p_* of the phosphate buffer (pK_a_ 7.198), which equals −0.23 kcal∙mol^−1^∙K^−1^ [38]. A slight increase in Δ*C_p_* for the buffer with detergent may indicate that the ligand became less hydrophobic under these experimental conditions. Furthermore, it might point to the slight increase in the SASA and the hydration of the binding site. The remaining three unsaturated LCFAs [C18:1(n-9)*c*/*t* and C20:5(n-3)*c*] also have similar Δ*C_p_* values from −0.37 to −0.16 kcal∙mol^−1^∙K^−1^. The corresponding unsaturated LCACs have smaller Δ*C_p_* values ranging from −0.10 to −0.08 kcal∙mol^−1^∙K^−1^. This clustering is in good agreement with the hydrophobicity of the molecules, i.e., LCFAs that are more hydrophobic than LCACs also exhibit more negative Δ*C_p_* values. However, no correlation between the Δ*C_p_* and binding affinity was observed. The Δ*H* of all tested FAs varied more significantly by 5.6 ± 1.9 kcal∙mol^−1^ on average with the change in the temperature, while the Δ*H* of LCACs showed smaller changes of approximately 2.1 ± 0.8 kcal∙mol^−1^. Similar to the NMR results, these data also support two different binding mechanisms for LCFAs and LCACs.

### 2.6. Competitive Binding Experiments for ACs

**Figure 9:**
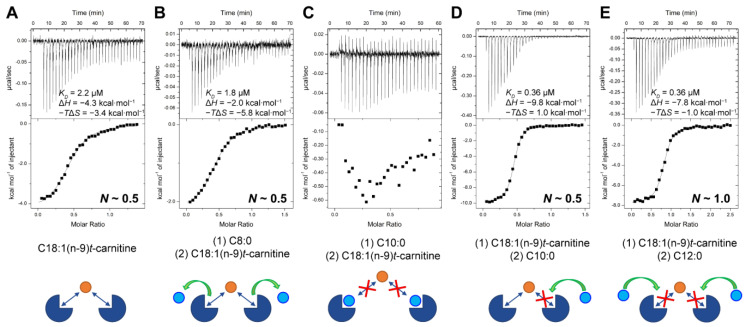
Top. Competitive binding studies of heart-type fatty acid-binding protein (FABP3)-C18:1(n-9)*t*-carnitine and three fatty acids (FAs) of different chain lengths. (**A**) Reference titration of C18:1(n-9)*t*-carnitine to apo-FABP3. (**B**) FABP3-C8:0 complex titrated with C18:1(n-9)*t*-carnitine. (**C**) FABP3-C10:0 complex titrated with C18:1(n-9)*t*-carnitine. (**D**) FABP3-C18:1(n-9)*t*-carnitine complex titrated with C10:0. (**E**) FABP3-C18:1(n-9)*t*-carnitine complex titrated with C12:0. All experiments were performed in 20 mM K_2_HPO_4_/KH_2_PO_4_ and 50 mM KCl buffer pH 7.6 (KPi) at 25 °C. Bottom. Schematic representation of ligand binding and competition. Protein is represented as the dark blue sector, acylcarnitines (ACs) as orange, and FAs as cyan circles. Green arrows indicate that the competition event between ligands was successful.

To characterize the novel binding mechanism of the monounsaturated LCACs, competitive binding experiments with FAs of variable chain lengths were performed. As C18:1(n-9)*c*-carnitine and C18:1(n-9)*t*-carnitine showed identical binding data, subsequent experiments were performed with C18:1(n-9)*t*-carnitine only. The results are summarized in Figure 9 and Figures S44–S48.

First, competition experiments between the medium-chain FAs and C18:1(n-9)*t*-carnitine were performed. FABP3 was saturated with C8:0 and then titrated with C18:1(n-9)*t*-carnitine. The resulting thermogram is shown in Figure 9B. Here, C18:1(n-9)*t*-carnitine substituted C8:0 in the binding site of FABP3, providing the stoichiometry, *N*, 0.5. The binding affinity remained highly similar to that of the reference titration (Figure 9A and Figure S39). However, slight deviations within ~2 kcal∙mol^−1^ in the thermodynamic parameters were observed.

Next, FABP3 was saturated with C10:0 and then titrated with C18:1(n-9)*t*-carnitine. As shown in Figure 9C, C18:1(n-9)*t*-carnitine could not replace C10:0. Thus, no binding heat effects were detected. The opposite experiment, in which the protein was first saturated with C18:1(n-9)*t*-carnitine and then titrated with C10:0, produced intriguing results (Figure 9D). C10:0 was potent enough to replace C18:1(n-9)*t*-carnitine. However, the determined stoichiometry dropped to 0.5 and was not affected by the changes in the protein–ligand concentrations or their ratio. The binding affinity of C10:0 to the FABP3-C18:1(n-9)*t*-carnitine complex was similar to the affinity of C10:0 to apo-FABP3 (Table 1, Figure S23).

The experiment in which the FABP3-C18:1(n-9)*t*-carnitine complex was titrated with C12:0 resulted in an S-shaped curve with the stoichiometry, *N*, 1.0 (Figure 9E). The determined thermodynamic parameters matched the data from the titration of apo-FABP3 (Table 1, Figure S27) within 0.5–0.8 kcal∙mol^−1^ deviation. Thus, C12:0 is a stronger binder than C10:0 and C18:1(n-9)*t*-carnitine and easily replaces LCACs in both FABP3 binding sites.

The last titration in this series was performed with C16:0. Previous binding experiments with ACs in the presence of 0.1% (*v*/*v*) TX were unsuccessful. However, if FABP3 was saturated with the monounsaturated LCAC before the detergent was added, it did not cause the dissociation of LCAC (as confirmed by NMR, Figure S49). Thus, this approach allowed us to perform titration with C16:0 and determine the competition with C18:1(n-9)*t*-carnitine (Figure S48). As expected, C16:0 successfully substituted C18:1(n-9)*t*-carnitine in the binding site of FABP3. The resulting enthalpic and entropic components for binding of C16:0 varied only for 0.5–1.2 kcal∙mol^−1^ in comparison to the titration of apo-FABP3 (Table 1, Figure S30).

All thermodynamic parameters were determined by applying the One Set of Sites fitting model, while the competitive binding fitting model failed to provide reasonable data. The *K_D_* values attained by the latter method were extremely overestimated and were 2–3 orders of magnitude smaller than that previously detected (indicating stronger binding). The thermodynamic parameters determined by the one set of sites fitting model varied within 0.6–10 kcal∙mol^−1^ and the largest differences were observed for the displacement of C8:0 with C18:1(n-9)*t*-carnitine (Table S1).

## 3. Discussion

We performed versatile studies on the ability of FABP3 to bind LCACs by applying cytotoxicity assays, NMR, and ITC experiments. In this study, we demonstrate that FABP3 binds LCACs and protects cells against their harmful effects.

Various conditions are known in which limited cardiac FA metabolism induces the accumulation of LCACs. In patients with inherited FA oxidation disorders (FAOD), very high levels of LCACs are observed, especially during fasting or exercise [39,40,41]. Furthermore, in metabolic rearrangements during ischemia and heart failure, oxidative phosphorylation of the FAs is not coupled with acyl group delivery to mitochondria [21,24,25]. Thus, limited mitochondrial FA metabolism leads to an accumulation of LCACs in the mitochondria and cytosol of cardiomyocytes. Elevated levels of LCACs have been shown to inhibit oxidative phosphorylation and induce ROS production in mitochondria [21,24,25]. In addition, LCACs arrest an insulin signaling pathway and pyruvate metabolism and affect ion channels, thus, inducing insulin resistance, cardiac events, arrhythmia, and death in patients [22,23,42]. Our results indicate that FABP3 can protect cells against the accumulation-induced detrimental effects of LCACs up to a certain level.

The protective mechanism of FABP3 depends on its capacity to bind cytotoxic LCACs, which might be influenced by the abundance of FABP3 as well as the intercellular concentration of FAs. A previous study demonstrated that cytosolic fractions of fed or fasted rat hearts exhibit different protective capabilities [21] and this study confirms that FABP3 binds various FAs and their metabolic intermediates. It appears that FABP3 binding capacity is sufficient to handle physiological levels of lipids even if their concentrations are elevated, as occurs in the fasted state. However, increased LCFA and LCAC content, e.g., during myocardial ischemia or starvation of patients with FAOD, exceeds the FABP3 binding capacity and leads to lipotoxicity. Furthermore, in this study, we demonstrated that the binding of LCFAs to FABP3 is stronger than that of the respective LCACs, and in competition experiments, LCFAs can easily substitute ACs in the binding pocket. Thus, an elevated LCFA content in the heart is sufficient to displace LCACs from the binding site of FABP3 and to increase the levels of free LCACs that are detrimental to the survival of cardiac cells. It has been discussed before [25] that LCFA and LCAC exhibit indistinguishable effects in cells and it is possible that LCACs are responsible for the lipotoxicity that is currently attributed to LCFAs.

Protein NMR data clearly indicated that FABP3 potently bound not only FAs but also LCACs [C14:0-, C18:1(n-9)*c*/*t*-, and C20:5(n-3)*c*-carnitines] and C16:0-CoA. While CSPs were highly similar within each class of ligands, two distinctive ligand binding modes were revealed. The first one that was more common for LCFAs showed strong CSPs for the residues deeply inside the binding pocket of FABP3 (Figure 5A–F) as well as for the residues on both sides of the β-barrel (strands β_A–D_ and β_H–J_). On the other hand, the second binding profile that is characteristic of LCAC binding showed smaller changes in the FABP3 β-barrel structure and mainly affected residues of the ligand entry portal (Figure 5G–L). Carnitine alone did not interact with FABP3, confirming that the hydrophobic aliphatic chain but not the carnitine moiety is necessary for the binding of ACs. Apparently, the carnitine moiety prevents long-chain alkyl groups from adopting the U-shape conformation inside the binding pocket of FABP3. Thus, ACs shorter than C14 were unable to bind to FABP3. Unexpectedly, NMR data revealed differences in the binding of CoA and carnitine esters. CSPs determined for C16:0-CoA were weaker but more similar to that observed for the FAs than for LCACs. Hence, the CoA group did not interrupt the binding of the alkyl chain inside the cavity of FABP3, while the carnitine group did.

Our novel ITC approach allowed us to determine binding affinities and thermodynamic parameters for nine FAs of variable alkyl chain lengths and degrees of unsaturation. We evaluated the effect of the detergent additive TX on the binding thermodynamics using C12:0 as a model compound. According to our findings, FAs became slightly less hydrophobic and maintained the enthalpy-driven binding mechanism in the presence of the nonionic detergent. By comparing the thermodynamic data obtained for the C8:0, C10:0, and C12:0 in two conditions (in the presence or absence of the TX supplement), it can be seen that detergent slightly reduced the binding affinities of all three compounds while maintaining relationships within the thermodynamic values. The binding affinities of both saturated (C12:0, C14:0, C16:0, and C18:0) and monounsaturated LCFAs [C18:1(n-9)*c*/*t*] ranged from 0.14 to 0.56 μM, indicating tight binding to FABP3. On the other hand, medium-chain FA, C8:0, and polyunsaturated FA, C20:5(n-3)*c*, were less potent and produced *K_D_* values that were one order of magnitude higher. Thus, FABP3 can bind FAs of variable length, but it is more potent in binding saturated and monounsaturated LCFAs. These results are in good agreement with previously published data [15,43]. It is unclear why C10:0 and C12:0 exhibited similar binding affinities with FABP3, while the competition experiments with C18:1(n-9)*t*-carnitine clearly indicated that C12:0 is a more potent binder. On the other hand, it is possible that the alkyl chain length is more crucial in the competition experiments than the binding affinities. Furthermore, C12:0 was the only FA that showed two binding conformations in the cavity of FABP3 (PDB ID 4TKB) [15]. Thus, it is possible that the *K_D_* value increased due to these conformational rearrangements that disturbed the tight binding.

Determining the thermodynamic parameters for the binding of LCACs to FABP3 was more challenging, as these compounds were less soluble and exhibited low CMC values in comparison to LCFAs [44,45,46,47]. The ligand binding to FABP3 could not be determined through either method for the solubilization of FAs, such as mixed liposomes with DMPC [15] and nonionic detergent additives. We were able to determine protein–ligand binding thermodynamics for LCACs such as C14:0-, C18:1(n-9)*c*/*t*-, and C20:5(n-3)*c*-carnitines and C16:0-CoA, which also showed binding to FABP3 in the NMR assay. The binding affinities of the unsaturated LCACs were by one order of magnitude higher than those of the corresponding LCFAs (*K_D_* of 2.2 and 0.2 μM, respectively), but the *K_D_* of the C14:0-carnitine (11.3 μM) was higher by two orders of magnitude, indicating that FABP3 has a significant preference toward binding of LCFAs and can easily replace LCACs, as was shown by our competitive binding studies.

While all FAs bind to FABP3 with the stoichiometry *N* = 1.0, two different binding mechanisms of LCACs binding to FABP3 with *N* equal to 1.0 and 0.5 were observed. The latter is only possible when two protein molecules are bound by a single ligand molecule. Fournier et al. [48,49] and Paulussen et al. [50] were the first to suggest that FABP3 can bind ACs while triggering protein self-aggregation. However, detailed studies targeting AC binding and their effects on cell viability were not performed. Previous studies [51,52,53] have shown that the FABP3 dimer forms under certain conditions. However, the data are controversial and the precise dimerization mechanism is unclear. The only dimeric structure for FABP3 (PDB ID 1FTP) [54] from the desert locust, *Schistocerca gregaria*, suggests dimerization via portal-to-portal regions. Other experimental data on different FABP isoforms [55,56,57,58,59] show dimerization with different symmetries by interfering mainly around the gap region (Figure 1A, β_D_–β_E_ strands) in a parallel or antiparallel manner. Several structures with dimerization via α_I_–α_II_ were also found [60,61]. Nevertheless, all these proteins were predominantly observed in their monomeric forms, thus, making the dimerization process highly case-specific or induced by ligand binding. Hence, one possible reason for changes in the protein–ligand stoichiometry from 1:1 to 1:2 for the binding of monounsaturated LCAC to FABP3 could be ligand-triggered dimer formation that blocks ligand entry routes to the binding cavity and creates the second binding site. This hypothesis is in good agreement with the competitive binding experiments in which C10:0 replaced only half of C18:1(n-9)*c*-carnitine, confirming the existence of two binding sites with different affinities. According to our NMR data, one of the possible mechanisms is dimerization via the ligand entry portal, in which several crosspeaks have disappeared from the spectra. Thus, this blocking could prevent short- and medium-chain FAs from entering the binding site of FABP3. On the other hand, LCFAs replace LCACs in the binding site of FABP3 and disrupt dimerization.

In conclusion, NMR spectroscopy and our novel ITC assay allowed us to establish the binding affinities of FAs and ACs toward FABP3 and characterize the binding mechanism. Our data indicate that FABP3 potently binds both FAs of variable length and LCACs. Furthermore, FABP3 can protect cells from the harmful effects of LCACs. However, LCFAs can easily substitute LCACs in the binding site of FABP3, thus, making the protective action of FABP3 highly concentration dependent.

## 4. Materials and Methods

### 4.1. Chemistry

**Scheme 1:**
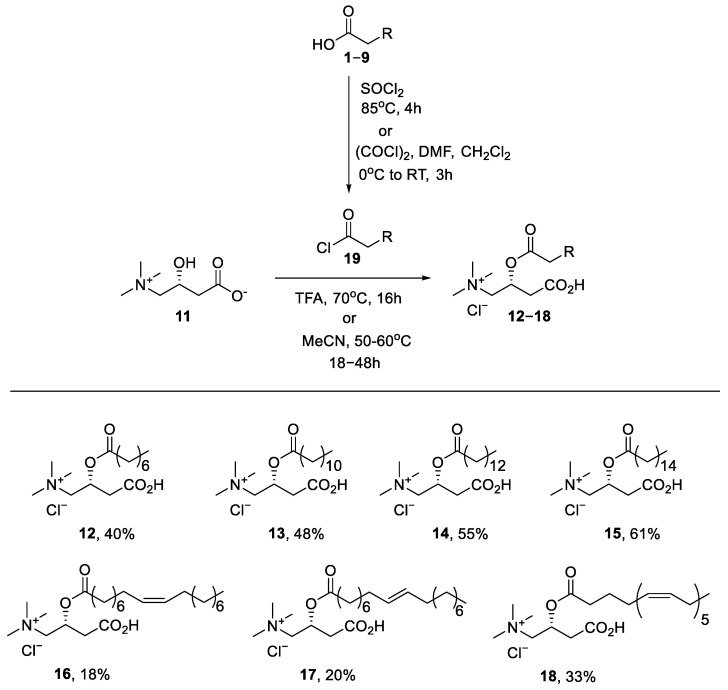
General scheme of the synthesis of saturated **12**–**15** and unsaturated **16**–**18** acylcarnitines (ACs).

All FA (**1**–**9**), C16:0-CoA (**10**), and carnitine (**11**) were commercially available and used without further purification. ACs (**12**–**18**) were synthesized by acylation of carnitine with the respective acyl chlorides [62,63]. Synthetic procedures are described in the supplementary material. Briefly, acyl chlorides **19** were commercially available or obtained by preparation from the corresponding carboxylic acids **1**–**9** in the presence of SOCl_2_ or oxalyl chloride. Acylation of carnitine with **19** in the presence of the trifluoroacetic acid gave saturated acylcarnitines **12**–**15** in 40–61% yield (Scheme 1). Using this protocol C8:0-carnitine (**12**), C12:0-carnitine (**13**), C14:0-carnitine (**14**), and C16:0-carnitine (**15**) were obtained.

In contrast, unsaturated ACs **16**–**18** were synthesized by acylation of carnitine with the corresponding unsaturated acyl chlorides **19** using acetonitrile as the solvent in 18–33% yield (Scheme 1). Thus, C18:1(n-9)*c*-carnitine (**16**), C18:1(n-9)*t*-carnitine (**17**), as well as C20:5(n-3)*c*-carnitine (**18**) were also obtained.

Stock solutions were prepared by dissolution of FAs in 50% (*v*/*v*) ethanol and neutralization by equimolar KOH and dissolution of ACs in DMSO-d_6_, both at 25 mM concentration. C16:0 and C18:0 were heated to 50 °C to achieve complete dissolution. C16:0-CoA was dissolved in D_2_O at the same concentration. The precise concentration of all compound stock solutions was determined by qNMR using the maleic acid internal standard (certified reference material TraceCERT^®^).

### 4.2. Protein Production

The gene coding human FABP3 containing *N*-terminal His-tag and TEV cleavage site in pET-15b vector with *NcoI* and *XhoI* restriction sites was ordered from BioCat (GmbH, Heidelberg, Germany). The resulting amino acid sequence is shown in Figure 1B. The expression plasmid was transformed into *E. coli* BL21(DE3) chemically competent cells and FABP3 was produced by a slightly modified protocol from Matsuoka et al. [15].

Nonlabeled FABP3 was produced by inoculating freshly transformed colonies into a 2 × YT medium. The resulting starter culture was grown overnight at 30 °C and 200 rpm. The next morning, 1% (*v*/*v*) of this night culture was used to inoculate 3 L of 2 × YT. Afterwards, the cells were cultivated at 37 °C and 200 rpm until the OD_600_ reached 0.6–0.8. Recombinant protein expression was induced by the addition of 0.4 mM isopropyl β-*d*-1-thiogalactopyranoside (IPTG) and continued for the next 3 h at the same temperature. Finally, the cells were harvested by centrifugation at 7400× *g* and stored at −20 °C.

^15^N- or ^15^N,^13^C- uniformly labeled FABP3 was produced similarly to a nonlabeled FABP3. Early in the morning, freshly transformed colonies were inoculated into 2 × YT and cultivated for 4–6 h at 37 °C and 200 rpm till the OD_600_ reached 1.0–1.2. Afterwards, 1% (*v*/*v*) of such a starter culture was used to inoculate 20 mL of M9 minimal medium containing ^15^NH_4_Cl and ^13^C-*d*-glucose as the sole sources of nitrogen and carbon. In the case of ^15^N-mono-labeled FABP3 production, nonlabeled *d*-glucose was used. The resulting culture was grown overnight at 37 °C and 200 rpm. The next morning, 1% (*v*/*v*) of the night culture was used to inoculate 1 L of M9 medium. The cells were cultivated at 37 °C and 200 rpm until the OD_600_ reached 0.4. Afterwards, the cultivation temperature was reduced to 20 °C. Protein expression was induced with 0.4 mM IPTG when the OD_600_ reached 0.6–0.8 and continued for the next 20 h under the same conditions. Finally, the cells were harvested by centrifugation at 7400× *g* and stored at −20 °C.

### 4.3. Protein Purification and Delipidation

Both nonlabeled and ^15^N- or ^15^N,^13^C-labeled FABP3 were purified similarly. The thawed cells were resuspended in the lysis buffer (50 mM Tris-Cl pH 8.0, 200 mM NaCl) and disrupted by sonication. The supernatant was harvested by centrifugation at 30,000× *g* for 45 min at 4 °C, filtrated through a 0.22 μm filter, and purified by nickel affinity chromatography on HisTrap™ HP 5 mL column (Cytiva, Marlborough, MA, USA). The column was washed with the binding buffer (50 mM Tris-Cl pH 8.0, 200 mM NaCl, 10 mM imidazole). Protein was eluted with the elution buffer consisting of 50 mM Tris-HCl pH 8.0, 200 mM NaCl, and 500 mM imidazole (Figure S50, lanes 1–7). Fractions containing protein of interest were pooled and concentrated to 3–5 mL on a centrifugal filter unit with a molecular weight cutoff 10 kDa (Merck, Darmstadt, Germany). Concentrated FABP3 was loaded on a size-exclusion chromatography (SEC) HiLoad column 16/600 or 26/600 Superdex™ 75 prep-grade (Cytiva) pre-equilibrated in 20 mM Tris-Cl pH 8.0, 100 mM NaCl (Figure S50, lanes 8–14). Fractions containing FABP3 were pooled and concentrated till 10–20 mg∙mL^−1^.

FABP3 delipidation was performed by refolding the purified protein. For the refolding procedure, concentrated FABP3 was dissolved in 6 M guanidine in 50 mM Tris-Cl pH 8.3, 200 mM NaCl. Afterwards, the protein solution was warmed up to 37 °C and loaded into a prewarmed and pre-equilibrated HisPur™ Ni-NTA (Thermo Fisher Scientific, Waltham, MA, USA) gravity column. Protein was washed with 10 column volumes of 50 mM Tris-Cl pH 8.3, 200 mM NaCl, 6 M guanidine buffer that was warmed to 37 °C. Elevated temperature facilitated unbinding of FAs and other lipids from FABP3 while washing with buffers at room temperature was inefficient. Delipidated protein was eluted with warm 50 mM Tris-Cl pH 8.3, 200 mM NaCl, 6 M guanidine, 500 mM imidazole buffer. Next, FABP3 was diluted to 1 mg∙mL^−1^ with the buffer consisting of 50 mM Tris-Cl pH 8.0, 5 mM dithiothreitol (DTT, Fisher BioReagents™, Pittsburgh, PA, USA), and 6 M guanidine. Protein was refolded by the dialysis against 20 mM Tris-Cl pH 7.5, 5 mM DTT buffer at 4 °C. The amount of apo-FABP3 varied from 70% to 95% between batches. Subsequent ITC binding experiments with FABP3 of different delipidation ranges showed that the remaining fraction of the lipid-bound protein did not affect binding thermodynamics but slightly reduced the stoichiometry, *N*, from 1.0 to 0.7–0.9.

For some experiments, *N*-terminal His-tag was cleaved with TEV protease (prepared in place by protocol from [64]). Cleavage was performed for 3 h at 16 °C. Next, cleaved FABP3 was purified from TEV protease, His-tag, and noncleaved FABP3 on the HisTrap™ HP 5 mL column (Cytiva). The protein of interest was collected from the flow through.

Finally, refolded apo-FABP3 was purified on the SEC HiLoad column 16/600 (Figure S51) or 26/600 Superdex™ 75 prep-grade (Cytiva) pre-equilibrated in KPi. Fractions containing FABP3 were pooled and concentrated until 10–20 mg∙mL^−1^ and either stored at 4 °C (short-term) or aliquoted, fast frozen in liquid nitrogen, and stored at −80 °C (long-term).

Protein concentration was determined using NanoDrop 2000c UV-VIS spectrometer (Thermo Fisher Scientific) by measuring the absorbance at 280 nm and applying theoretical molar extinction coefficient 15,470 M^−1^ cm^−1^ or 13,980 M^−1^ cm^−1^ for His-tagged and cleaved FABP3 versions, respectively.

### 4.4. LCAC Cytotoxicity Assays

PANC-1 (ATCC^®^ CRL-1469™) pancreatic epithelial carcinoma cells were obtained from ATCC^®^ (Manassas, VA, USA). PANC-1 cells were chosen as they are suitable for transfection and are characterized by low endogenous expression levels of FABP3. The cells were grown in Dulbecco’s Modified Eagle’s Medium (DMEM) supplemented with 10% (*v*/*v*) fetal bovine serum (FBS) and 1% (*v*/*v*) penicillin/streptomycin (Pen/Strep) at 37 °C in a humidified incubator with 5% CO_2_. All reagents for cell maintenance were purchased from Thermo Fisher Scientific. For experiments, PANC-1 cells were seeded at density 1 × 10^5^ cells∙mL^−1^ (100 μL per well) in the 96-well plate. Cells were left to attach to the plate overnight. Briefly, before the experiment, PANC-1 cells in 96-well plates were washed with Hanks’ Balanced Salt Solution (HBSS) several times. For all cell experiments, serum-free DMEM media were used.

In order to assess the protective properties of FABP3, we tested the toxicity of C16:0-carnitine over a range of concentrations up to 40 μM for 4 h both in the presence and in the absence of FABP3 (60 μM) in the cell medium. To determine cell viability after incubation with C16:0-carnitine, the 2,5-diphenyl-2H-tetrazolium bromide (MTT) assay was performed [65]. Briefly, the cell media were removed from the wells. A solution of MTT was added to each well at the concentration of 1 mg∙mL^−1^. The plate was incubated for 2 h at 37 °C. Then the MTT solution was removed and 100 μL of isopropyl alcohol was added to each well to dissolve the formazan crystals produced by living cells. The plate was then measured by the Hidex plate reader (Hidex Oy, Turku, Finland).

Next, we tested whether overexpression of FABP3 could result in protective effects regarding the cell viability of the PANC-1 cells. The commercially available FuGENE^®^ HD transfection reagent (Promega Corporation, Madison, WI, USA) was mixed with the FABP3 (NM_010174) mouse tagged ORF clone plasmid (OriGene Technologies, Inc., Rockville, MD, USA) in a 4:1 ratio for transfection according to the manufacturer’s protocol. Transfection was performed for 24 h in 6-well plates. To ensure transfection efficiency, quantitative PCR (qPCR) and Western blot analysis were performed (see Figures S52 and S53). Forward and reverse primer sequences used in qPCR assay are summarized in Table S2. Transfected PANC-1 cells were transferred to 96-well plates and further examined for C16:0-carnitine toxicity at various concentrations as previously described. The obtained results were compared to the effects in native cells.

### 4.5. NMR Spectroscopy

NMR spectra were acquired on the 600 MHz Bruker Avance Neo spectrometer (Bruker Biospin GmbH, Rheinstetten, Germany) equipped with a 5-mm quadruple-resonance CryoProbe (QCI) with z-gradients. The protein samples at 250–350 μM or ~1 mM concentration were prepared for the protein–ligand binding or protein backbone assignment studies, respectively. All experiments were performed in KPi buffer without or with TX and with the addition of 7% (*v*/*v*) D_2_O, 0.03% (*v*/*v*) NaN_3_, and 0.01% (*v*/*v*) SigmaFAST EDTA-free protease inhibitor cocktail to prevent protein degradation. The amount of organic solvent (ethanol or DMSO-d_6_) was below 2% (*v*/*v*) in each experiment.

3D HNCA, 3D HNCO, 3D HN(CA)CO, and 3D CBCA(CO)NH spectra were acquired for the backbone assignment. 3D [^1^H,^1^H]-nuclear Overhauser spectroscopy (NOESY)-^15^N-heteronuclear single-quantum correlation (HSQC) spectrum was acquired using a mixing time of 100 ms and also used in the backbone assignment. 2D ^1^H-^15^N HSQC spectra were acquired for evaluation of the CSPs caused by the lipid binding. Chemical shifts were referenced internally to the residual water signal at 4.70 ppm relative to the 4,4-dimethyl-4-silapentane-1-sulfonic acid (DSS). Backbone assignment was specifically performed for two samples that gave the most distinctive HSQC spectra: apo-protein and FABP3-C18:0. Other complexes were analyzed by analogy and with the help of 3D [^1^H-^1^H]-NOESY-^15^N-HSQC. All spectra were processed in TopSpin v. 4.1.1. (Bruker, Billerica, MA, USA) and analyzed in CARA 1.9.1. [66] software. Residue-specific protein backbone amide assignment was performed based on the published chemical shifts [28]. CSPs, Δ*δ*, were calculated using Equation (1) with the scaling factor, α, which was set to 0.1 [67,68].

(1) $$\Delta \delta =\sqrt{\frac{1}{2}[\delta_{H}^{2}+{\left(\alpha \cdot \delta_{N}\right)}^{2}]},$$

### 4.6. ITC Assay

ITC experiments were carried out on the MicroCal™ iTC200 instrument (Malvern Instruments Ltd., Malvern, UK). Experiments were performed in KPi buffer without and with 0.1% (*v*/*v*) TX supplement. All titrations except for the determination of the heat capacity, Δ*C_p_*, were performed at 25 °C using 20–120 μM protein. Experiments for the determination of the Δ*C_p_* were performed at three different temperatures: 16, 25, and 37 °C. 280 μL of the protein solution in the sample cell were titrated with the ligand solution diluted to 200–1200 μM depending on the enzyme concentration (to achieve a 1:10 or 1:5 ratio). Every titration started with a small 0.3 μL injection introduced to compensate for the diffusion effects. One of the following injection profiles was chosen based on the activity of the ligand and binding heat released: 1.2 μL × 32 or 1.5 μL × 26—all with 120 s time spacing between every injection and 600 rpm. Each ligand was titrated at least two times. The reference baseline consisting of small peaks of identical size (titration of the corresponding ligand into the buffer) was subtracted from the data before fitting to correct for the heat of the ligand dilution. The first injection was excluded from each dataset. The final data were fitted to a theoretical titration curve (one set of sites fitting model) using MicroCal Origin 7 SR4 software (OriginLab, Northampton, MA, USA).

## Figures and Tables

**Table 1:** ITC results of the heart-type fatty acid-binding protein (FABP3) interaction with fatty acids (FAs) and acylcarnitines (ACs) in 20 mM K_2_HPO_4_/KH_2_PO_4_, 50 mM KCl buffer pH 7.6 (KPi) at 25 °C.

#	Compound ^1^	Name	0.1% TX	KD, μM	Δ*G*, kcal·mol^−1^	Δ*H*, kcal·mol^−1^	−*T*Δ*S*, kcal·mol^−1^	*N* ^2^
**1**	C8:0	caprylate	-	1.86 ± 0.05	−7.82 ± 0.02	−8.01 ± 0.09	0.19 ± 0.07	1.0
			+	1.109 ± 0.001	−8.13 ± 0.01	−12.6 ± 0.3	4.5 ± 0.3	1.0
**2**	C10:0	caprate	-	0.252 ± 0.004	−9.01 ± 0.01	−11.09 ± 0.08	2.08 ± 0.08	1.0
			+	0.56 ± 0.02	−8.535 ± 0.007	−13.2 ± 0.3	4.7 ± 0.3	1.0
**3**	C12:0	laurate	-	0.20 ± 0.02	−9.12 ± 0.06	−8.60 ± 0.08	−0.53 ± 0.02	1.0
			+	0.57 ± 0.06	−8.54 ± 0.06	−14.5 ± 0.4	6.0 ± 0.3	1.0
**4**	C14:0	myristate	+	0.3 ± 0.1	−8.9 ± 0.2	−10.9 ± 0.4	1.9 ± 0.2	1.0
**5**	C16:0	palmitate	+	0.14 ± 0.02	−9.34 ± 0.07	−8.7 ± 0.9	−0.6 ± 0.8	1.0
**6**	C18:0	stearate	+	0.7 ± 0.1	−8.34 ± 0.04	−6.4 ± 0.4	−2.0 ± 0.4	1.0
**7**	C18:1(n-9)*c*	oleate	+	0.29 ± 0.03	−8.93 ± 0.06	−10.0 ± 0.4	1.1 ± 0.5	1.0
**8**	C18:1(n-9)*t*	elaidate	+	0.21 ± 0.02	−9.12 ± 0.06	−8.0 ± 0.9	−1.1 ± 0.9	1.0
**9**	C20:5(n-3)*c*	EPA	+	1.6 ± 0.2	−7.90 ± 0.07	−17.4 ± 0.7	9.5 ± 0.8	1.0
**10**	C16:0-CoA	palmitoyl-CoA	-	8.9 ± 1.2	−6.90 ± 0.08	−5.04 ± 0.09	−1.86 ± 0.01	1.0
**14**	C14:0-carnitine	miristoylcarnitine	-	11.3	−6.7	−1.1	−5.7	0.5
**16**	C18:1(n-9)*c*-carnitine	oleoylcarnitine	-	2.21 ± 0.07	−7.72 ± 0.01	−4.6 ± 0.2	−3.1 ± 0.2	0.5
**17**	C18:1(n-9)*t*-carnitine	elaidoylcarnitine	-	2.19 ± 0.04	−7.72 ± 0.02	−4.29 ± 0.06	−3.43 ± 0.08	0.5
**18**	C20:5(n-3)*c*-carnitine	EPA-carnitine	-	14.83 ± 0.08	−6.61 ± 0.03	−2.43 ± 0.09	−4.19 ± 0.06	1.0

^1^ Binding heat effects were not detected for carnitine (**11**), C8:0-carnitine (**12**), C12:0-carnitine (**13**), and C16:0-carnitine (**15**). ^2^ Here, stoichiometry, *N*, is assessed assuming that the protein is 100% active and completely delipidated.

**Table 2:** Comparison of the changes in the heat capacity, Δ*C_p_*, for the heart-type fatty acid-binding protein (FABP3)—ligand complexes.

#	Compound	0.1% TX	Δ*C_p_*, kcal∙mol^−1^∙K^−1^	*R* ^2^	Polar Surface Area, Å^2^ *
**3**	C12:0	−	−0.33 ± 0.01	0.996	40.1
		+	−0.21 ± 0.02	0.981	
**7**	C18:1(n-9)*c*	+	−0.29 ± 0.06	0.964	40.1
**8**	C18:1(n-9)*t*	+	−0.15 ± 0.02	0.988	40.1
**9**	C20:5(n-3)*c*	+	−0.37 ± 0.03	0.994	40.1
**16**	C18:1(n-9)*c*-carnitine	−	−0.09 ± 0.01	0.998	66.4
**17**	C18:1(n-9)*t*-carnitine	−	−0.14 ± 0.01	0.985	66.4
**18**	C20:5(n-3)*c*-carnitine	−	−0.07 ± 0.01	0.975	66.4

* Values computed by Maestro v. 2022-1 software (Schrödinger, LLC: New York, NY, USA) [37].

## Data Availability

The datasets generated and analyzed during the current study are available from the corresponding author on reasonable request.

## Supplementary Materials

The following supporting information can be downloaded at: https://www.mdpi.com/article/10.3390/ijms24065528/s1. Synthetic procedures and analytical data for compounds **12**–**18**; experimental data for NMR and ITC.

## Abbreviations

AC	acylcarnitine
ACBP	acyl-CoA-binding protein
CMC	critical micelle concentration
CSP	chemical shift perturbation
FA	fatty acid
FABP	fatty acid binding protein
FABP3	heart-type fatty acid binding protein
ITC	isothermal titration calorimetry
KPi	potassium phosphate buffer
LCAC	long-chain acylcarnitine
LCFA	long-chain fatty acid
NMR	nuclear magnetic resonance
SASA	solvent-accessible surface area
SEC	size-exclusion chromatography
TX	Triton™ X-100
