# Supplementary material for: Heart-Type Fatty Acid Binding Protein Binds Long-Chain Acylcarnitines and Protects against Lipotoxicity
Source: Int J Mol Sci. 2023 Mar 14;24(6):5528. doi: 10.3390/ijms24065528 (PMC10058761; doi:10.3390/ijms24065528)
Supplement: Supplementary file 1 [file ijms-24-05528-s001.zip › ijms-2266999-supplementary.pdf]

Supplementary Information

Heart-Type Fatty Acid Binding Protein Binds Long-Chain Acylcarnitines and Protects Against Lipotoxicity

Diana Zelencova-Gopejenko [1,2,*], Melita Videja [3,4], Aiga Grandane [5], Linda Pudnika-Okinčica [5], Anda Sipola [6], Karlis Vilks [3], Maija Dambrova [3,4], Kristaps Jaudzems [1], Edgars Liepinsh [3]

Authors Affiliations:

[1] Department of Physical Organic Chemistry, Latvian Institute of Organic Synthesis, Aizkraukles 21, Riga, LV-1006, Latvia; zelencova@osi.lv (D.Z.G); kristaps.jaudzems@osi.lv (K.J.)

[2] Faculty of Materials Science and Applied Chemistry, Riga Technical University, Paula Valdena 3, Riga, LV-1048, Latvia

[3] Laboratory of Pharmaceutical Pharmacology, Latvian Institute of Organic Synthesis, Aizkraukles 21, Riga, LV-1006, Latvia; melita.videja@farm.osi.lv (M.V.); karlis.vilks@cfi.lu.lv (K.V.); maija.dambrova@farm.osi.lv (M.D.); ledgars@farm.osi.lv (E.L.)

[4] Faculty of Pharmacy, Riga Stradins University, Dzirciema 16, Riga, LV-1007 Latvia

[5] Organic Synthesis Group, Latvian Institute of Organic Synthesis, Aizkraukles 21, Riga, LV-1006, Latvia; aiga@osi.lv (A.G.); linda.pudnika@osi.lv (L.P.O)

[6] Laboratory of Membrane active Compounds and β-diketones, Latvian Institute of Organic Synthesis, Aizkraukles 21, Riga, LV-1006 Latvia; anda@osi.lv (A.S.)

**Email of the corresponding author*: zelencova@osi.lv

Table of Contents

Synthetic Procedures....................S2

NMR Spectra of Synthesized Compounds....................S7

Supplementary Figures....................S14

Synthetic Procedures

NMR spectra were recorded at ambient temperature with a 400 MHz Bruker Avance Neo spectrometer (Bruker Biospin Gmbh) equipped with a 5-mm Double-Resonance Broadband CryoProbe with Z-gradients. Chemical shifts are referenced to the residual solvent signal. HRMS (ESI) was performed on a Waters Synapt G2-Si Mass Spectrometer in positive ionization mode. Elemental analysis was performed with a Carlo Erba EA 1108 instrument (CE Instruments Ltd., Hindley Green, UK). The melting point was measured using an OptiMelt melting point apparatus (Stanford Research Systems, Sunnyvale, CA, USA) and is reported uncorrected. Chemicals and reagents and solvents were obtained from commercial suppliers and were used without further purification. Anhydrous dichloromethane was obtained by passing a commercially available solvent through an activated alumina column.

(*R*)-3-Carboxy-2-(octanoyloxy)-*N,N,N*-trimethylpropan-1-aminium chloride (12)

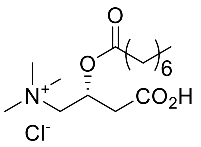

To a solution of L-carnitine (312 mg, 1.94 mmol) in TFA (3 mL) octanoyl chloride (350 mg, 2.15 mmol) was added. The resulting solution was stirred at 70˚C under argon for 16 h. After cooling to room temperature acetone (5 mL) and Et_2O (10 mL) were added. Precipitate was filtered, washed with Et_2O, and dried under vacuum to yield a white solid (250 mg, 40 %). Mp 161–163˚C. $[\alpha]_D^{20}$ –19.8 (*c*=1.0, MeOH). IR (KBr, cm^{-1}): 3441 (O-H), 1733 (C=O). ^1H NMR (400 MHz, DMSO-d_6) δ 5.49 – 5.41 (m, 1H), 3.82 (dd, *J* = 14.3, 8.2 Hz, 1H), 3.73 – 3.67 (m, 1H), 3.12 (s, 9H), 2.76 – 2.63 (m, 2H), 2.38 – 2.24 (m, 2H), 1.57 – 1.46 (m, 2H), 1.32 – 1.18 (m, 8H), 0.89 – 0.82 (m, 3H). ^{13}C NMR (100 MHz, DMSO-d_6) δ 172.1, 170.5, 67.1, 64.9, 53.0, 39.9, 37.2, 33.6, 31.1, 28.3, 24.1, 22.0, 13.9. Anal. Calcd. for $C_{15}H_{30}ClNO_4$: C, 55.63; H, 9.34; N, 4.33. Found: C, 55.88; H, 9.35; N, 4.25.

(*R*)-3-Carboxy-2-(dodecanoyloxy)-*N,N,N*-trimethylpropan-1-aminium chloride (13)

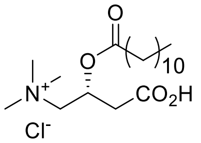

Dodecanoic acid (350 mg, 1.75 mmol) was dissolved in thionyl chloride (2 mL). The clear solution was stirred at 85˚C under argon for 4 hours. The resulting dark solution was cooled to room temperature and evaporated. To the obtained dodecanoic acid chloride a solution of L-carnitine (225 mg, 1.40 mmol) in TFA (2 mL) was added. The resulting dark solution was stirred at 50˚C under argon for 16 h. After cooling to room temperature acetone (5 mL) and Et_2O (10 mL) were added. The white precipitate was filtered and washed with diethyl ether. The crude was recrystallized from isopropanol (10 mL) to yield a white solid (254 mg, 48 %). Mp 162–164 ˚C. $[\alpha]_D^{20}$ –13.8 (*c*=1.1, MeOH). IR (KBr, cm^{-1}): 3450 (O-H), 1735 (C=O). ^1H NMR (400 MHz, DMSO-d_6) δ 5.49 – 5.41 (m, 1H), 3.82 (dd, *J* = 14.4, 8.3 Hz, 1H), 3.71 – 3.63 (m, 1H), 3.12 (s, 9H), 2.74 – 2.63 (m, 2H), 2.38 – 2.24 (m, 2H), 1.57 – 1.46 (m, 2H), 1.32 – 1.17 (m, 16H), 0.91 – 0.81 (m, 3H). ^{13}C NMR (101 MHz, DMSO-d_6) δ 172.1, 170.5, 67.1, 64.9, 53.0, 37.2, 33.6, 31.3, 29.0, 28.9, 28.7, 28.4, 24.1, 22.1, 14.0. Anal. Calcd for $C_{19}H_{38}ClNO_4$: C, 60.06; H, 10.08; N, 3.69. Found: C, 59.69; H, 9.81; N, 3.54.

(*R*)-3-Carboxy-2-(tetradecanoyloxy)-*N,N,N*-trimethylpropan-1-aminium chloride (14)

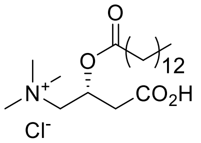

Tetradecanoic acid (500 mg, 2.19 mmol) was dissolved in thionyl chloride (3 mL). The clear solution was stirred at 85˚C under argon for 4 h. The resulting dark solution was cooled to room temperature and evaporated. To the obtained tetradecanoic acid chloride a solution of L-carnitine (282 mg, 1.75 mmol) in TFA (3 mL) was added. The resulting dark solution was stirred at 70˚C under argon for 16 h. After cooling to room temperature Et_2O (10 mL) was added. The white precipitate was filtered and washed with Et_2O. The crude was recrystallized from isopropanol (10 mL) to yield a white solid (395 mg, 55 %). Mp 159–161 ˚C. $[\alpha]_D^{20}$ –14.6 (*c*=1.0, MeOH). IR (KBr, cm^{-1}): 3441 (O-H), 1735 (C=O), 1706 (C=O). ^1H NMR (400 MHz, DMSO-d_6) δ 12.74 (s, 1H), 5.49 – 5.41 (m, 1H), 3.82 (dd, *J* = 14.4, 8.3 Hz, 1H), 3.72 – 3.64 (m, 1H), 2.75 – 2.62 (m, 2H), 2.38 – 2.24 (m, 2H), 1.57 – 1.46 (m, 2H), 1.31 – 1.18 (m, 20H), 0.89 – 0.81 (m, 3H). ^{13}C NMR (100 MHz, DMSO-d_6) δ 172.1, 170.5, 67.1,

64.9, 53.0, 37.2, 33.6, 31.3, 29.05, 29.03, 29.01, 28.99, 28.87, 28.7, 28.4, 24.1, 22.1, 14.0. Anal. Calcd. for $C_{21}H_{42}ClNO_4$: C, 61.82; H, 10.38; N, 3.43. Found: C, 61.55; H, 10.48; N, 3.41.

(*R*)-3-(Palmitoyloxy)-4-(trimethylammonio)butanoate (15)

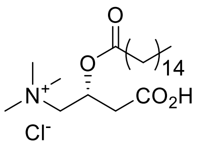

To a solution of L-carnitine (286 mg, 1.77 mmol) in trifluoroacetic acid (3 mL) palmitoyl chloride (0.59 mL, 1.95 mmol) was added dropwise. The resulting solution was stirred at 70 °C in the dark under argon for 4 h and then at room temperature for 16 h. After cooling to room temperature Et_2O (10 mL) was added. The white precipitate was filtered and washed with Et_2O. The crude was recrystallized from isopropanol (10 mL) to yield a white solid (475 mg, 61 %). Mp 157–159 ˚C. $[\alpha]_D^{20}$ –14.4 (*c*=1.00, MeOH). IR (KBr, cm^{-1}): 3488 (O-H), 1748 (C=O). ^{1}H NMR (400 MHz, DMSO–d_6) δ 12.72 (s, 1H), 5.49 – 5.41 (m, 1H), 3.81 (dd, *J* = 14.2, 8.4 Hz, 1H), 3.65 (d, *J* = 14.2 Hz, 1H), 3.11 (s, 9H), 2.74 – 2.63 (m, 2H), 2.38 – 2.24 (m, 2H), 1.58 – 1.46 (m, 2H), 1.32 – 1.17 (m, 24H), 0.89 – 0.81 (m, 3H). ^{13}C NMR (100 MHz, DMSO–d_6) δ 172.1, 170.5, 67.1, 64.9, 53.0, 37.1, 33.6, 31.3, 29.04, 29.00, 28.9, 28.7, 28.4, 24.1, 22.1, 14.0. Anal. Calcd. for $C_{23}H_{46}ClNO_4$: C, 63.35; H, 10.63; N, 3.21. Found: C, 63.11; H, 10.79; N, 3.12.

(*R*)-3-Carboxy-*N,N,N*-trimethyl-2-(oleoyloxy)propan-1-aminium chloride (16)

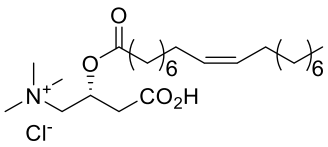

To a solution of oleic acid (2.0 mL, 6.30 mmol) in anhydrous CH_2Cl_2 (30 mL) few drops of anhydrous DMF were added. The solution was cooled to 0 ˚C and oxalyl chloride (1.65 mL, 18.9 mmol) was added dropwise. The reaction mixture was stirred at 0 ˚C for 10 min. then at room temperature for 3h. Volatiles were evaporated. Obtained oleoyl chloride (560 mg, 1.86 mmol) was dissolved in anhydrous MeCN (25 mL) and L-carnitine (0.200 g, 1.24 mmol) was added. The mixture was stirred at 60 ˚C overnight. After cooling to room temperature extra 1.5 equiv. of oleoyl chloride (560 mg, 1.86 mmol) were added. The mixture was stirred at 60 ˚C for 6 h. After cooling to room temperature another extra 1.5 equiv. of oleoyl chloride (560

mg, 1.86 mmol) were added. The resulting mixture was stirred at 60 ˚C for 16 h. The solution obtained was evaporated. The crude was purified by reversed-phase column chromatography on C-18-SH modified silica gel (eluent 0.1% HCl/H_2O-MeCN grad.) to yield a yellow amorphous solid (103 mg, 18 %). $[\alpha]_D^{20}$ = –6.25 (*c*=1.3, $CHCl_3$). IR (neat; cm^{-1}): 3008 (O-H), 1739 (C=O), 1653 (C=C).^{1}H NMR (400 MHz, CD_3OD) δ 5.67 – 5.58 (m, 1H), 5.39 – 5.30 (m, 2H), 3.91 – 3.82 (m, 1H), 3.71 (d, *J* = 13.8 Hz, 1H), 3.21 (s, 9H), 2.83 – 2.69 (m, 2H), 2.39 (t, *J* = 7.4 Hz, 2H), 2.10 – 1.95 (m, 4H), 1.68 – 1.58 (m, 2H), 1.40 – 1.23 (m, 20H), 0.95 – 0.85 (m, 3H). ^{13}C NMR (100 MHz, CD_3OD) δ 174.1, 172.4, 130.9, 130.7, 69.5, 66.2, 54.7, 37.8, 35.1, 33.0, 30.8, 30.6, 30.4, 30.3, 30.2, 28.1, 25.7, 23.7, 14.4. HRMS-ESI (m/z) calcd for $C_{25}H_{48}NO_4$ $[M]^+$ 426.3583, found: 426.3587.

(*R*,*E*)-3-Carboxy-*N*,*N*,*N*-trimethyl-2-(octadec-9-enoyloxy)propan-1-aminium chloride (17)

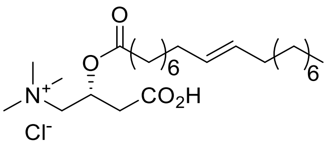

To a solution of elaidic acid (300 mg, 1.06 mmol) in anhydrous CH_2Cl_2 (5 mL) few drops of anhydrous DMF were added. The solution was cooled to 0 ˚C and oxalyl chloride (0.28 mL, 3.19 mmol) was added dropwise. The reaction mixture was stirred at 0 ˚C for 10 min. then at room temperature for 3h. Volatiles were evaporated. Obtained (*E*)-octadec-9-enoyl chloride (320 mg, 1.06 mmol) was dissolved in anhydrous MeCN (4 mL) and *L*-carnitine (154 mg, 0.957 mmol) was added. The mixture was stirred at 60 ˚C for 18 h. Volatiles were evaporated. The crude was purified by reversed-phase column chromatography on C-18-SH modified silica gel (eluent 0.1% HCl/H_2O-MeCN grad.) and recrystallized from MeCN to yield a white solid (98 mg, 20 %). Mp 140–141˚C. $[\alpha]_D^{20}$ = –5.03 (*c*=0.83, $CHCl_3$). IR (neat; cm^{-1}): 3019 (O-H), 1736 (C=O). ^{1}H NMR (400 MHz, CD_3OD) δ 5.66 – 5.58 (m, 1H), 5.44 – 5.33 (m, 2H), 3.87 (dd, *J* = 14.4, 8.5 Hz, 1H), 3.71 (d, *J* = 14.4 Hz, 1H), 3.20 (s, 9H), 2.81 – 2.69 (m, 2H), 2.39 (t, *J* = 7.5 Hz, 2H), 2.05 – 1.93 (m, 4H), 1.69 – 1.57 (m, 2H), 1.42 – 1.24 (m, 20H), 0.95 – 0.85 (m, 3H). ^{13}C NMR (100 MHz, CD_3OD) δ 174.1, 172.4, 131.6, 131.4, 69.5, 66.2, 54.5, 37.7, 35.1, 33.61, 33.58, 33.1, 30.8, 30.7, 30.6, 30.5, 30.23, 30.20, 30.15, 30.0, 25.7, 23.7, 14.4. HRMS-ESI (m/z) calcd for $C_{25}H_{48}NO_4$ $[M]^+$ 426.3583, found: 426.3583.

(*R*)-3-Carboxy-2-(((5*Z*,8*Z*,11*Z*,14*Z*,17*Z*)-icosa-5,8,11,14,17-pentaenoyl)oxy)-*N*,*N*,*N*-trimethylpropan-1-aminium chloride (18)

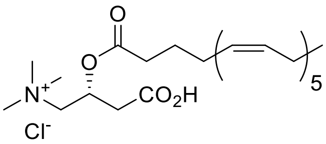

To a solution of eicosapentaenoic acid (500 mg, 1.65 mmol) in anhydrous CH_2Cl_2 (5 mL) few drops of anhydrous DMF were added. The solution was cooled to 0 ˚C and oxalyl chloride (0.43 mL, 4.96 mmol) was added dropwise. The reaction mixture was stirred at 0 ˚C for 10 min. then at room temperature for 3h. Volatiles were evaporated. Obtained eicosapentaenoyl chloride (530 mg, 1.65 mmol) was dissolved in anhydrous MeCN (5 mL) and L-carnitine (266 mg, 1.65 mmol) was added. The mixture was stirred at 50 ˚C for 18 h. Volatiles were evaporated. The crude was purified by reversed-phase column chromatography on C-18-SH modified silica gel (eluent 0.1% HCl/H_2O-MeCN grad.) to yield a yellow amorphous solid (263 mg, 33 %). $[\alpha]_D^{20}$ = –5.73 (*c*=1.5, $CHCl_3$). IR (neat; cm^{-1}): 3025 (O-H), 1732 (C=O). ^1H NMR (400 MHz, MeOD) δ 5.66 – 5.59 (m, 1H), 5.45 – 5.26 (m, 10H), 3.87 (dd, *J* = 14.4, 8.5 Hz, 1H), 3.71 (d, *J* = 14.4 Hz, 1H), 3.20 (s, 9H), 2.91 – 2.69 (m, 10H), 2.44 – 2.38 (m, 2H), 2.19 – 2.04 (m, 4H), 1.75 – 1.66 (m, 2H), 0.97 (t, *J* = 7.6 Hz, 3H). ^{13}C NMR (100 MHz, MeOD) δ 173.9, 172.4, 132.8, 130.1, 129.8, 129.5, 129.23, 129.17, 129.16, 129.08, 128.91, 128.2, 69.4, 66.3, 54.5, 37.7, 34.5, 27.5, 26.6, 26.5, 26.4, 25.6, 21.5, 14.7. HRMS-ESI (m/z) calcd for $C_{27}H_{44}NO_4$ $[M]^+$ 446.3270, found: 446.3275.

NMR Spectra of Synthesized Compounds

^{1}H NMR spectrum of (*R*)-3-Carboxy-2-(octanoyloxy)-*N*,*N*,*N*-trimethylpropan-1-aminium chloride (12).

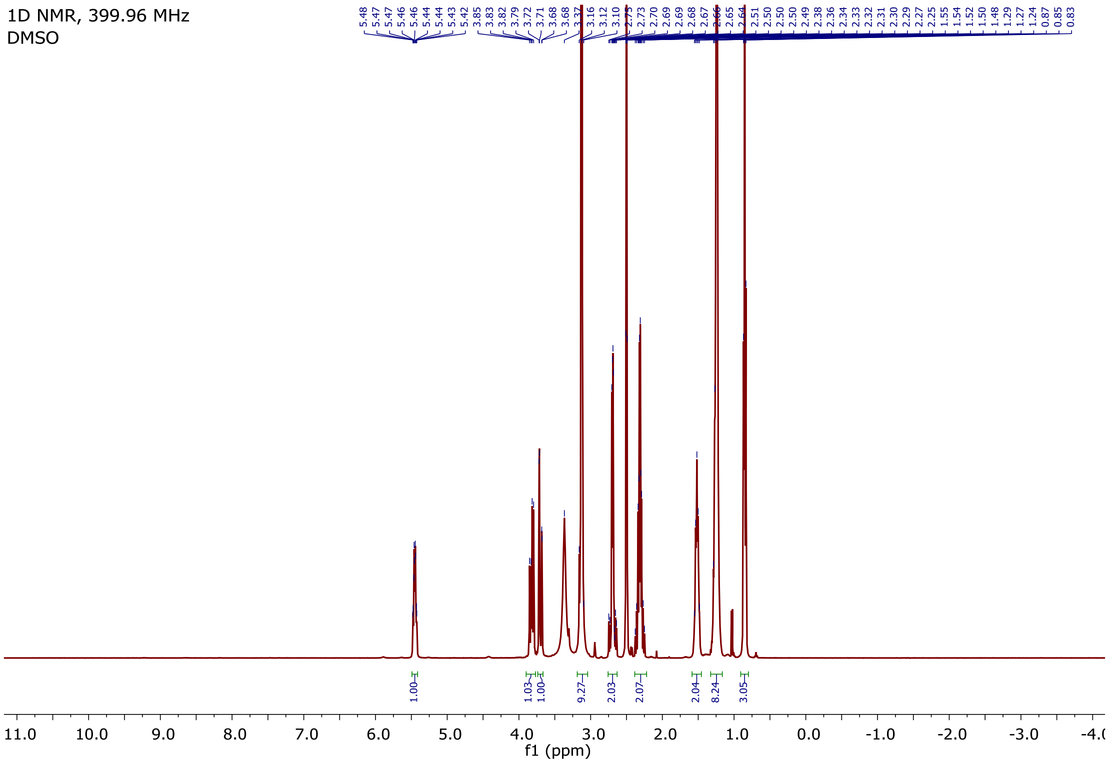

^{13}C NMR spectrum of (*R*)-3-Carboxy-2-(octanoyloxy)-*N*,*N*,*N*-trimethylpropan-1-aminium chloride (12).

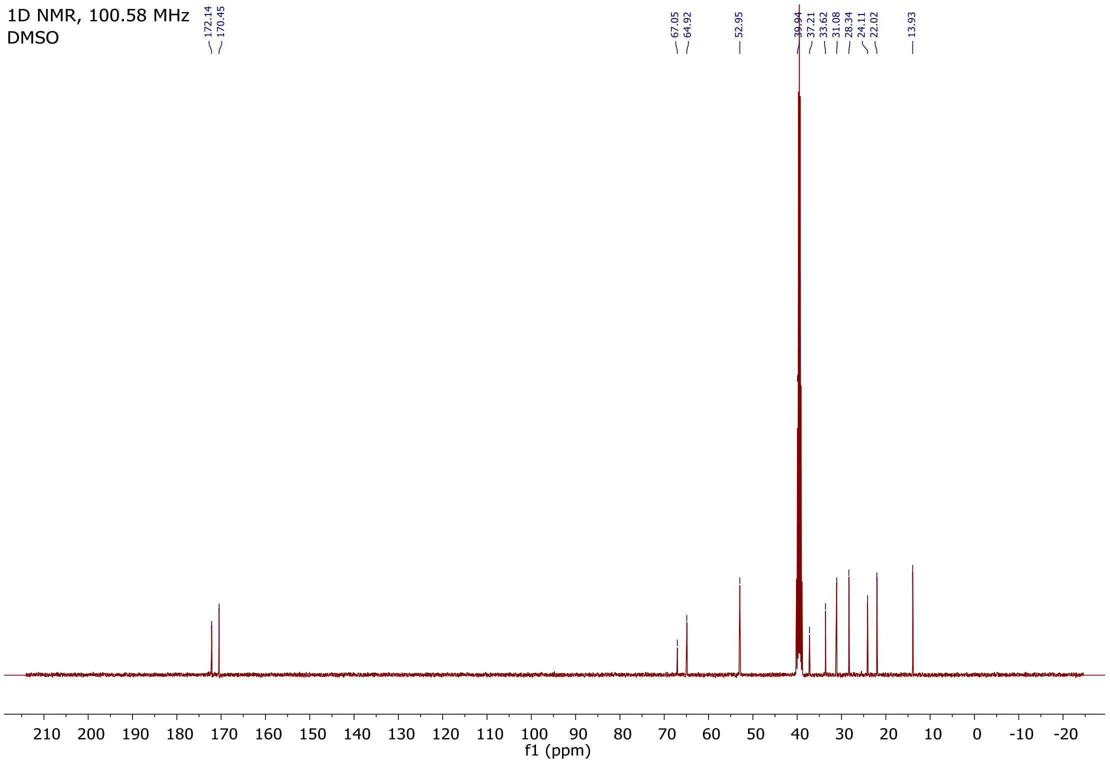

^{1}H NMR spectrum of (*R*)-3-Carboxy-2-(dodecanoyloxy)-*N*,*N*,*N*-trimethylpropan-1-aminium chloride (13).

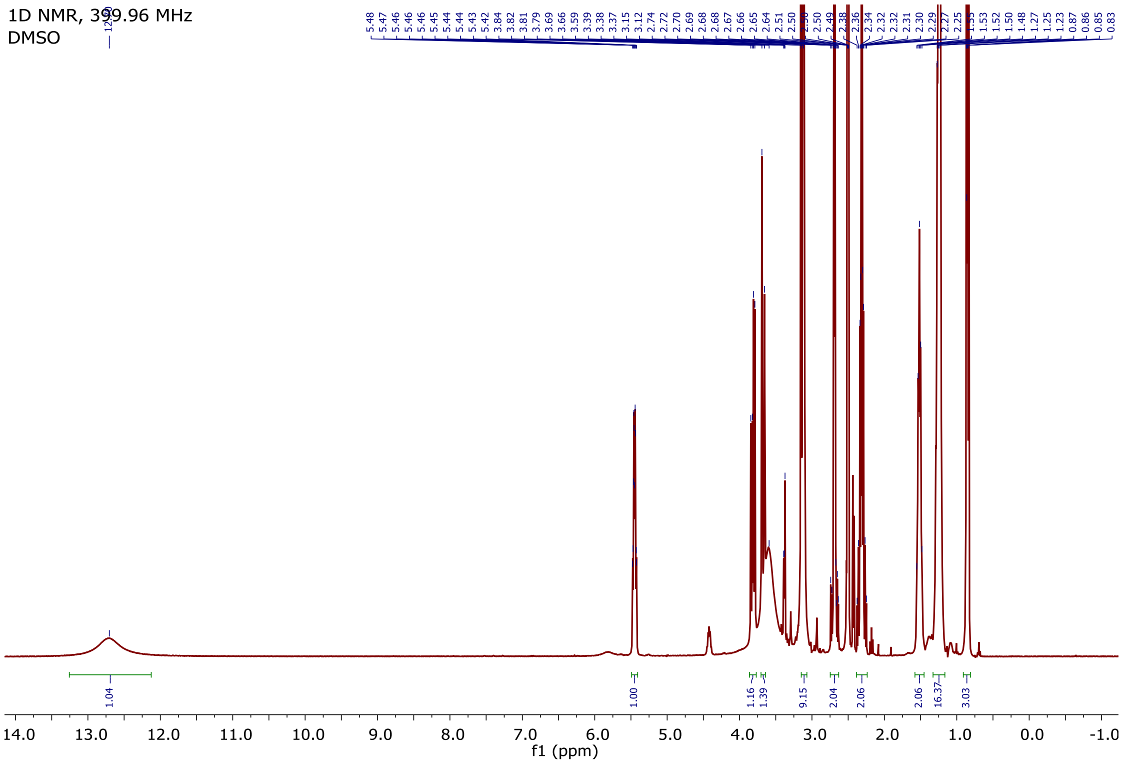

^{13}C NMR spectrum of (*R*)-3-Carboxy-2-(dodecanoyloxy)-*N*,*N*,*N*-trimethylpropan-1-aminium chloride (13).

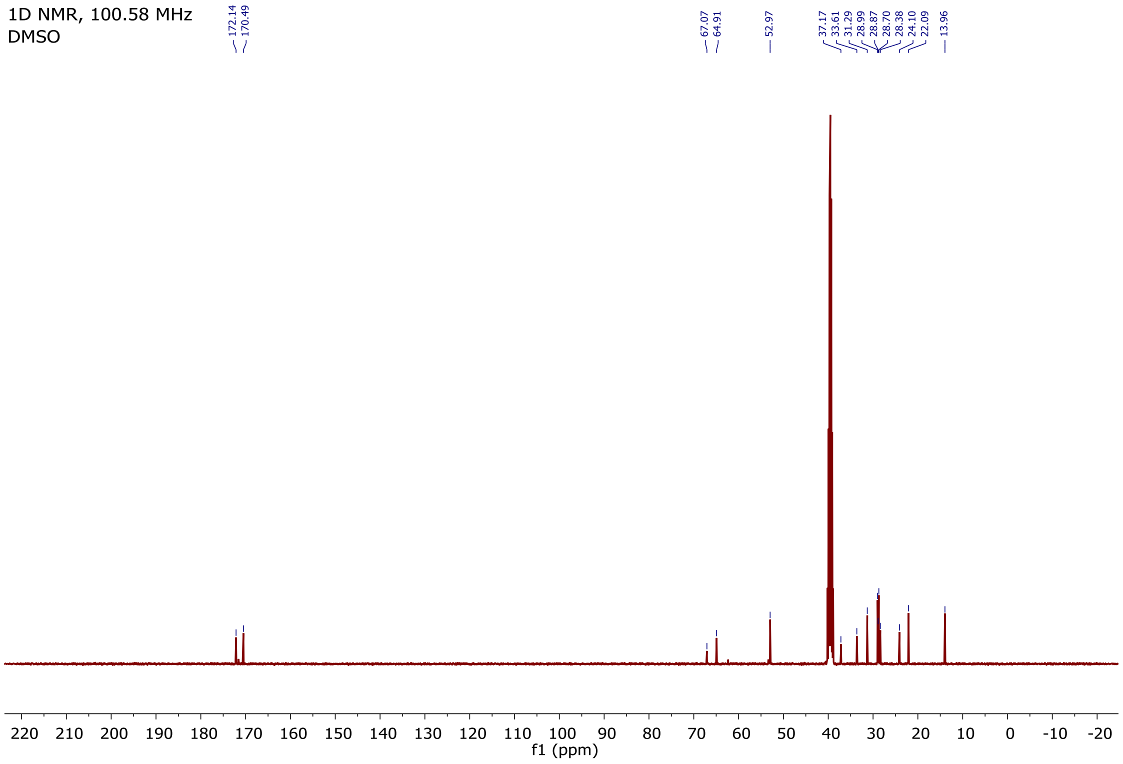

^{1}H NMR spectrum of (*R*)-3-Carboxy-2-(tetradecanoyloxy)-*N*,*N*,*N*-trimethylpropan-1-aminium chloride (14).

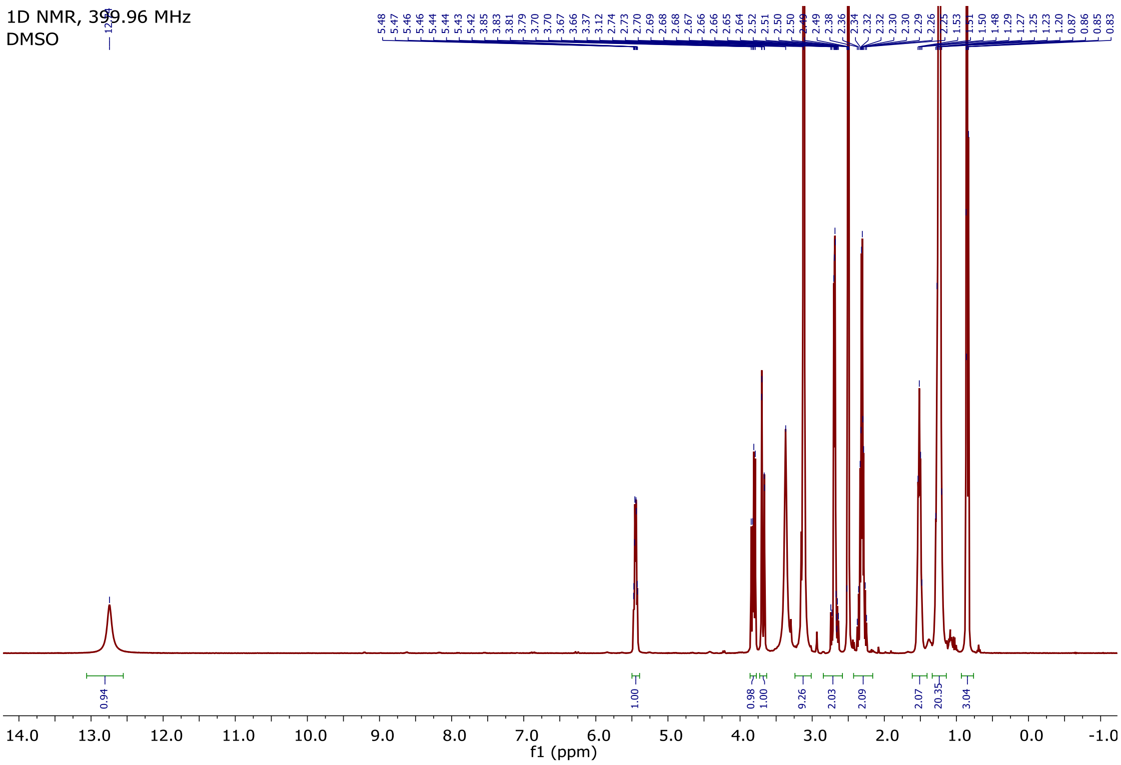

^{13}C NMR spectrum of (*R*)-3-Carboxy-2-(tetradecanoyloxy)-*N*,*N*,*N*-trimethylpropan-1-aminium chloride (14).

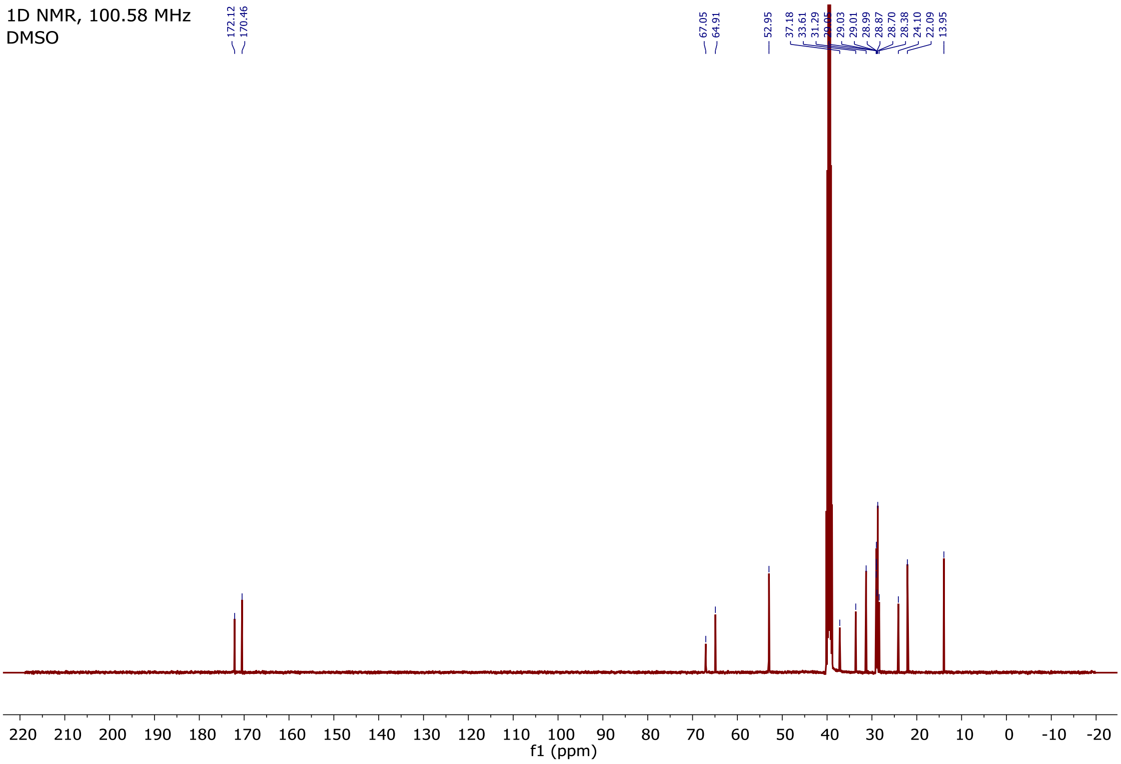

^{1}H NMR spectrum of (*R*)-3-(Palmitoyloxy)-4-(trimethylammonio)butanoate (15).

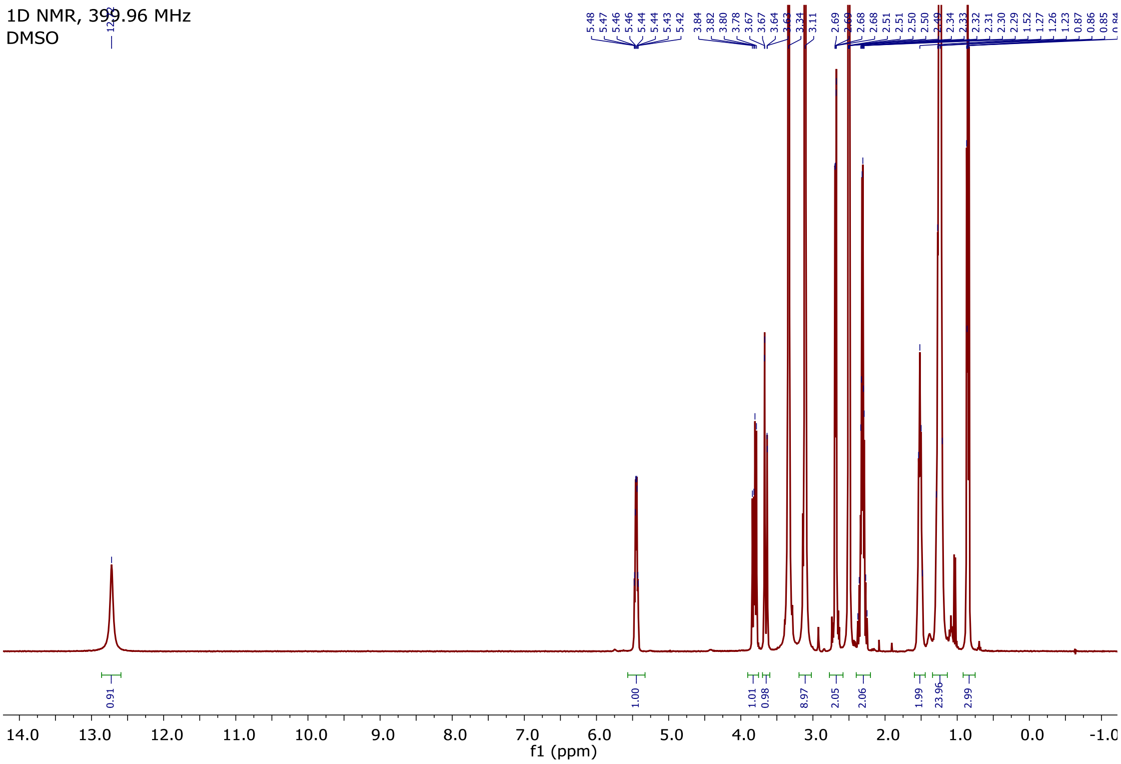

^{13}C NMR spectrum of (*R*)-3-(Palmitoyloxy)-4-(trimethylammonio)butanoate (15).

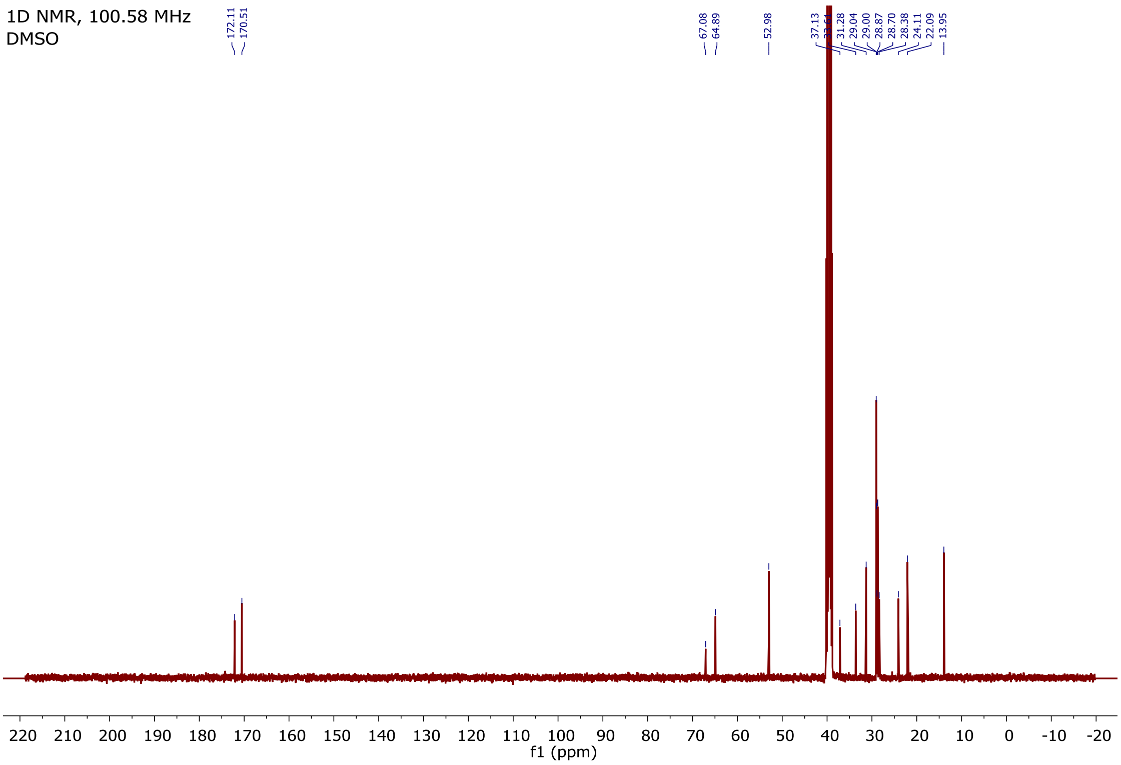

¹H NMR spectrum of (*R*)-3-Carboxy-*N*,*N*,*N*-trimethyl-2-(oleoyloxy)propan-1-aminium chloride (16).

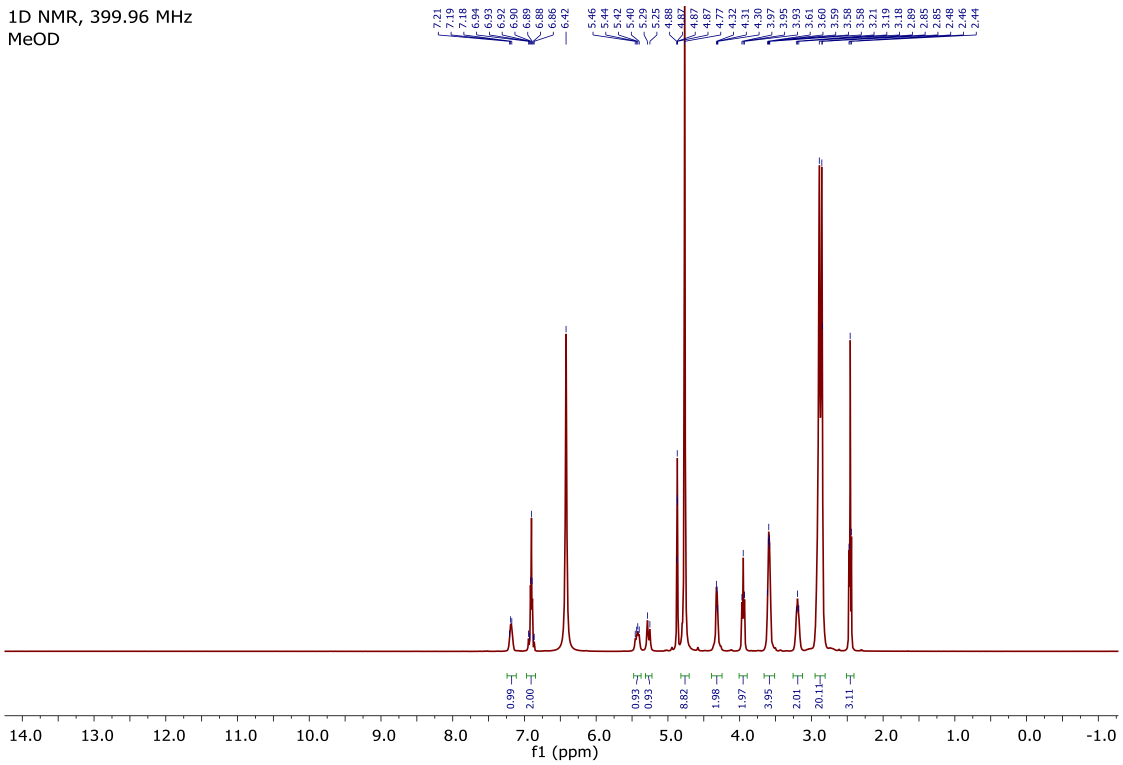

¹³C NMR spectrum of (*R*)-3-Carboxy-*N*,*N*,*N*-trimethyl-2-(oleoyloxy)propan-1-aminium chloride (16).

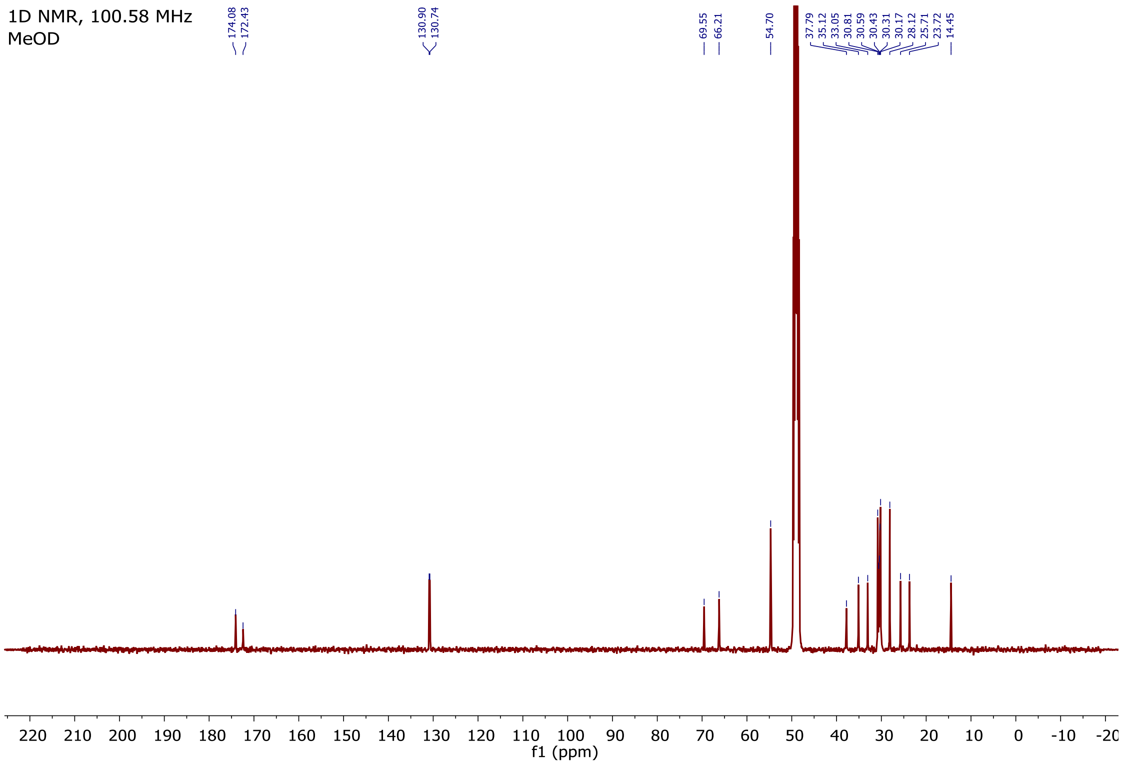

^{1}H NMR spectrum of (*R*,*E*)-3-Carboxy-*N*,*N*,*N*-trimethyl-2-(octadec-9-enoyloxy)propan-1-aminium chloride (17).

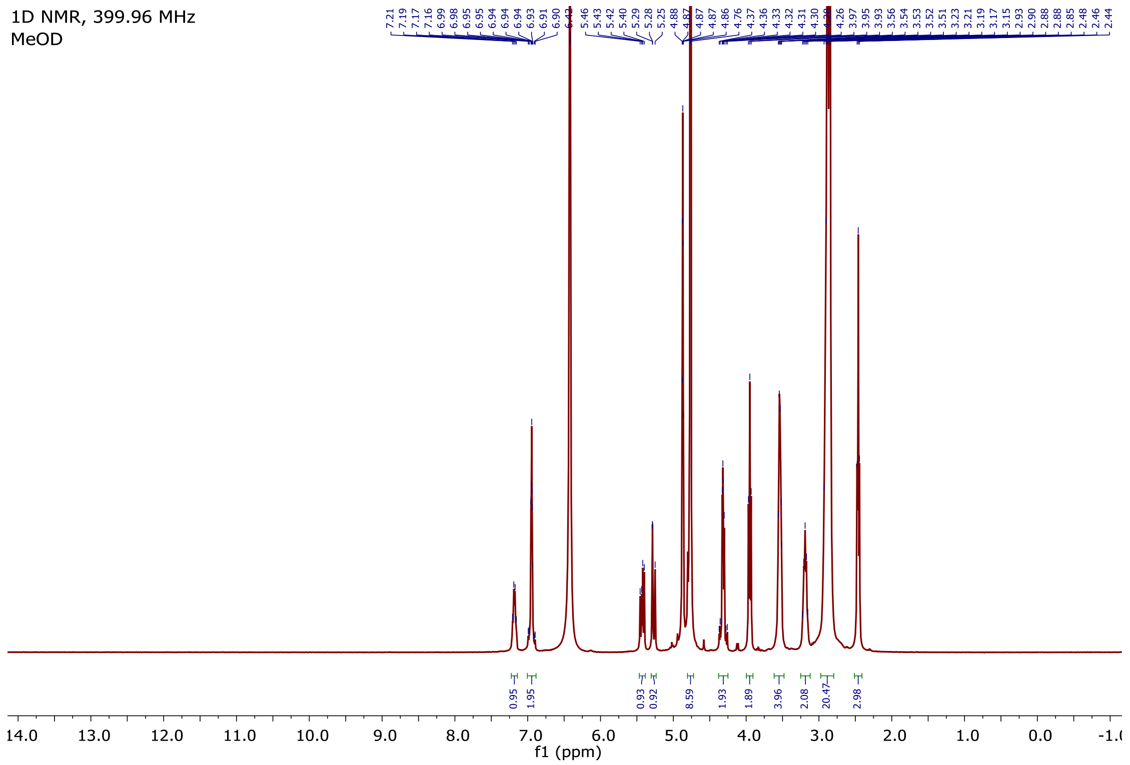

^{13}C NMR spectrum of (*R*,*E*)-3-Carboxy-*N*,*N*,*N*-trimethyl-2-(octadec-9-enoyloxy)propan-1-aminium chloride (17).

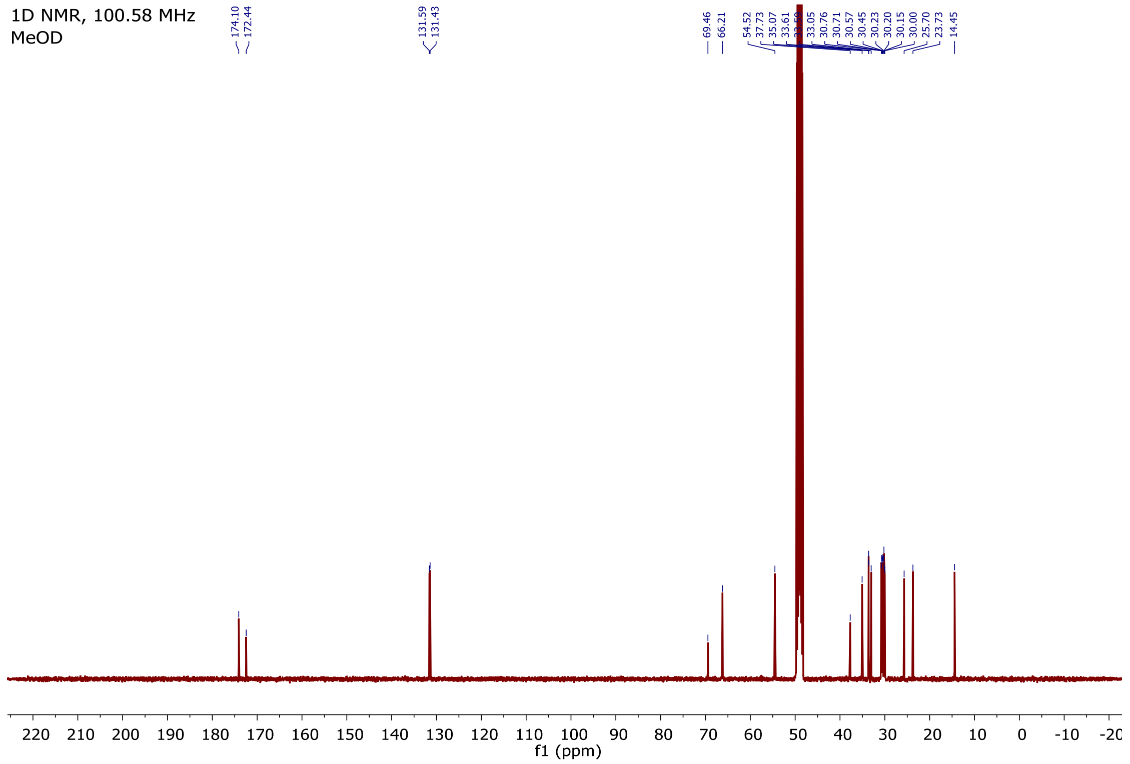

^{1}H NMR spectrum of (*R*)-3-Carboxy-2-(((5*Z*,8*Z*,11*Z*,14*Z*,17*Z*)-icosa-5,8,11,14,17-pentaenoyl)oxy)-*N*,*N*,*N*-trimethylpropan-1-aminium chloride (18).

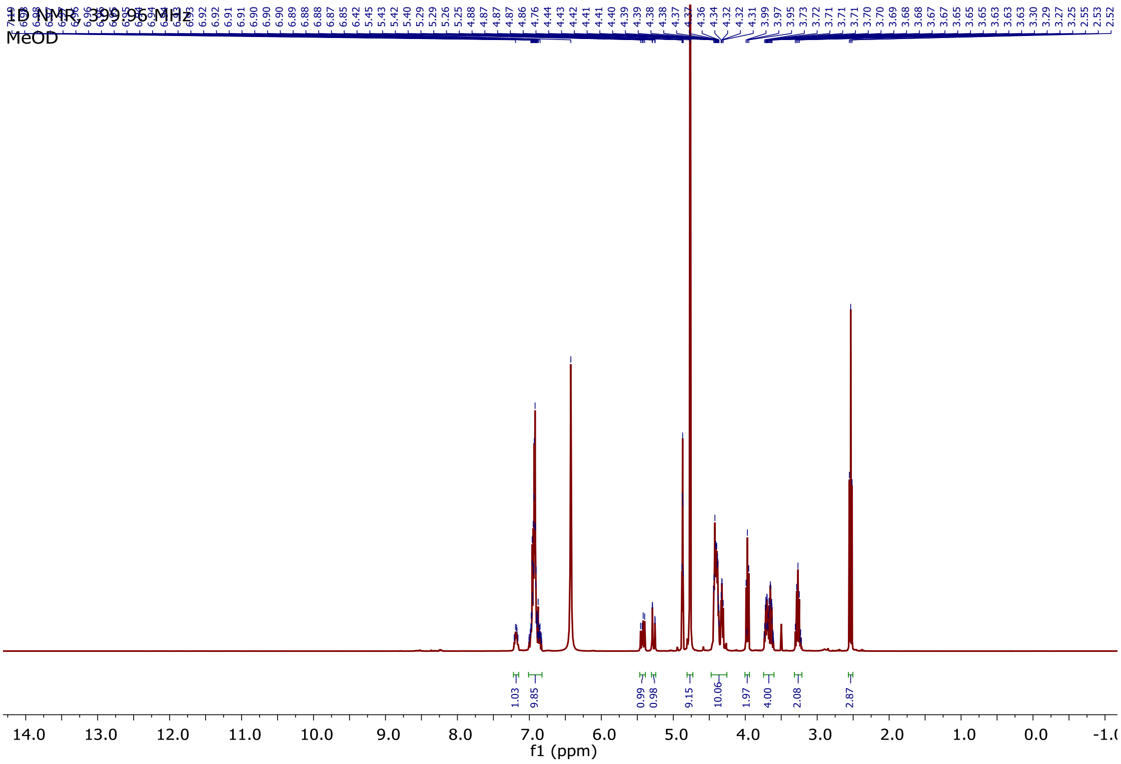

^{13}C NMR spectrum of (*R*)-3-Carboxy-2-(((5*Z*,8*Z*,11*Z*,14*Z*,17*Z*)-icosa-5,8,11,14,17-pentaenoyl)oxy)-*N*,*N*,*N*-trimethylpropan-1-aminium chloride (18).

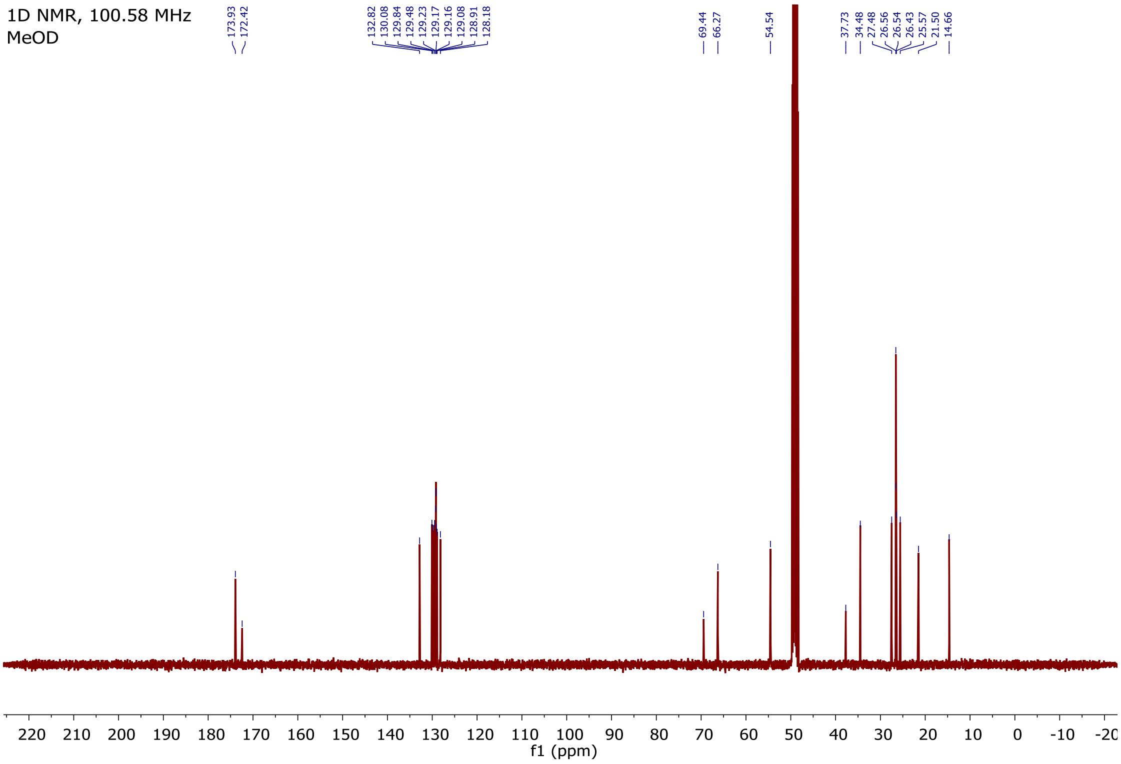

Supplementary Figures

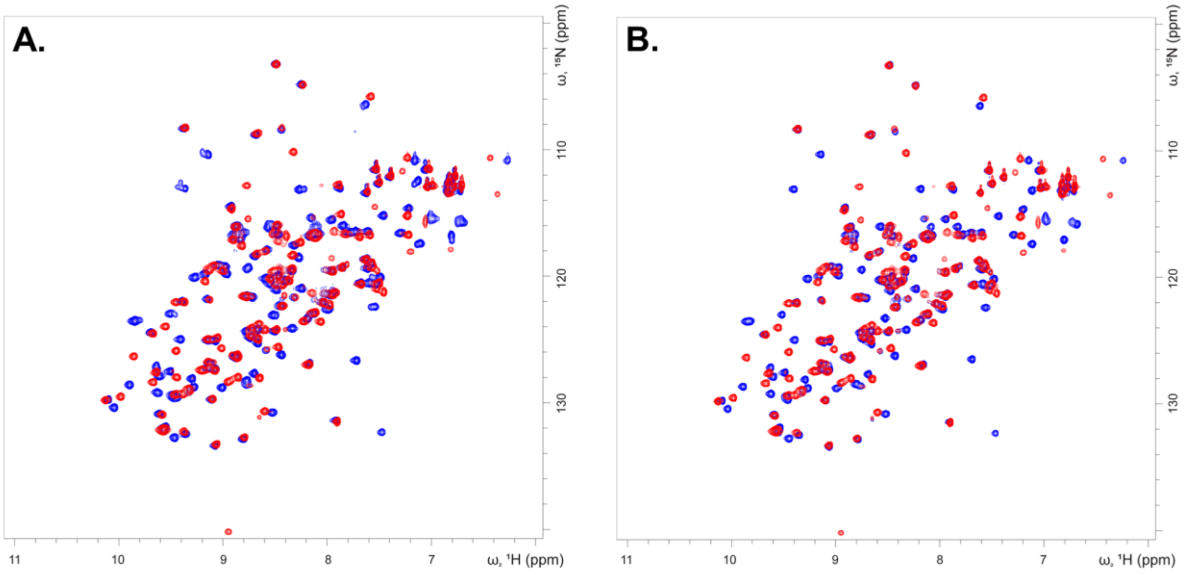

Figure S1. 2D ^{1}H-^{15}N-HSQC spectra of human FABP3 in 20 mM potassium phosphate buffer pH 7.6 with 50 mM KCl. (**A**) Superposition of saturated FABP3 purified from *E. Coli* (blue) and delipidated apo-FABP3 after refolding (red). (**B**) Superposition of refolded apo-FABP3 (red) and FABP3-C16:0 complex (blue).

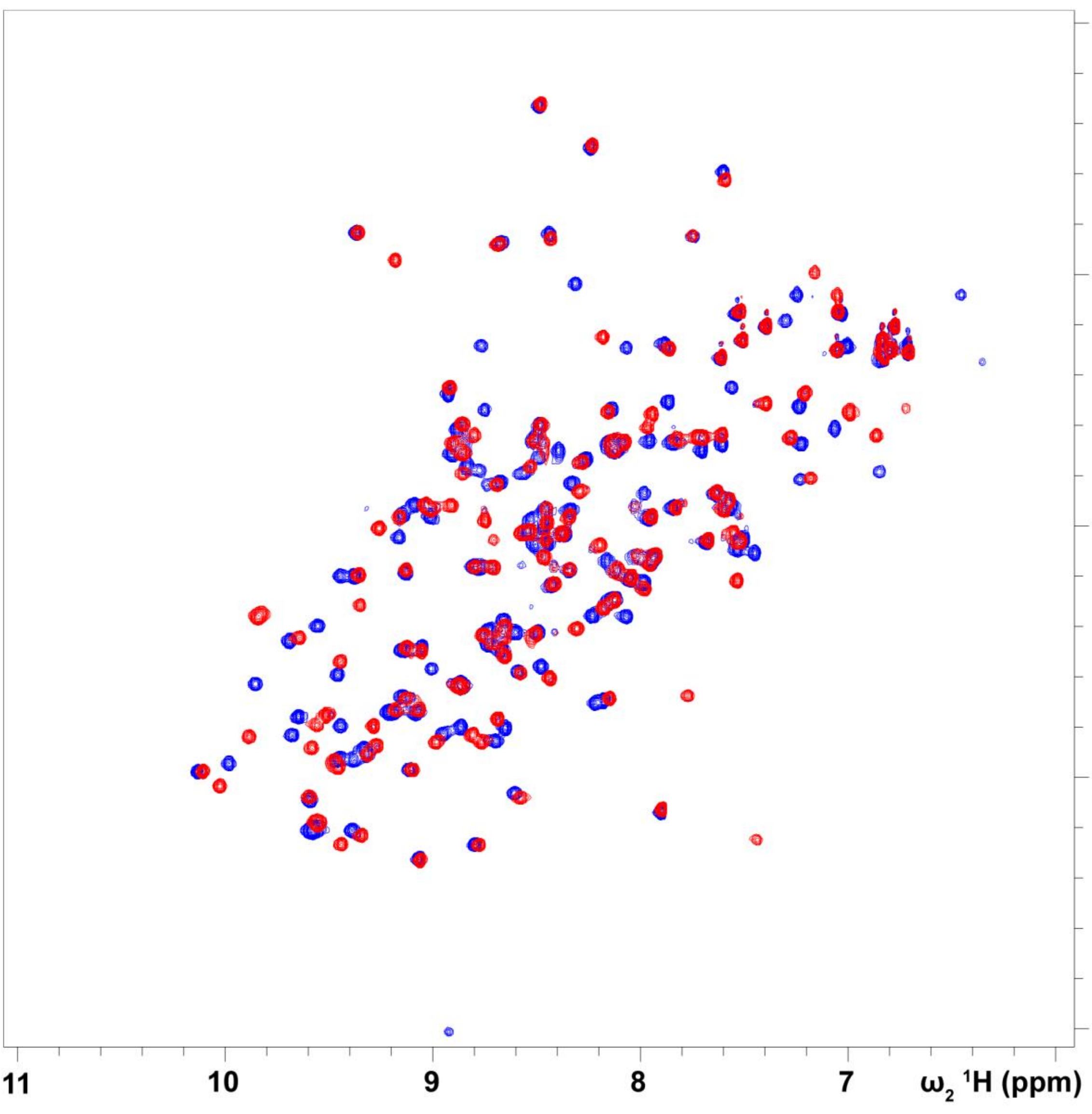

Figure S2. 2D ^{1}H-^{15}N-HSQC spectra of human apo-FABP3 in 20 mM potassium phosphate buffer pH 7.6 with 50 mM KCl. Apo-FABP3 is shown in blue and the FABP3-C8:0 complex – in red.

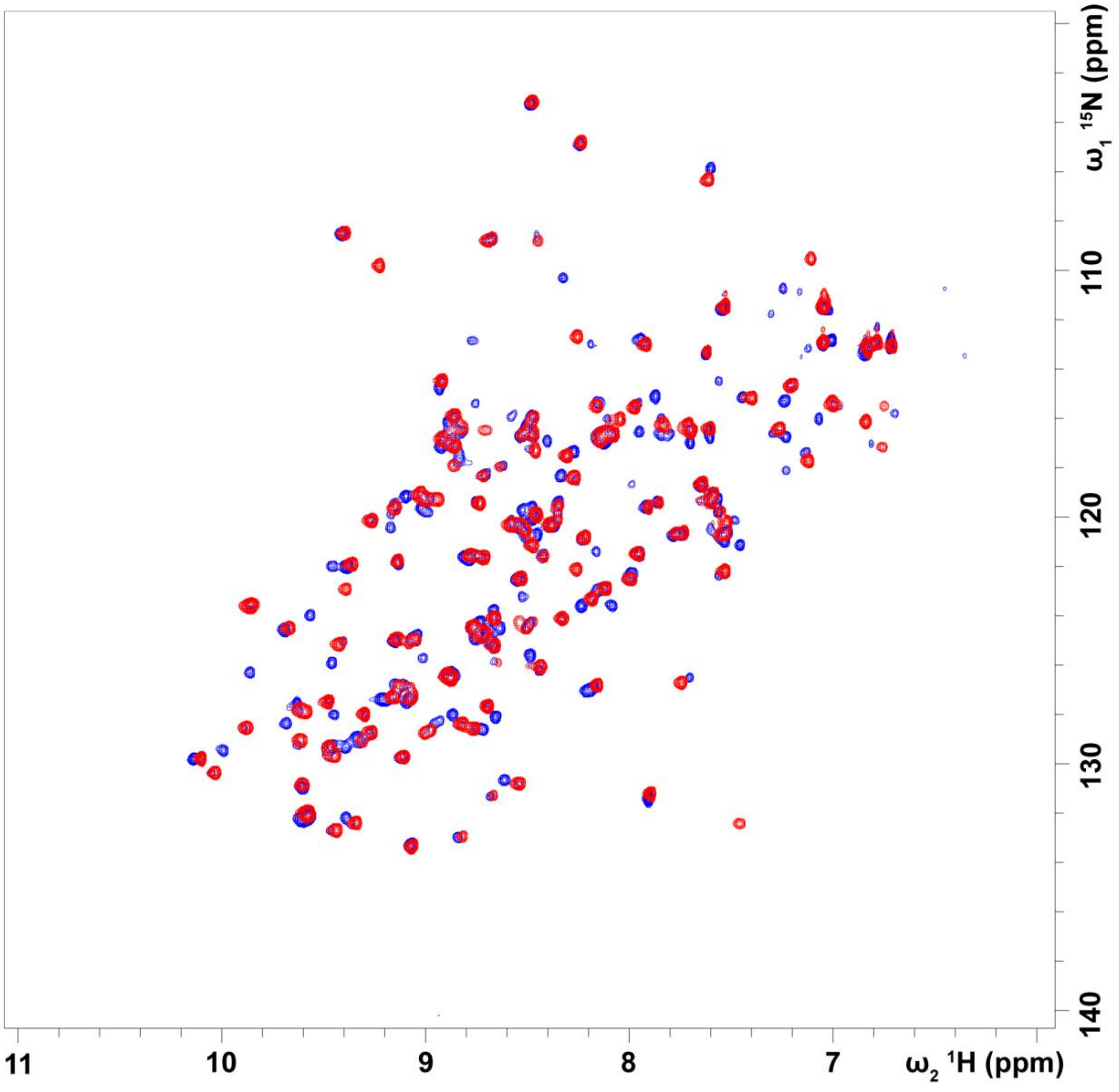

Figure S3. 2D ^1H-^{15}N-HSQC spectra of human apo-FABP3 in 20 mM potassium phosphate buffer pH 7.6 with 50 mM KCl. Apo-FABP3 is shown in blue and the FABP3-C10:0 complex – in red.

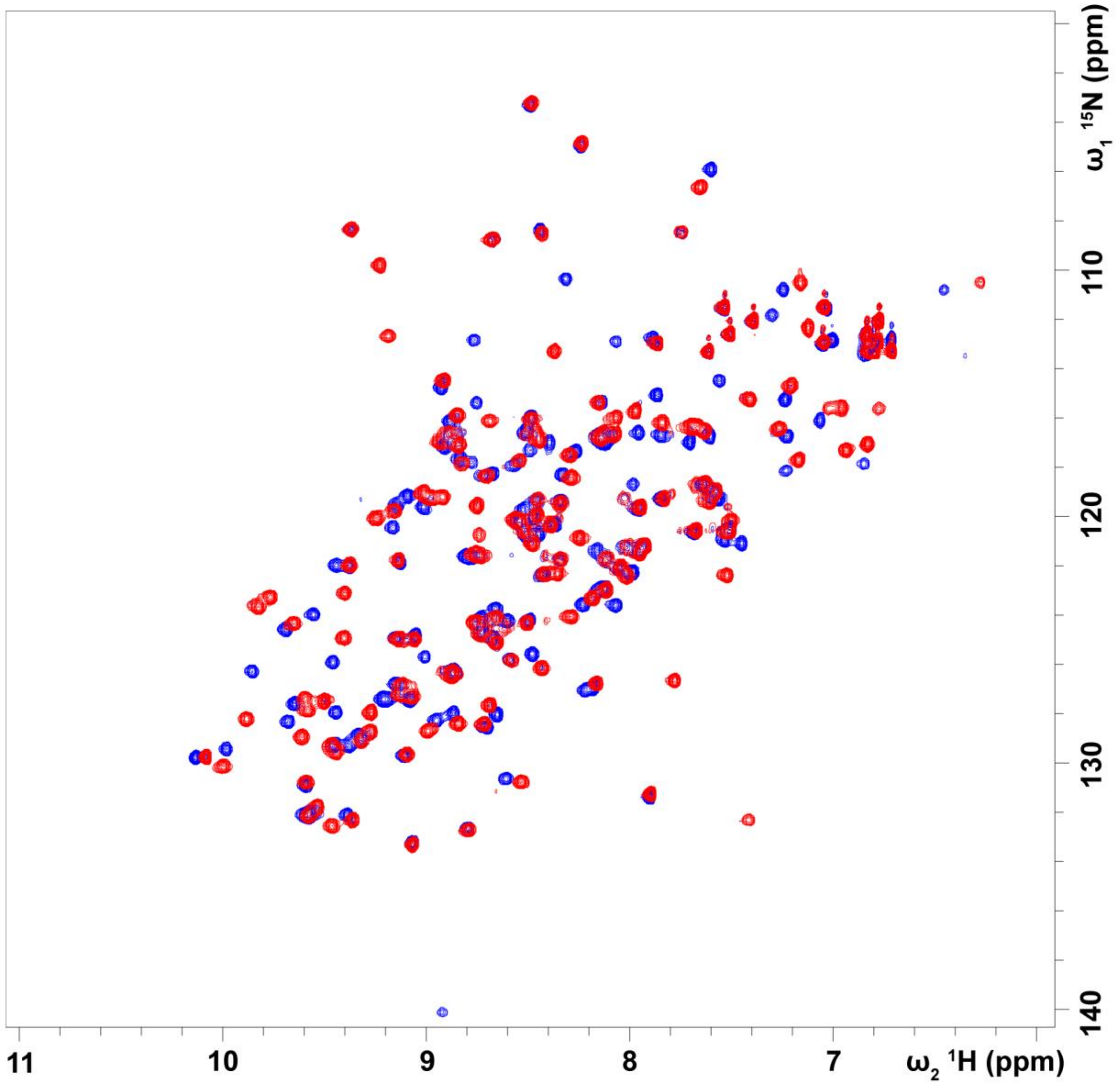

Figure S4. 2D ^{1}H-^{15}N-HSQC spectra of human apo-FABP3 in 20 mM potassium phosphate buffer pH 7.6 with 50 mM KCl. Apo-FABP3 is shown in blue and the FABP3-C12:0 complex – in red.

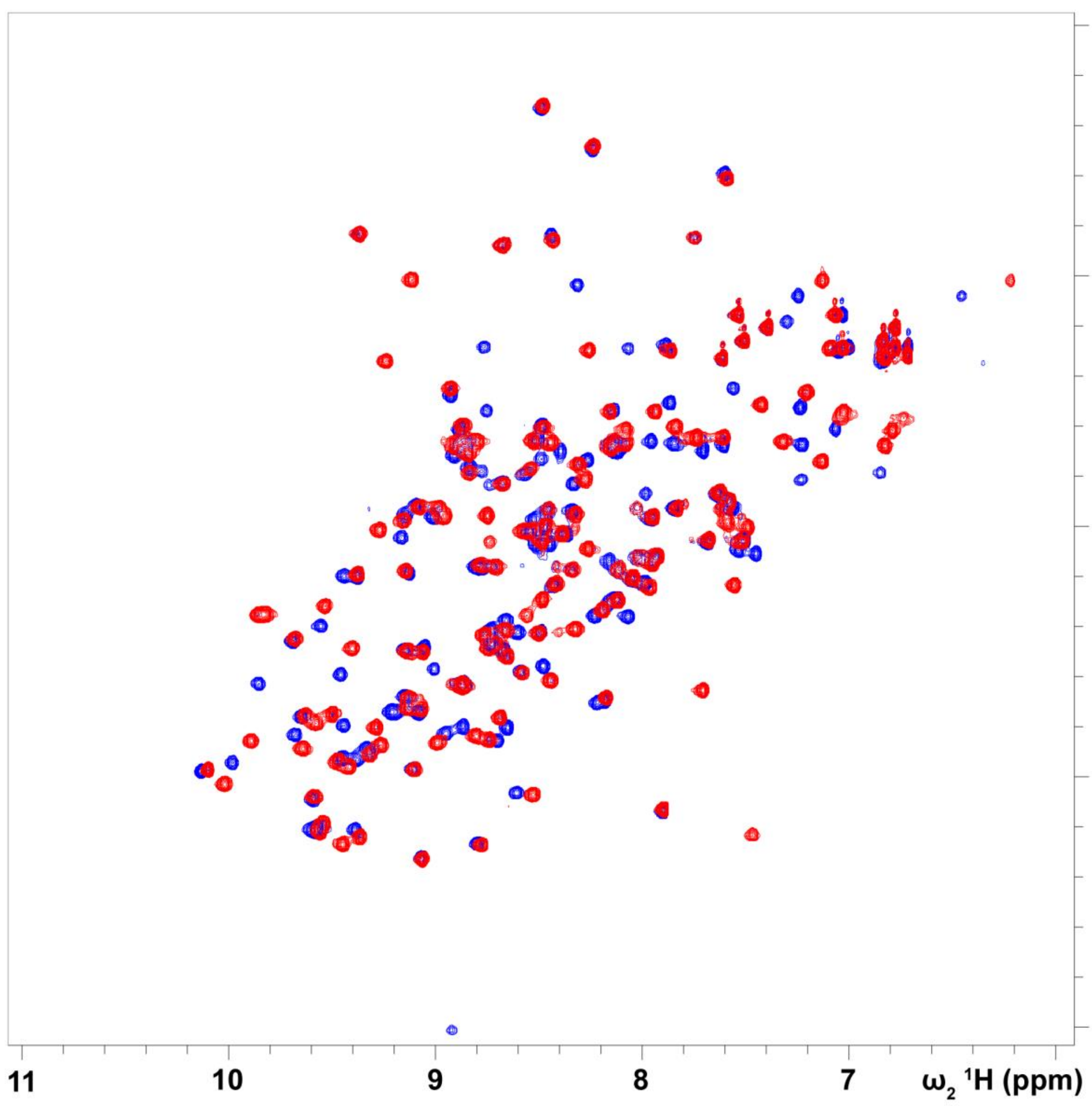

Figure S5. 2D ^{1}H-^{15}N-HSQC spectra of human apo-FABP3 in 20 mM potassium phosphate buffer pH 7.6 with 50 mM KCl. Apo-FABP3 is shown in blue and the FABP3-C14:0 complex – in red.

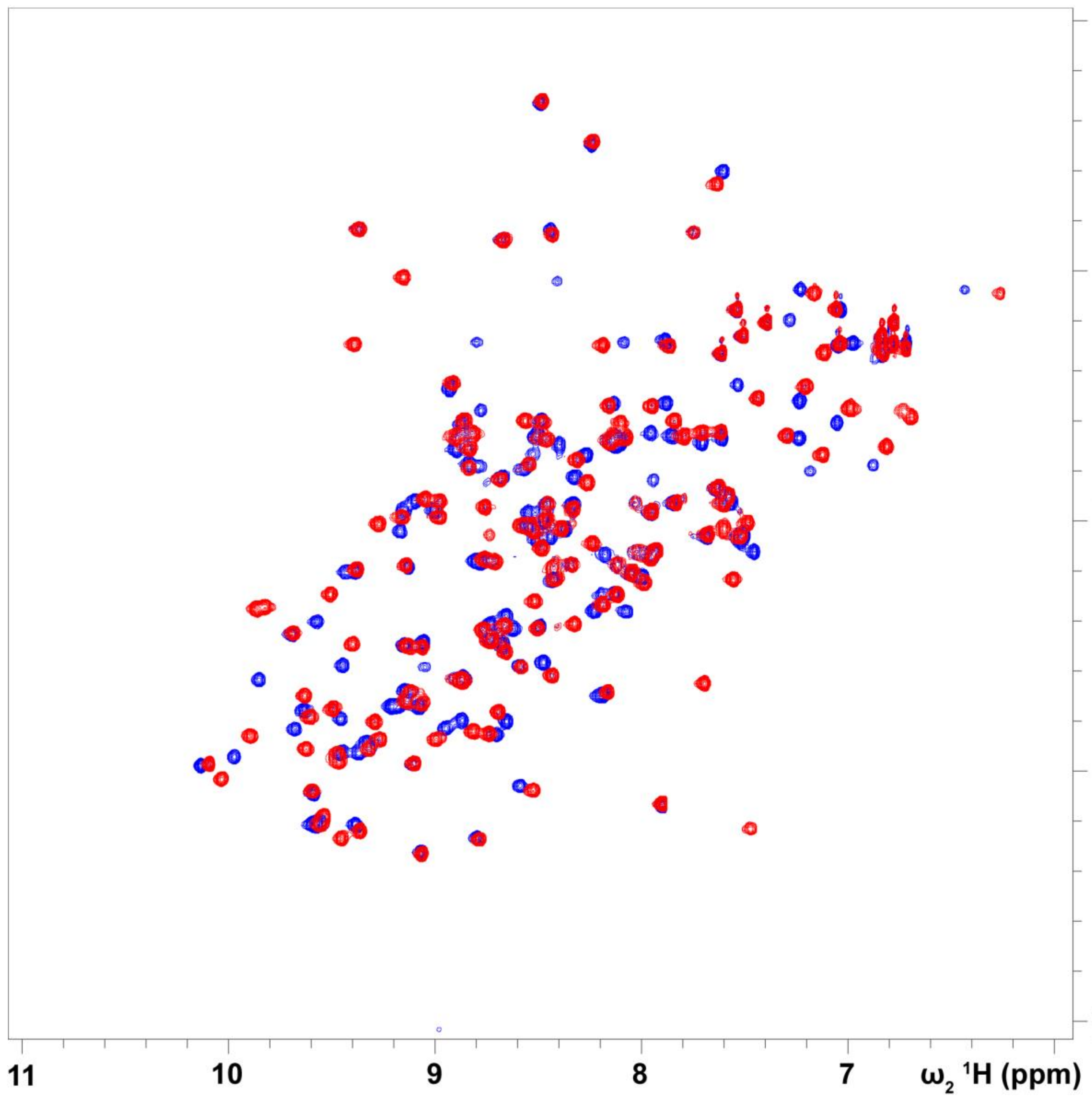

Figure S6. 2D ^{1}H-^{15}N-HSQC spectra of human apo-FABP3 in 20 mM potassium phosphate buffer pH 7.6 with 50 mM KCl. Apo-FABP3 is shown in blue and the FABP3-C16:0 complex – in red.

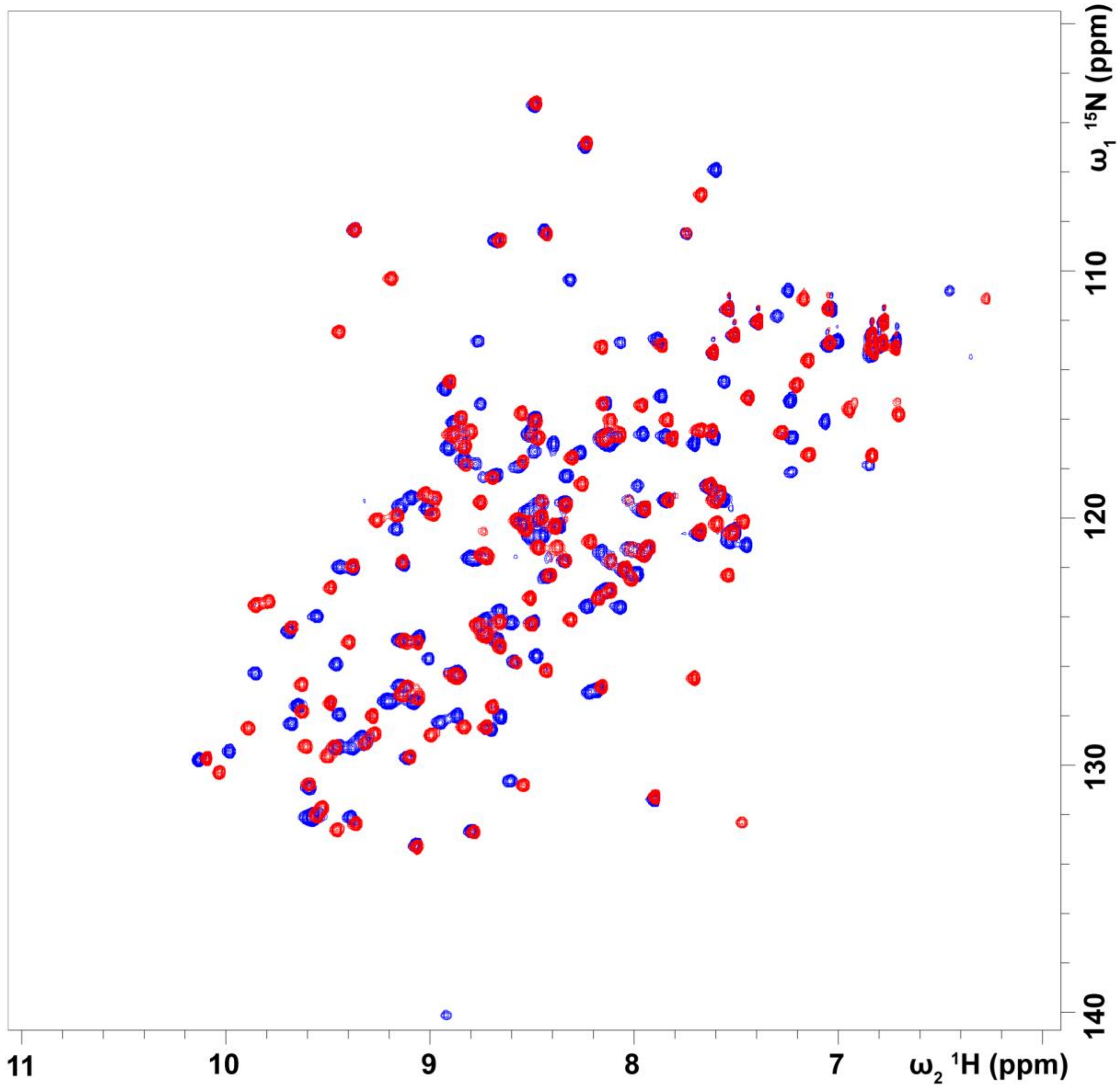

Figure S7. 2D ^{1}H-^{15}N-HSQC spectra of human apo-FABP3 in 20 mM potassium phosphate buffer pH 7.6 with 50 mM KCl. Apo-FABP3 is shown in blue and the FABP3-C18:0 complex – in red.

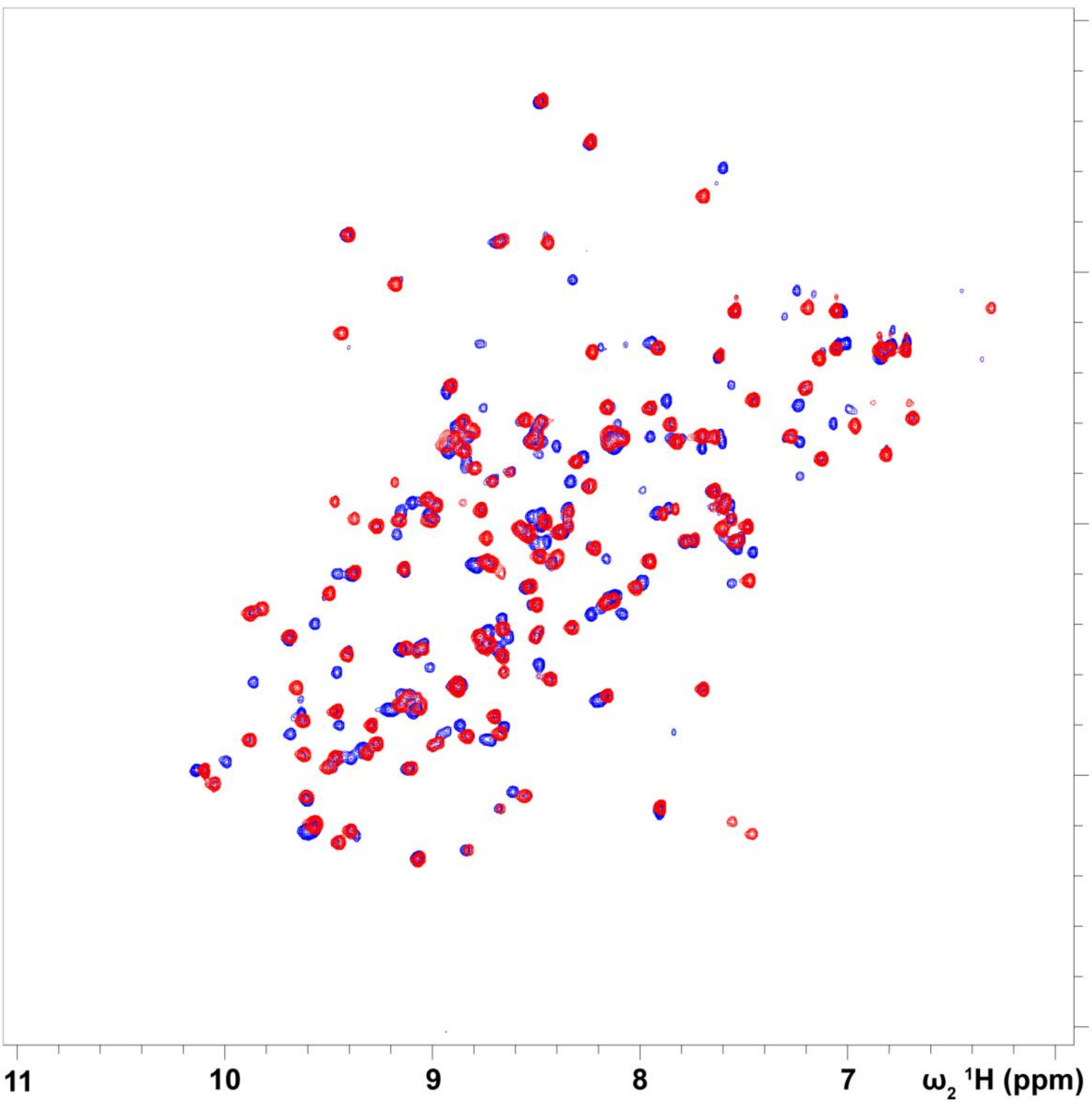

Figure S8. 2D ^{1}H-^{15}N-HSQC spectra of human apo-FABP3 in 20 mM potassium phosphate buffer pH 7.6 with 50 mM KCl. Apo-FABP3 is shown in blue and the FABP3-C18:1(n-9)*c* complex – in red.

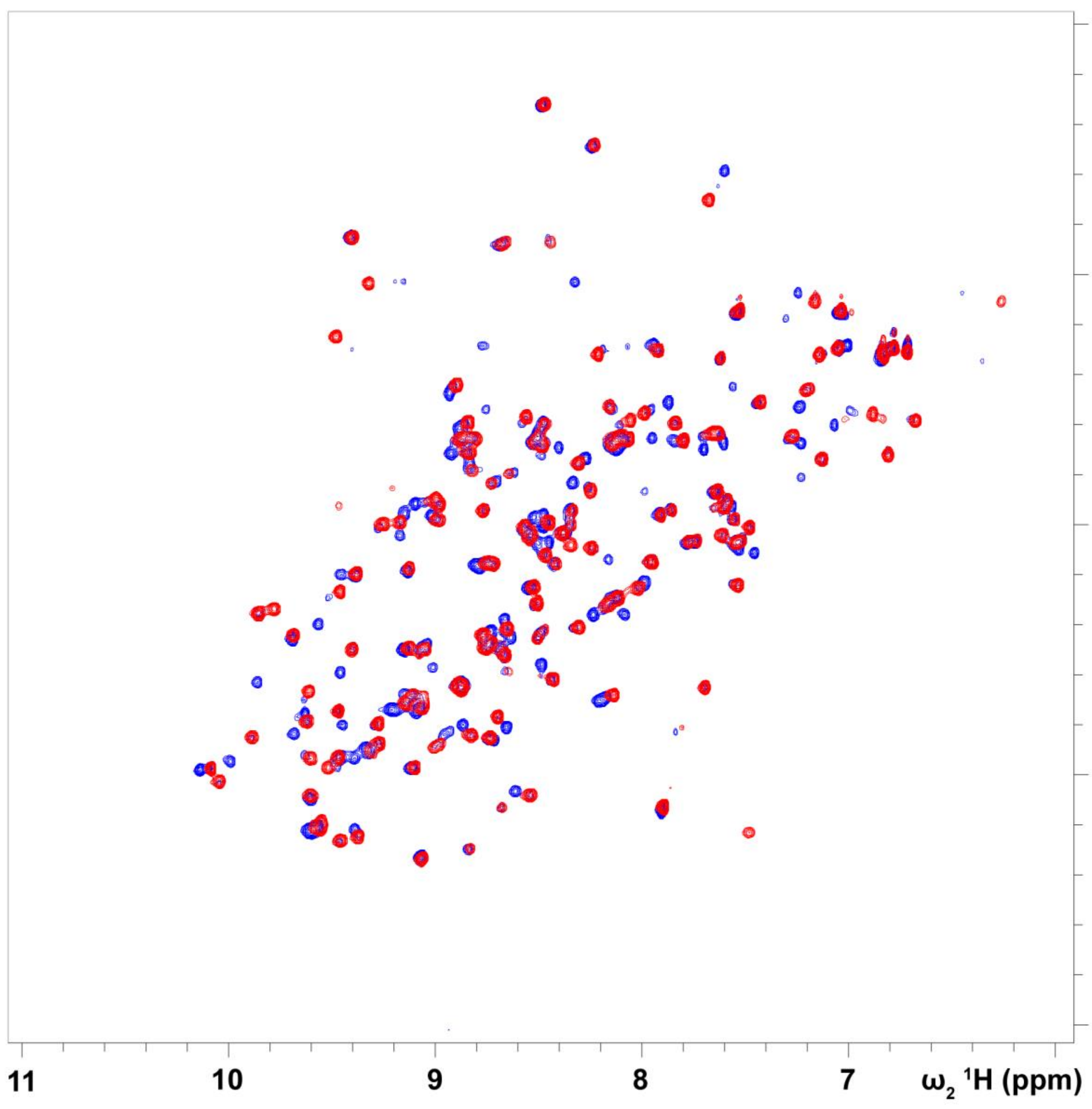

Figure S9. 2D ^{1}H-^{15}N-HSQC spectra of human apo-FABP3 in 20 mM potassium phosphate buffer pH 7.6 with 50 mM KCl. Apo-FABP3 is shown in blue and the FABP3-C18:1(n-9)*t* complex – in red.

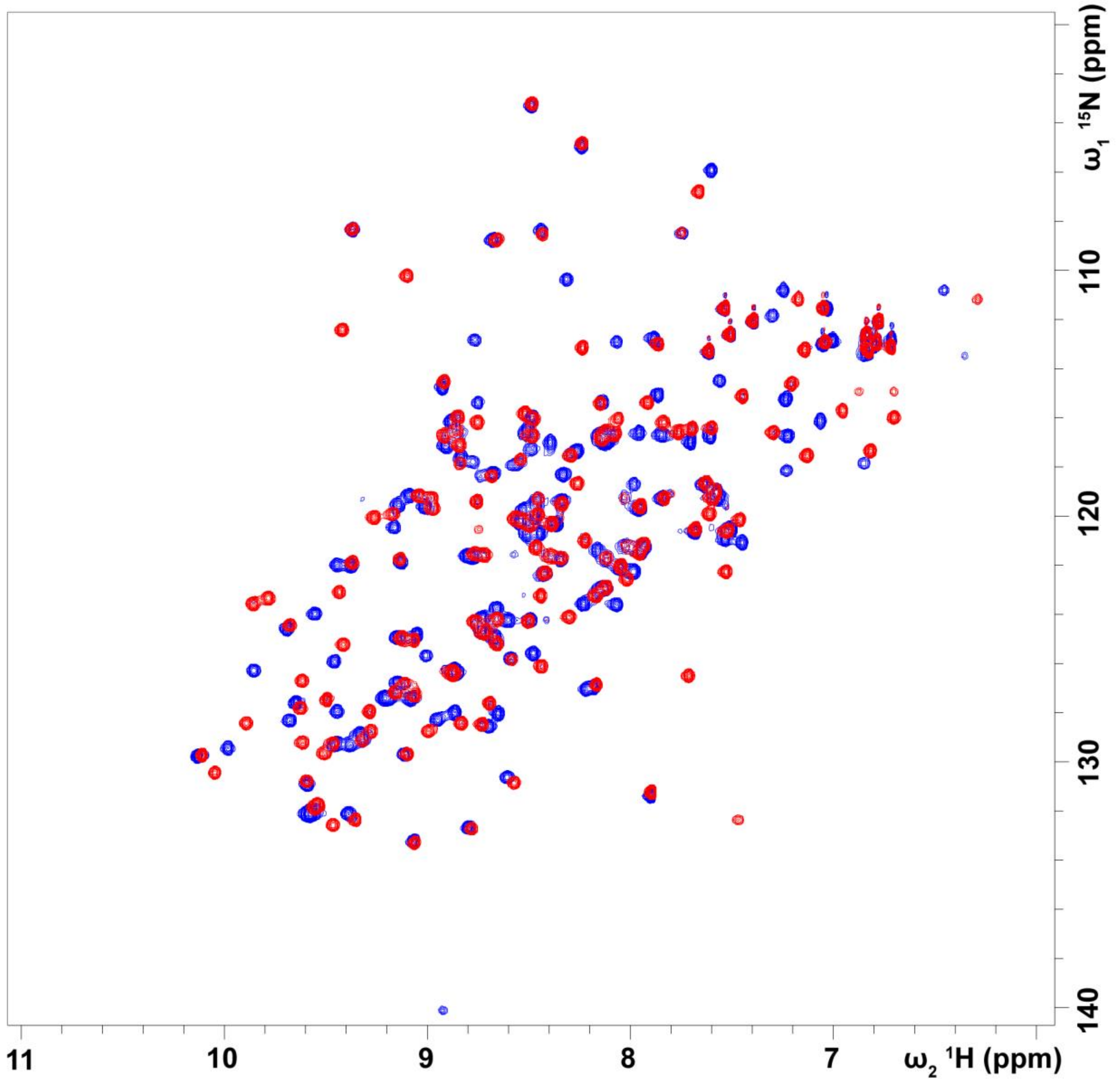

Figure S10. 2D ^{1}H-^{15}N-HSQC spectra of human apo-FABP3 in 20 mM potassium phosphate buffer pH 7.6 with 50 mM KCl. Apo-FABP3 is shown in blue and the FABP3-C20:5(n-3)*c* complex – in red.

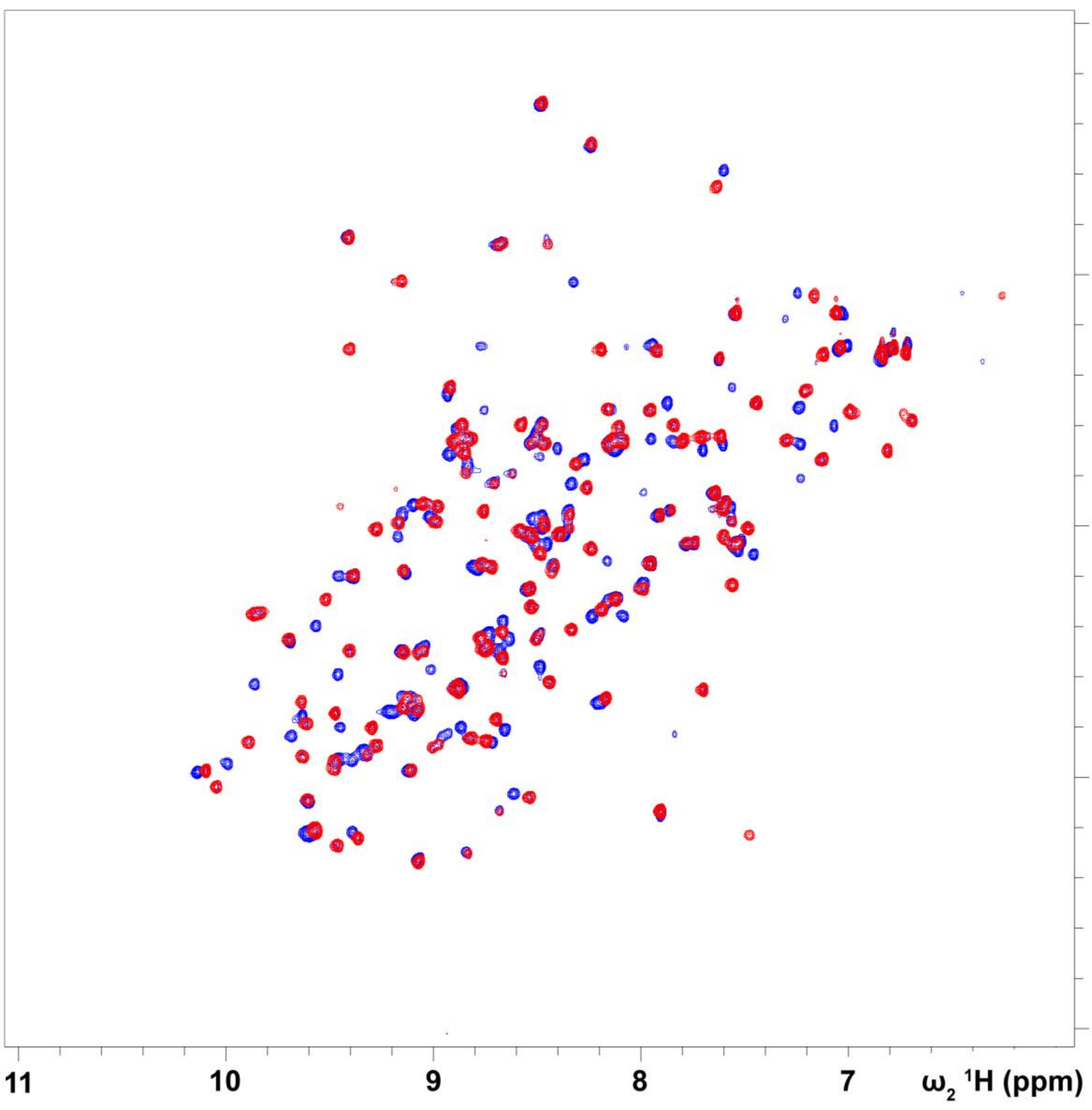

Figure S11. 2D ^{1}H-^{15}N-HSQC spectra of human apo-FABP3 in 20 mM potassium phosphate buffer pH 7.6 with 50 mM KCl. Apo-FABP3 is shown in blue and the FABP3-C16:0-CoA complex – in red.

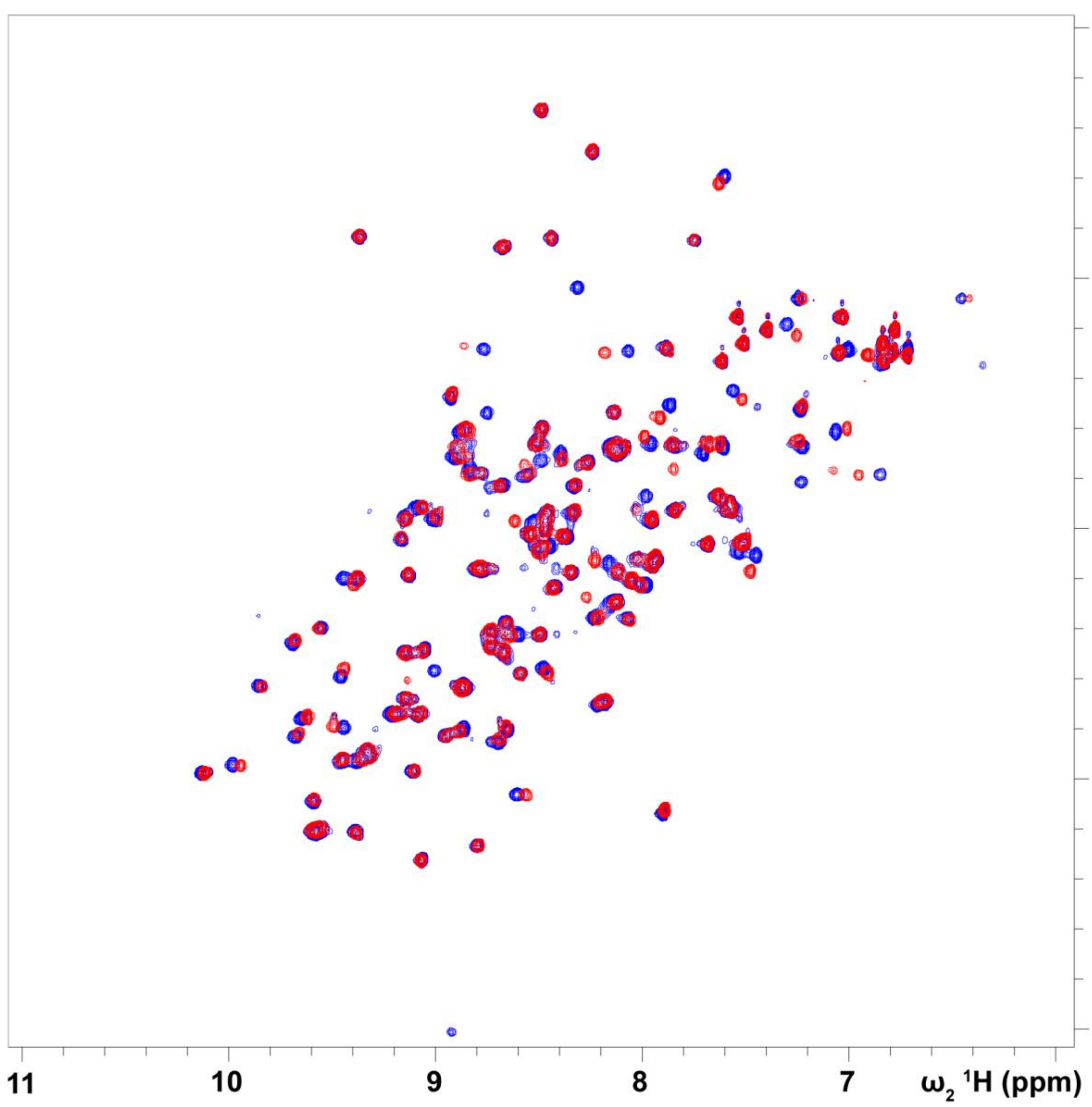

Figure S12. 2D ^{1}H-^{15}N-HSQC spectra of human apo-FABP3 in 20 mM potassium phosphate buffer pH 7.6 with 50 mM KCl. Apo-FABP3 is shown in blue and the FABP3-C14:0-carnitine complex – in red.

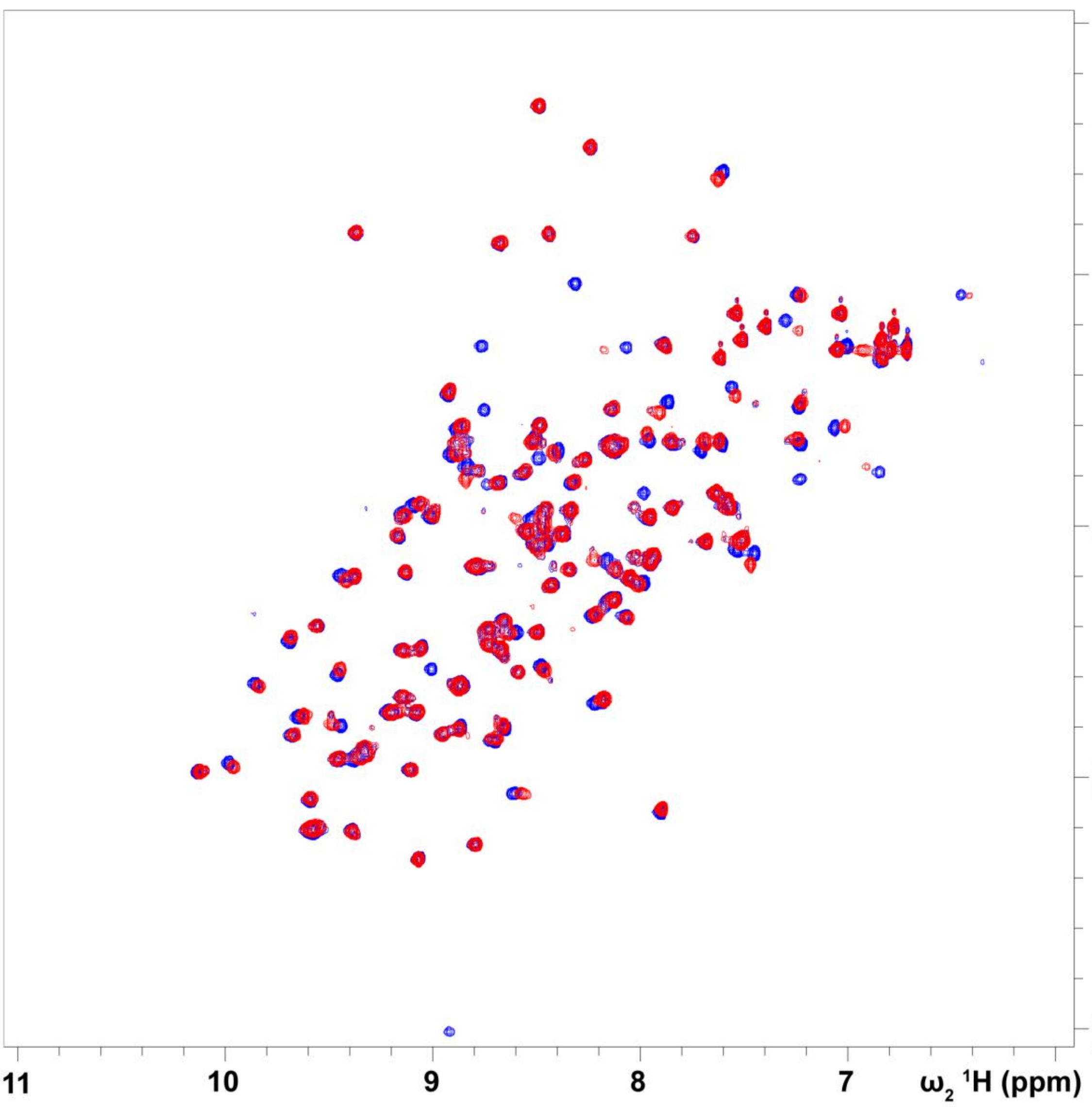

Figure S13. 2D ^{1}H-^{15}N-HSQC spectra of human apo-FABP3 in 20 mM potassium phosphate buffer pH 7.6 with 50 mM KCl. Apo-FABP3 is shown in blue and the FABP3-C18:1(n-9)*c*-carnitine complex – in red.

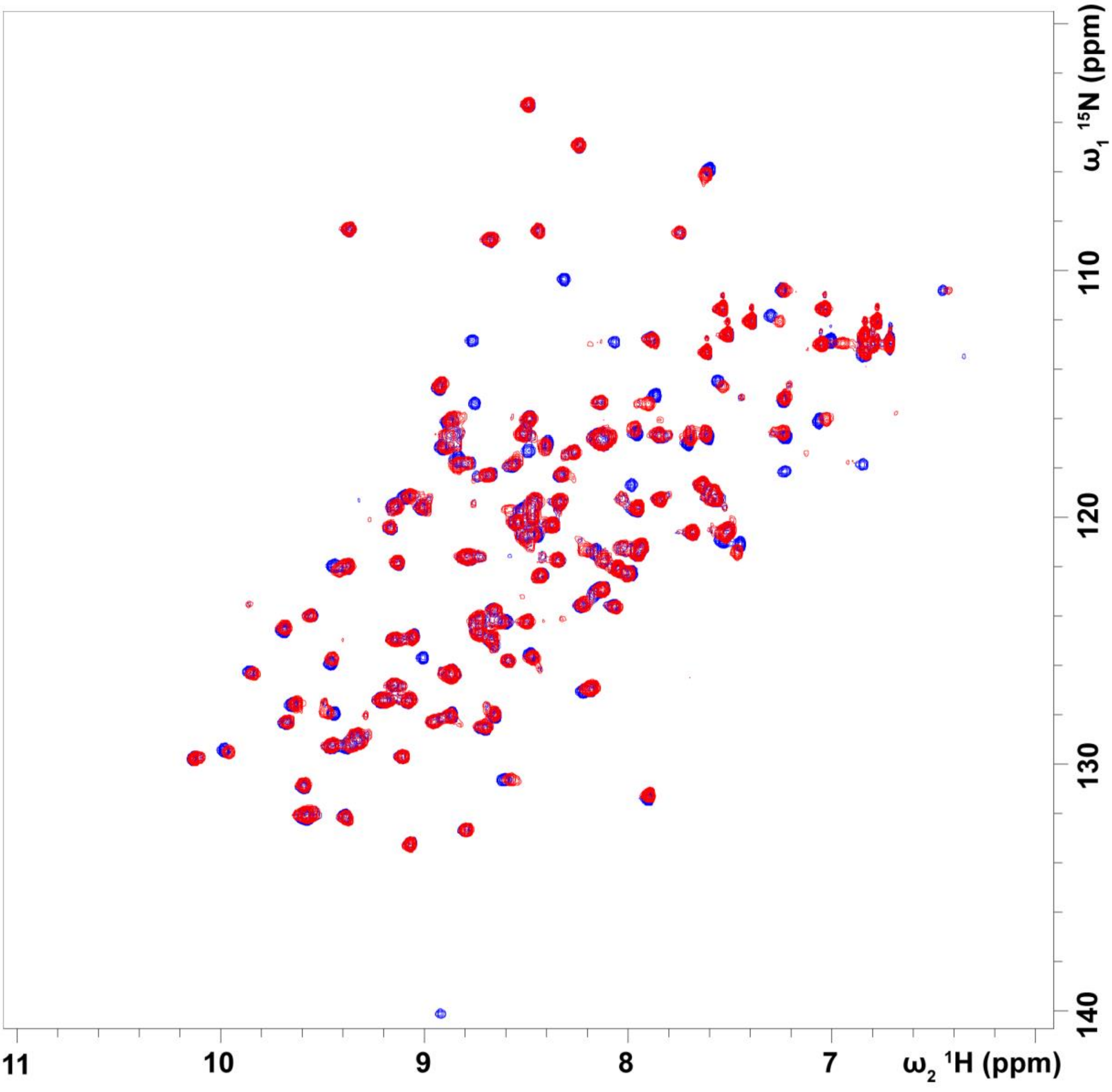

Figure S14. 2D ^{1}H-^{15}N-HSQC spectra of human apo-FABP3 in 20 mM potassium phosphate buffer pH 7.6 with 50 mM KCl. Apo-FABP3 is shown in blue and the FABP3-C18:1(n-9)*t*-carnitine complex – in red.

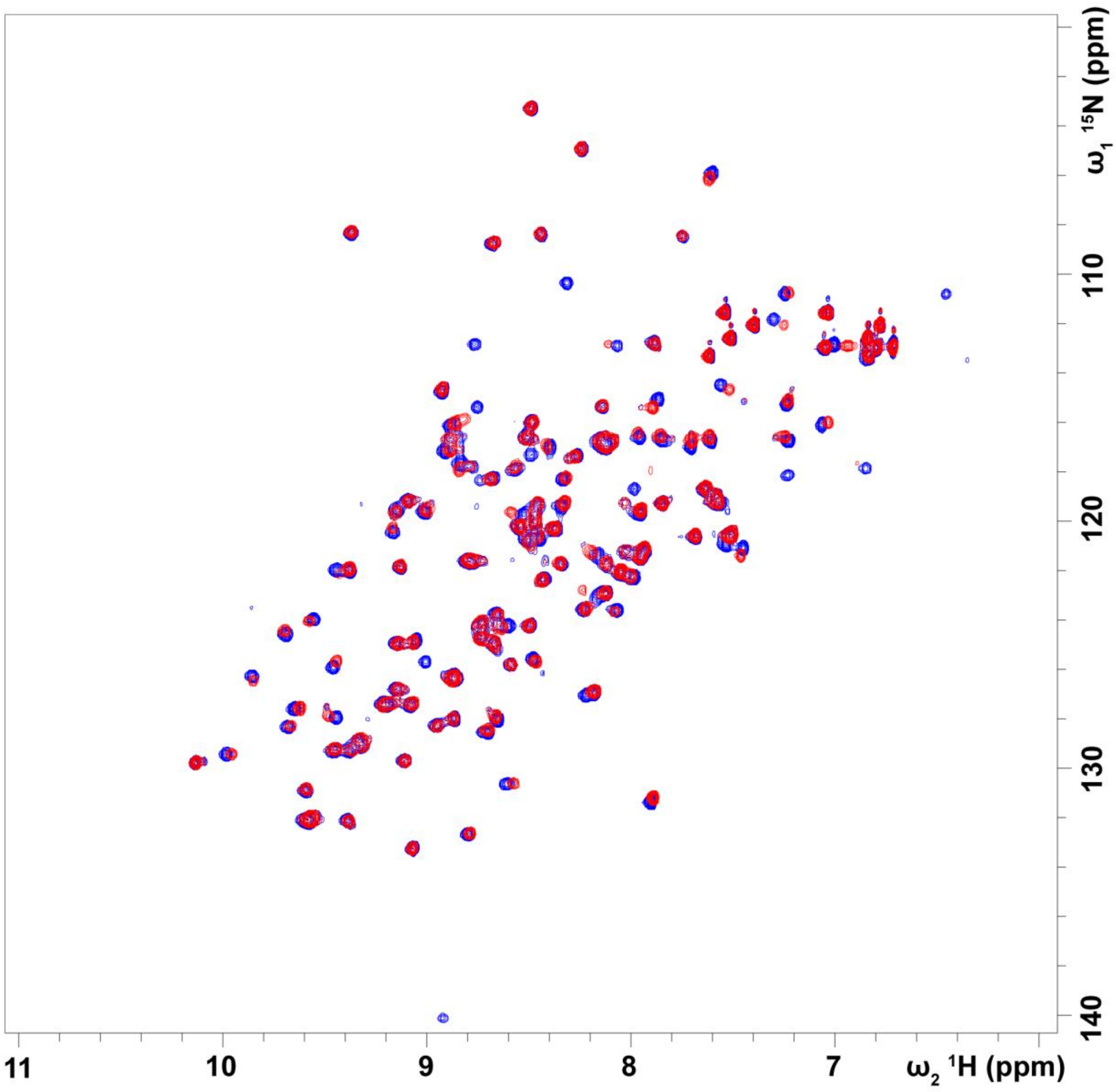

Figure S15. 2D ^{1}H-^{15}N-HSQC spectra of human apo-FABP3 in 20 mM potassium phosphate buffer pH 7.6 with 50 mM KCl. Apo-FABP3 is shown in blue and the FABP3-C20:5(n-3)*c*-carnitine complex – in red.

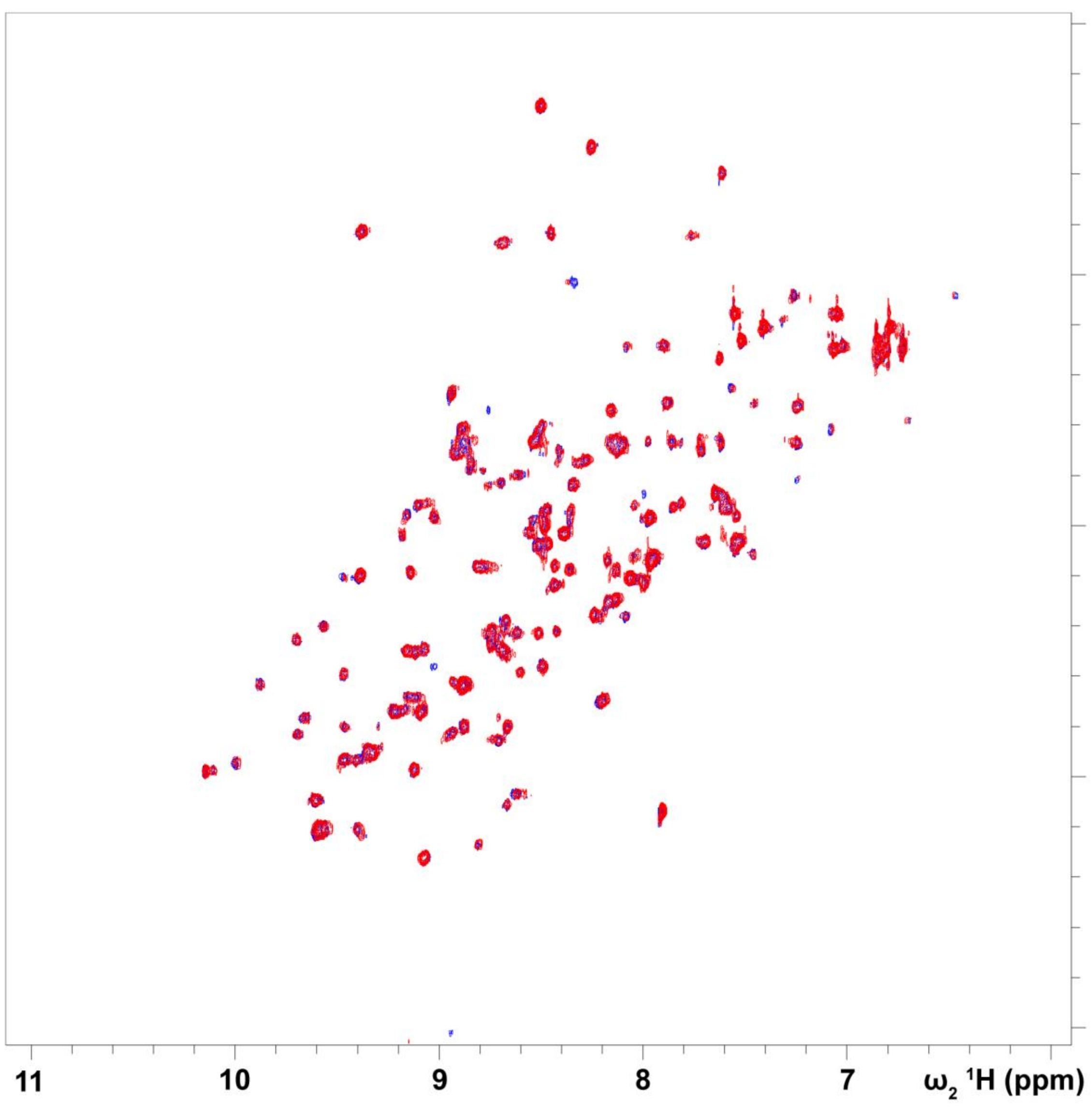

Figure S16. 2D ^{1}H-^{15}N-HSQC spectra of human apo-FABP3 in 20 mM potassium phosphate buffer pH 7.6 with 50 mM KCl. Apo-FABP3 is shown in blue and the FABP3-C8:0-carnitine complex – in red.

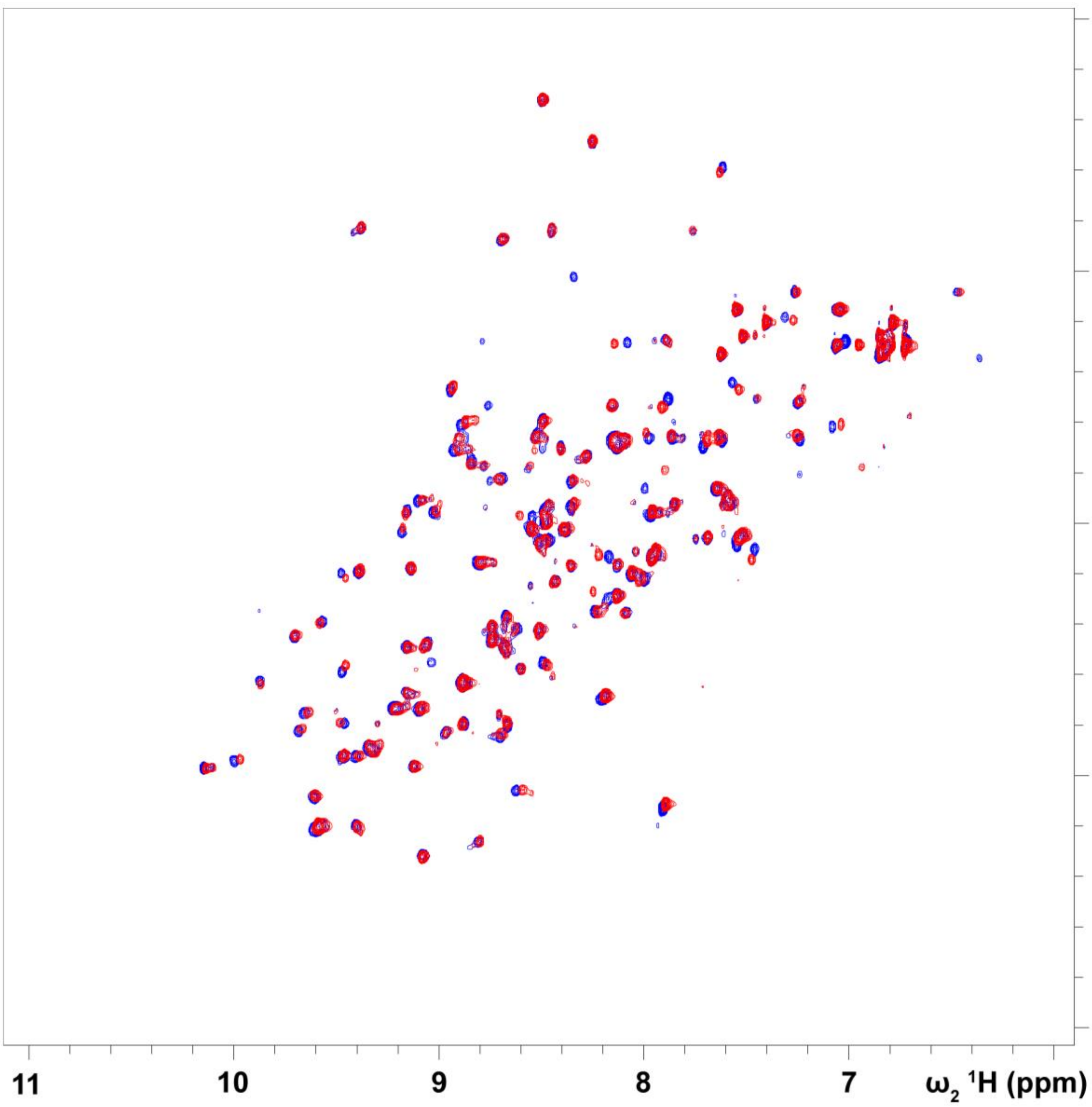

Figure S17. 2D ^{1}H-^{15}N-HSQC spectra of human apo-FABP3 in 20 mM potassium phosphate buffer pH 7.6 with 50 mM KCl. Apo-FABP3 is shown in blue and the FABP3-C12:0-carnitine complex – in red.

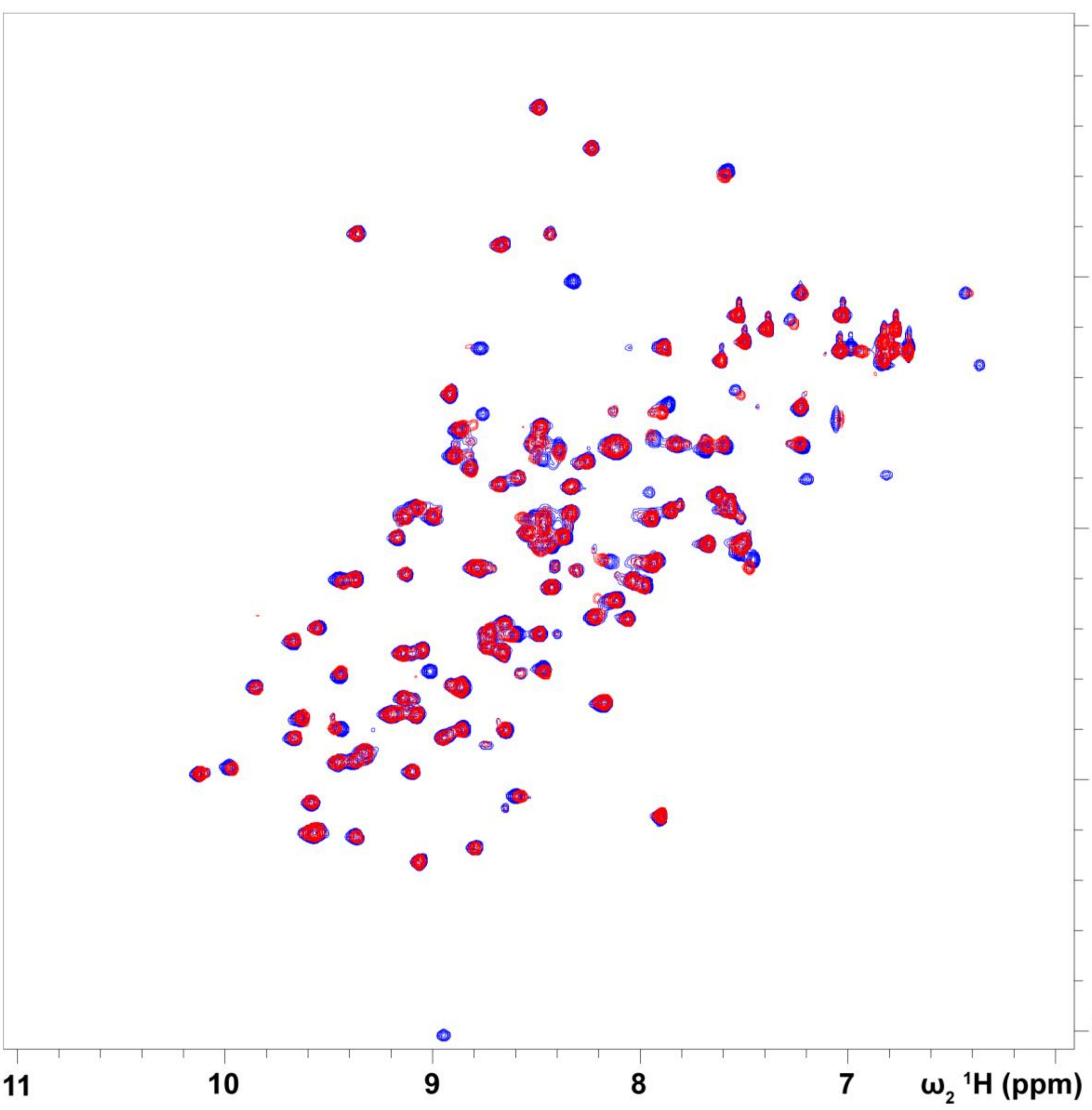

Figure S18. 2D ^{1}H-^{15}N-HSQC spectra of human apo-FABP3 in 20 mM potassium phosphate buffer pH 7.6 with 50 mM KCl. Apo-FABP3 is shown in blue and the FABP3-C16:0-carnitine complex – in red.

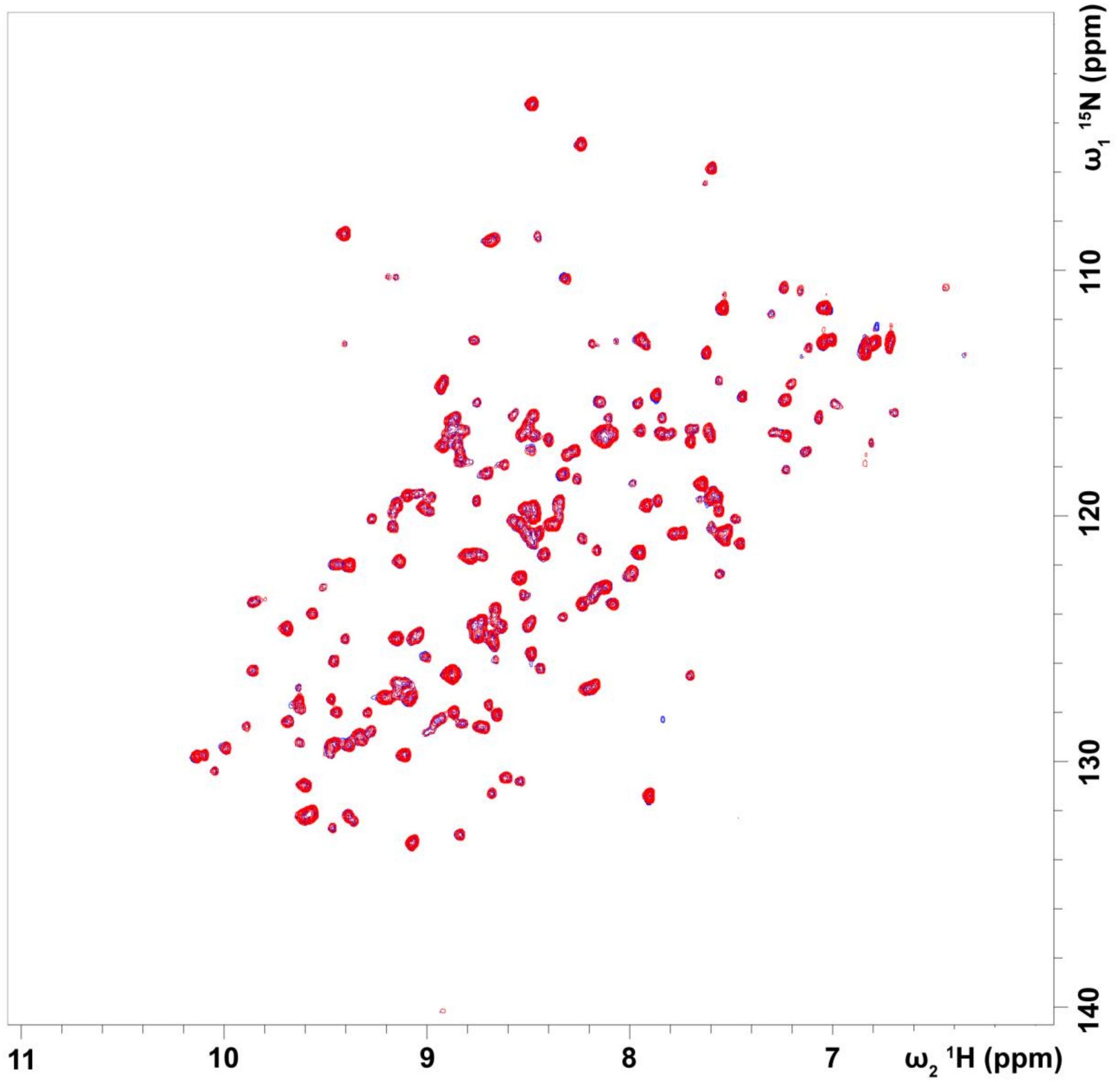

Figure S19. 2D ^{1}H-^{15}N-HSQC spectra of human apo-FABP3 in 20 mM potassium phosphate buffer pH 7.6 with 50 mM KCl. Apo-FABP3 is shown in blue and the FABP3-carnitine complex – in red.

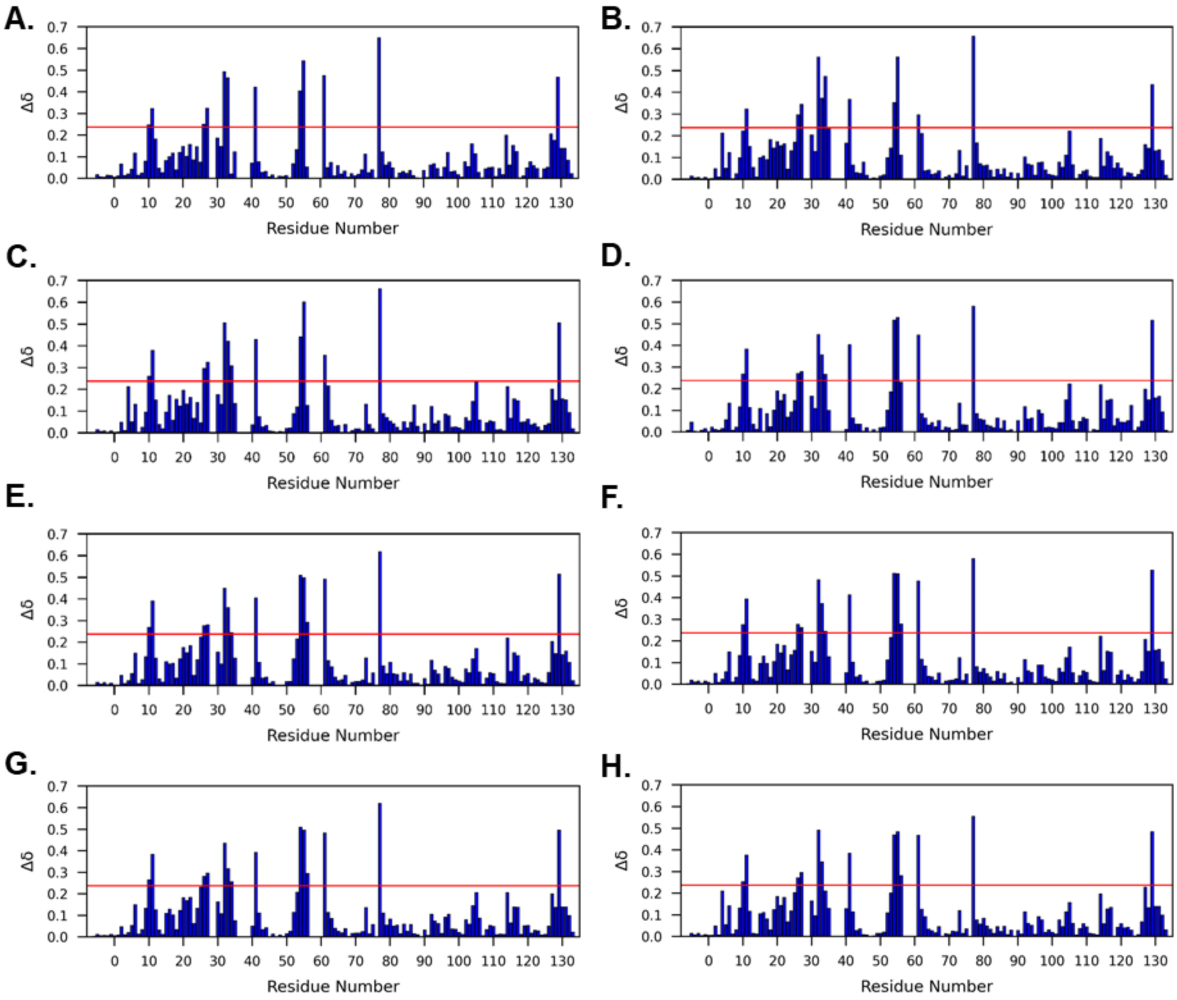

Figure S20. Graphical representation of averaged backbone amide ^{1}H and ^{15}N CSP between apo-FABP3 and FABP3-ligand complexes: (**A**) C8:0; (**B**) C12:0; (**C**) C14:0; (**D**) C16:0; (**E**) C18:1(n-9)*c*; (**F**) C18:1(n-9)*t*; (**G**) C18:0; and (**H**) C20:5(n-3)*c* in 20 mM potassium phosphate buffer pH 7.6 with 50 mM KCl. The red line corresponds to the mean value plus one standard deviation. Residues with CSPs values above the red line are considered as the ones with significant chemical shift differences.

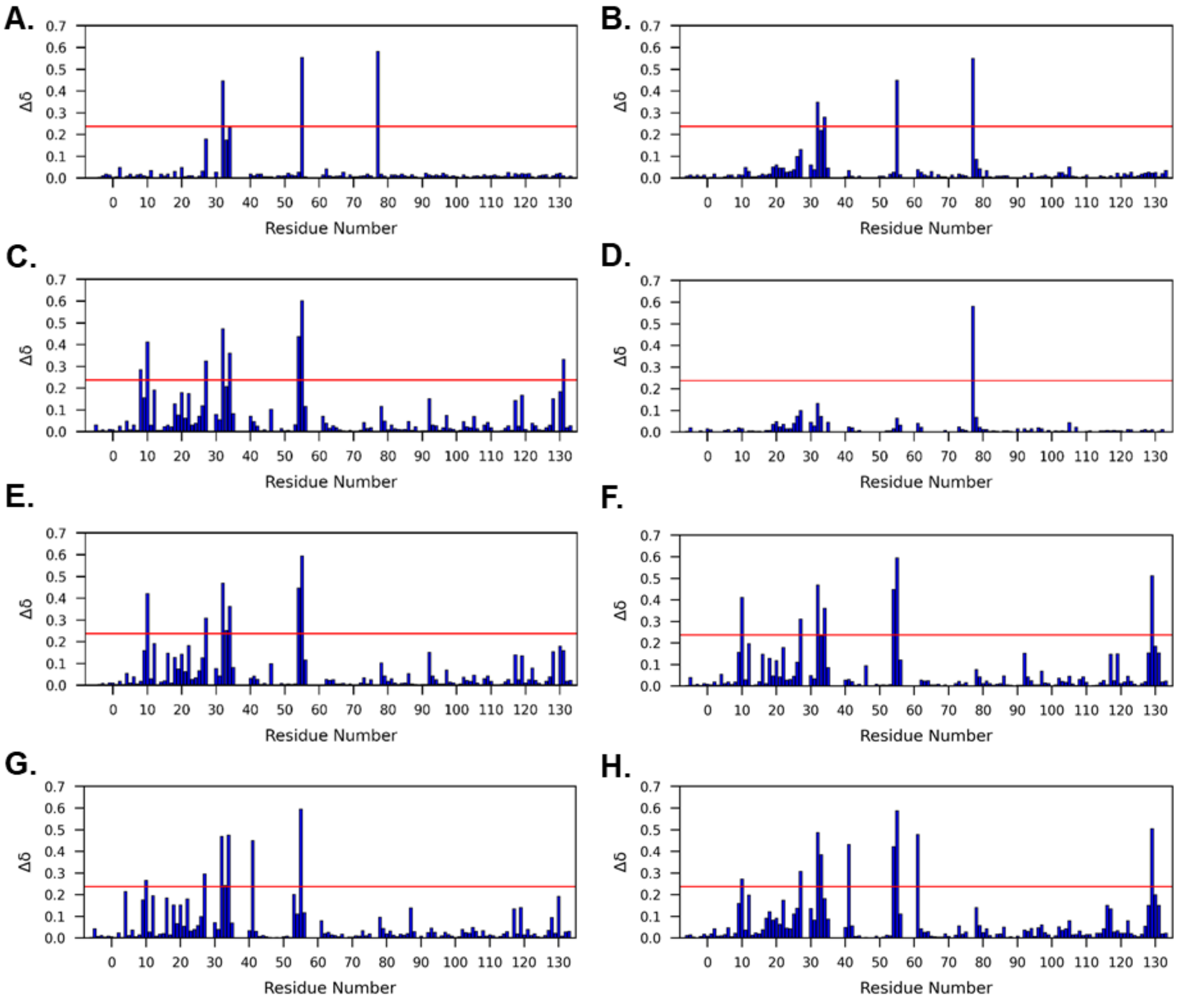

Figure S21. Graphical representation of averaged backbone amide ^{1}H and ^{15}N CSP between apo-FABP3 and FABP3-ligand complexes: (**A**) C8:0-carnitine; (**B**) C12:0-carnitine; (**C**) C14:0-carnitine; (**D**) C16:0-carnitine; (**E**) C18:1(n-9)*c*-carnitine; (**F**) C18:1(n-9)*t*-carnitine; (**G**) C20:5(n-3)*c*-carnitine, and (**H**) C16:0-CoA 20 mM potassium phosphate buffer pH 7.6 with 50 mM KCl. The red line corresponds to the mean value plus one standard deviation. Residues with CSP values above the red line are considered as the ones with significant chemical shift differences.

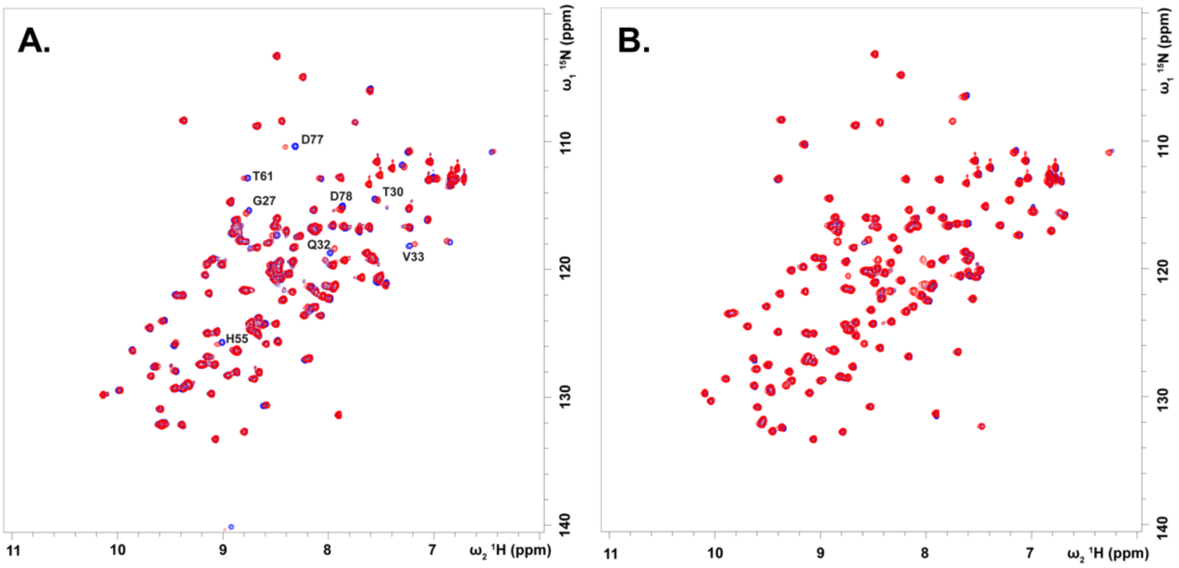

Figure S22. 2D ^{1}H-^{15}N-HSQC spectra superposition of (**A**) apo-FABP3 and (**B**) FABP3-C16:0 complex in 20 mM potassium phosphate buffer pH 7.6 and 50 mM KCl without (blue) and with (red) 0.1% (v/v) Triton™ X-100 additive. Residues that were minimally affected by detergent additive were denoted as one letter symbol and residue number.

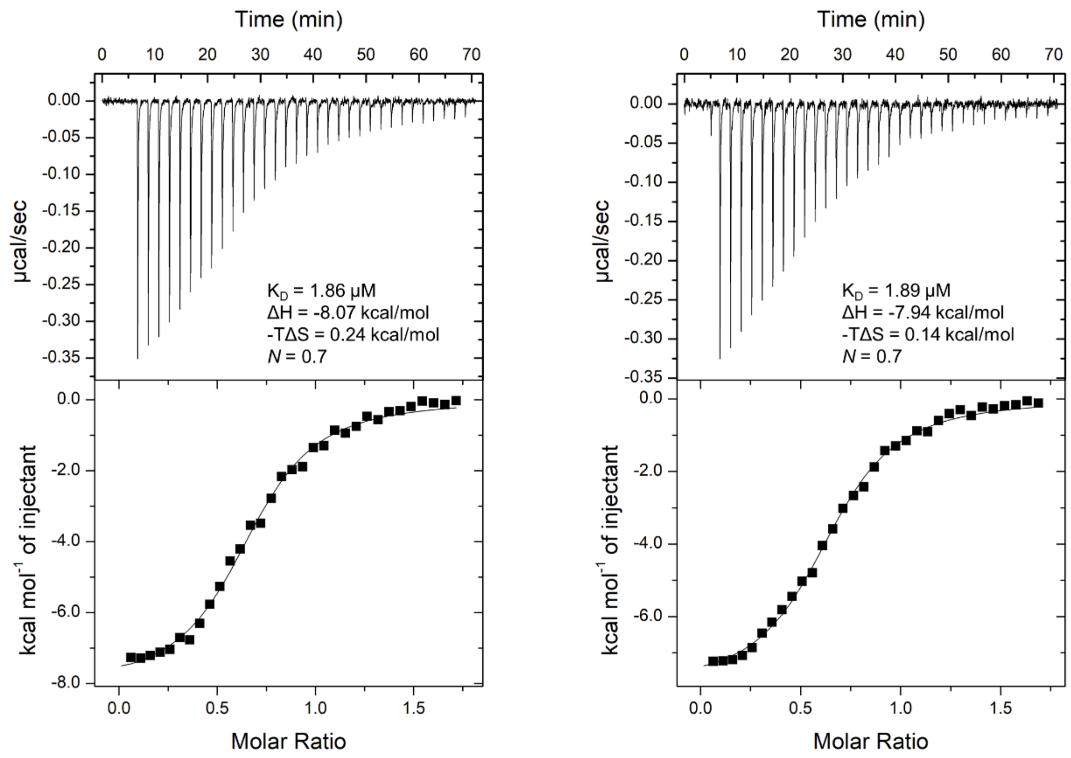

Figure S23. ITC titration data for FABP3 interaction with C8:0 at 25 °C in 20 mM potassium phosphate buffer pH 7.6 and 50 mM KCl.

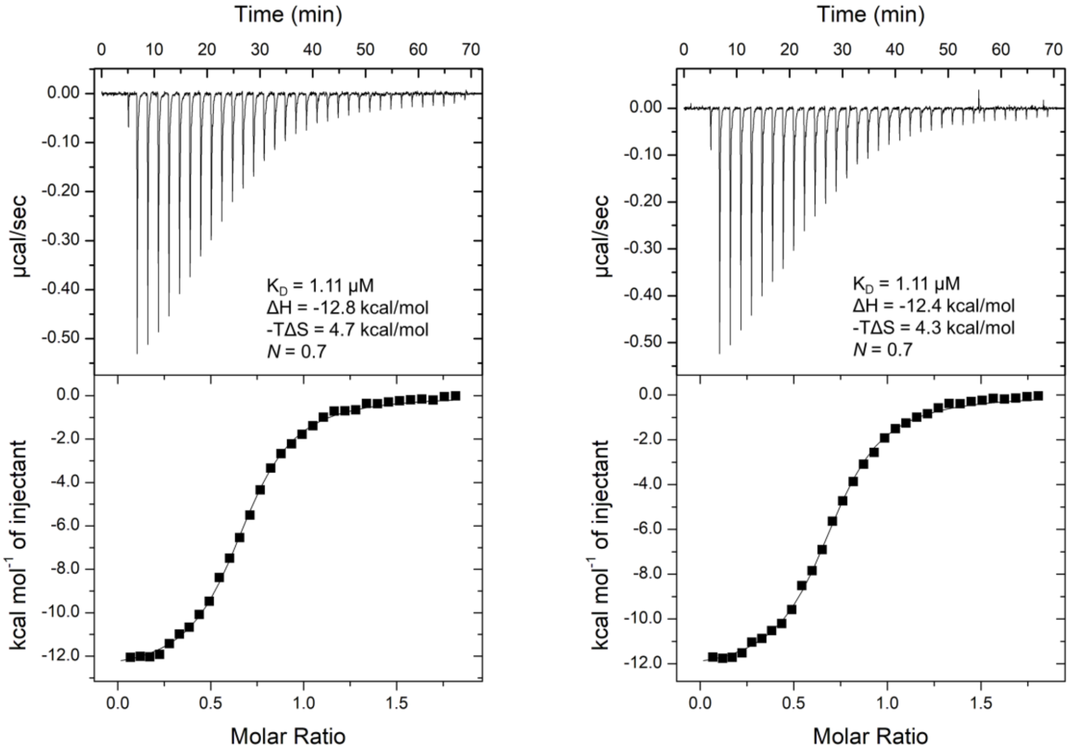

Figure S24. ITC titration data for FABP3 interaction with C8:0 at 25 °C in 20 mM potassium phosphate buffer pH 7.6 and 50 mM KCl supplemented with 0.10% (v/v) Triton™ X-100.

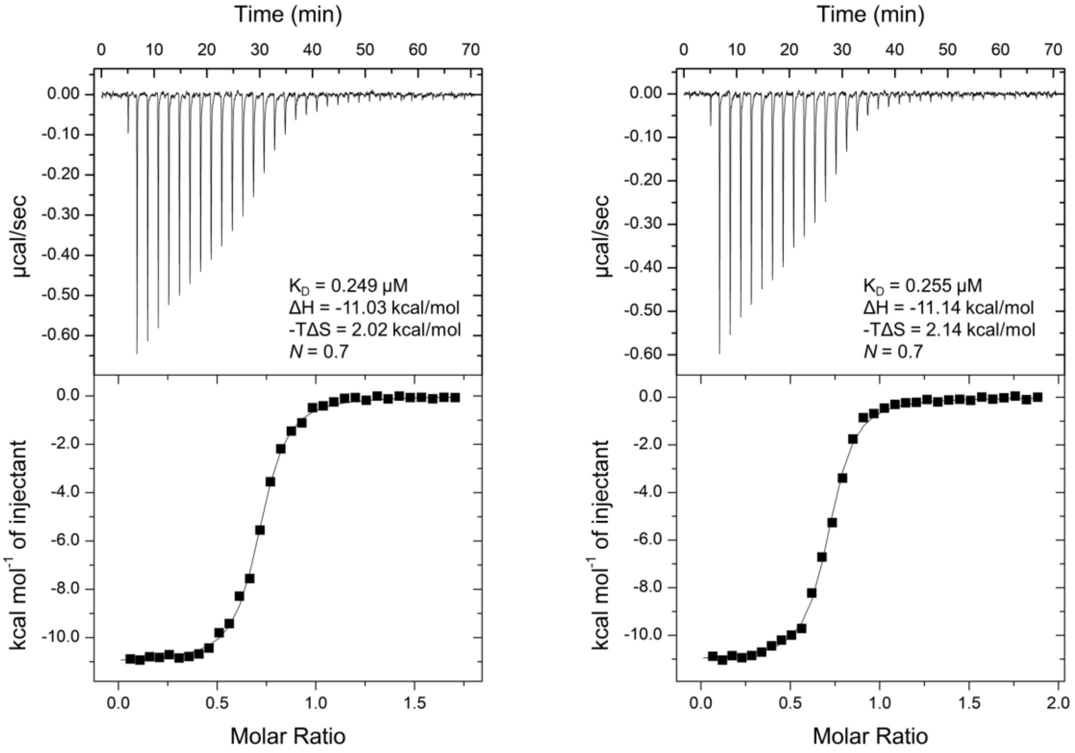

Figure S25. ITC titration data for FABP3 interaction with C10:0 at 25 °C in 20 mM potassium phosphate buffer pH 7.6 and 50 mM KCl.

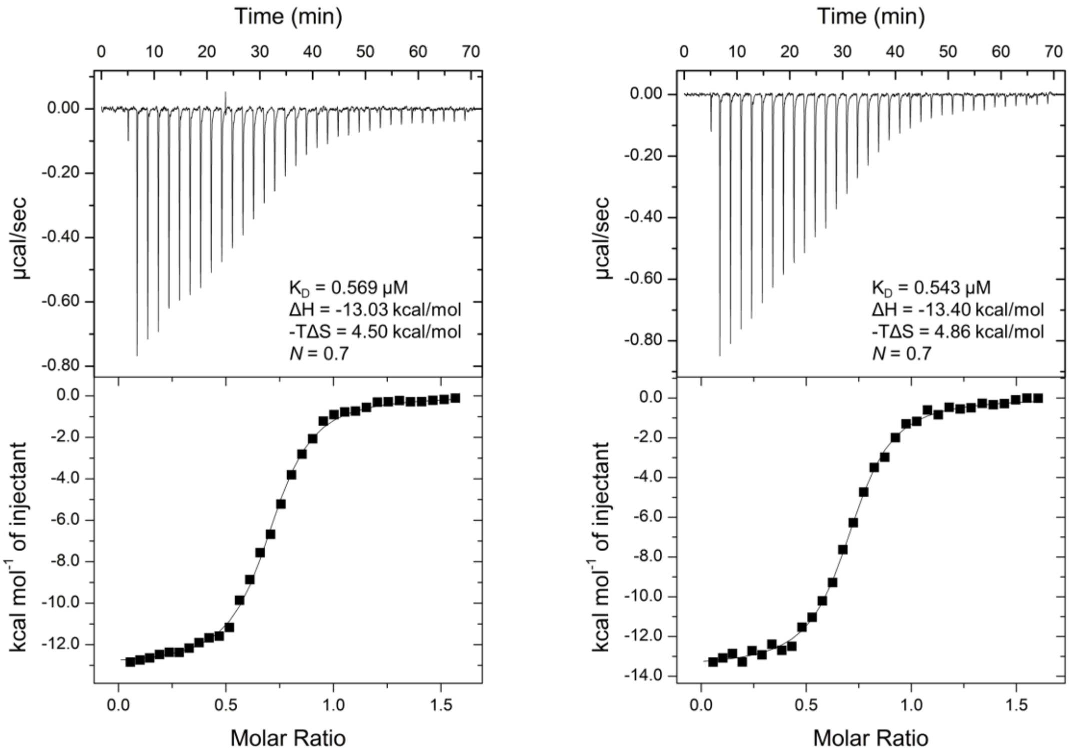

Figure S26. ITC titration data for FABP3 interaction with C10:0 at 25 °C in 20 mM potassium phosphate buffer pH 7.6 and 50 mM KCl supplemented with 0.10% (v/v) Triton™ X-100.

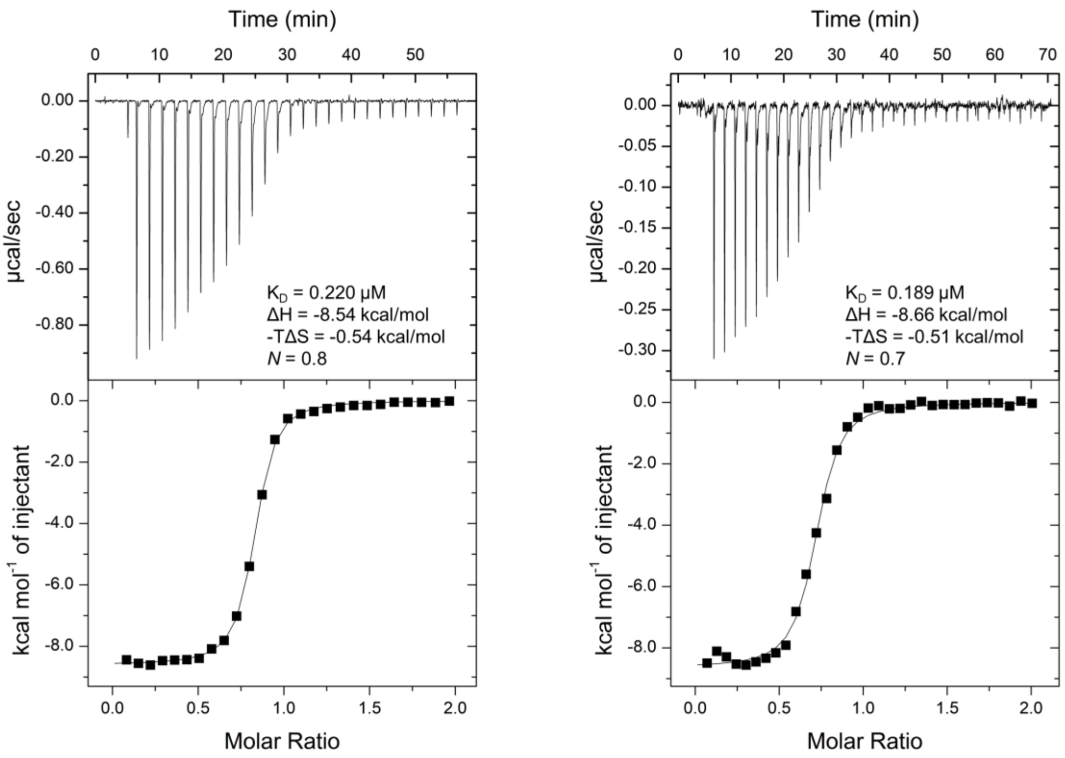

Figure S27. ITC titration data for FABP3 interaction with C12:0 at 25 °C in 20 mM potassium phosphate buffer pH 7.6 and 50 mM KCl.

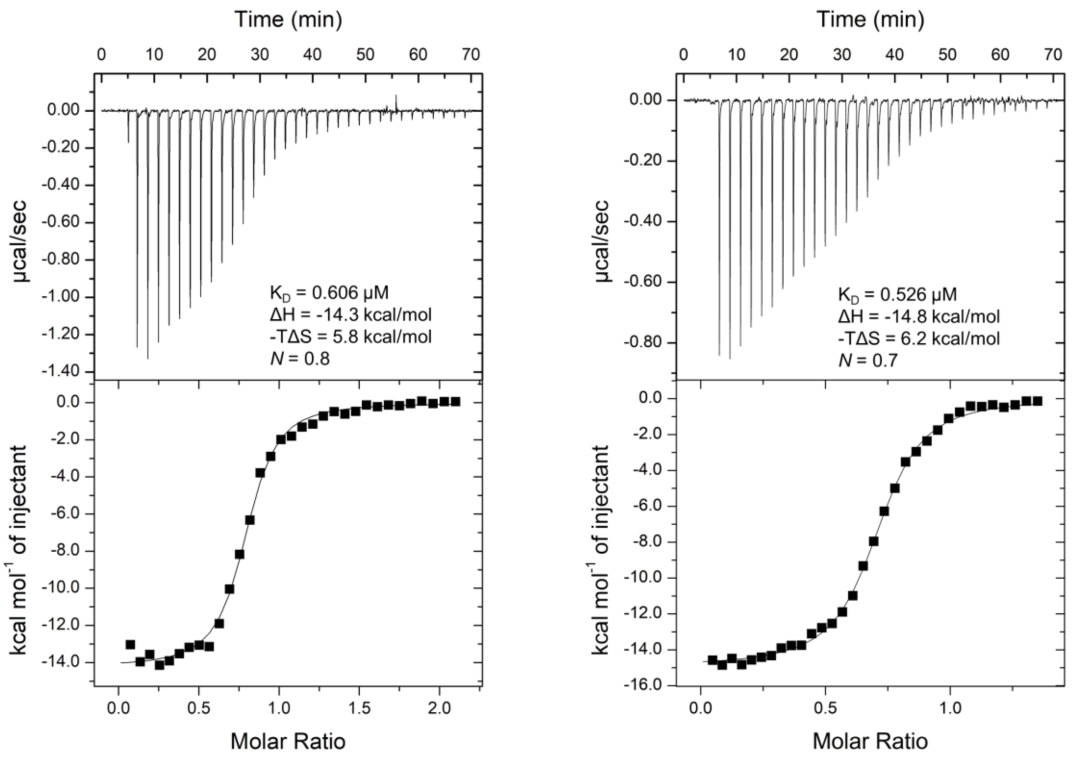

Figure S28. ITC titration data for FABP3 interaction with C12:0 at 25 °C in 20 mM potassium phosphate buffer pH 7.6 and 50 mM KCl supplemented with 0.10% (v/v) Triton™ X-100.

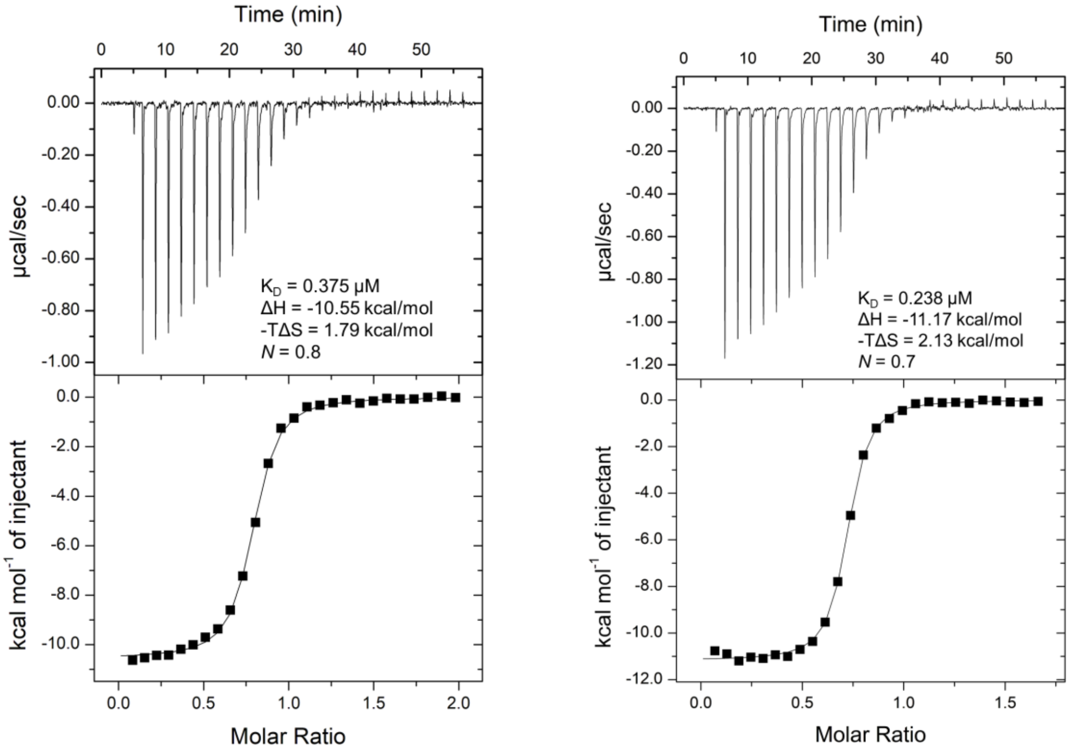

Figure S29. ITC titration data for FABP3 interaction with C14:0 at 25 °C in 20 mM potassium phosphate buffer pH 7.6 and 50 mM KCl supplemented with 0.10% (v/v) Triton™ X-100.

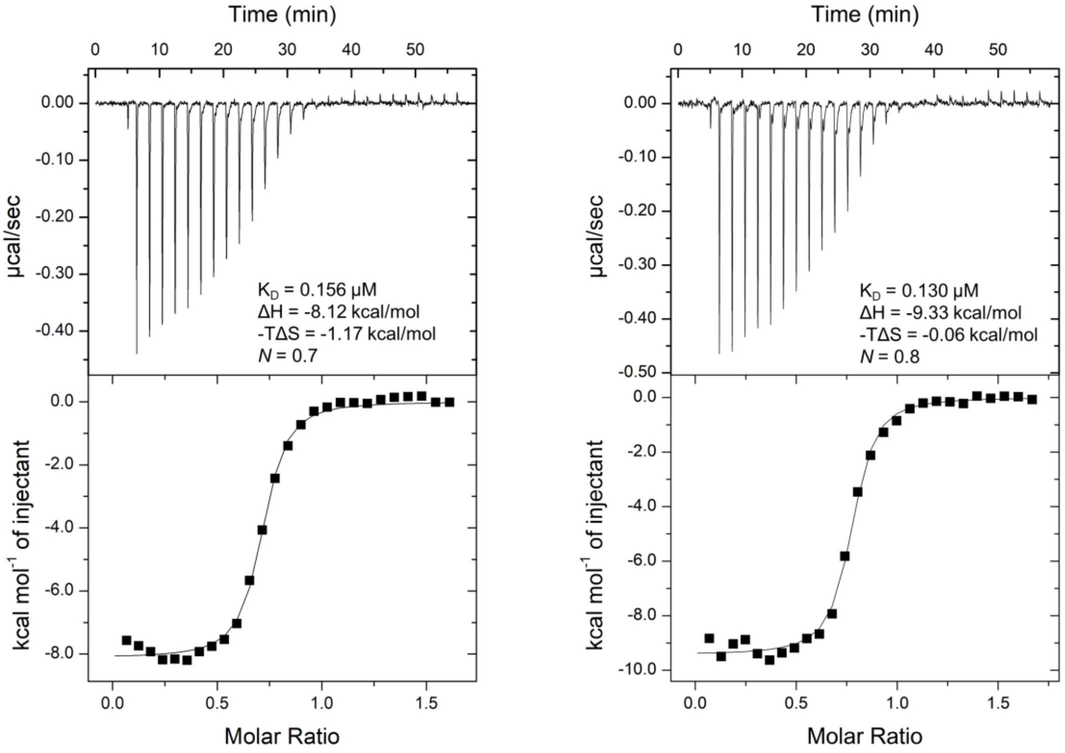

Figure S30. ITC titration data for FABP3 interaction with C16:0 at 25 °C in 20 mM potassium phosphate buffer pH 7.6 and 50 mM KCl supplemented with 0.10% (v/v) Triton™ X-100.

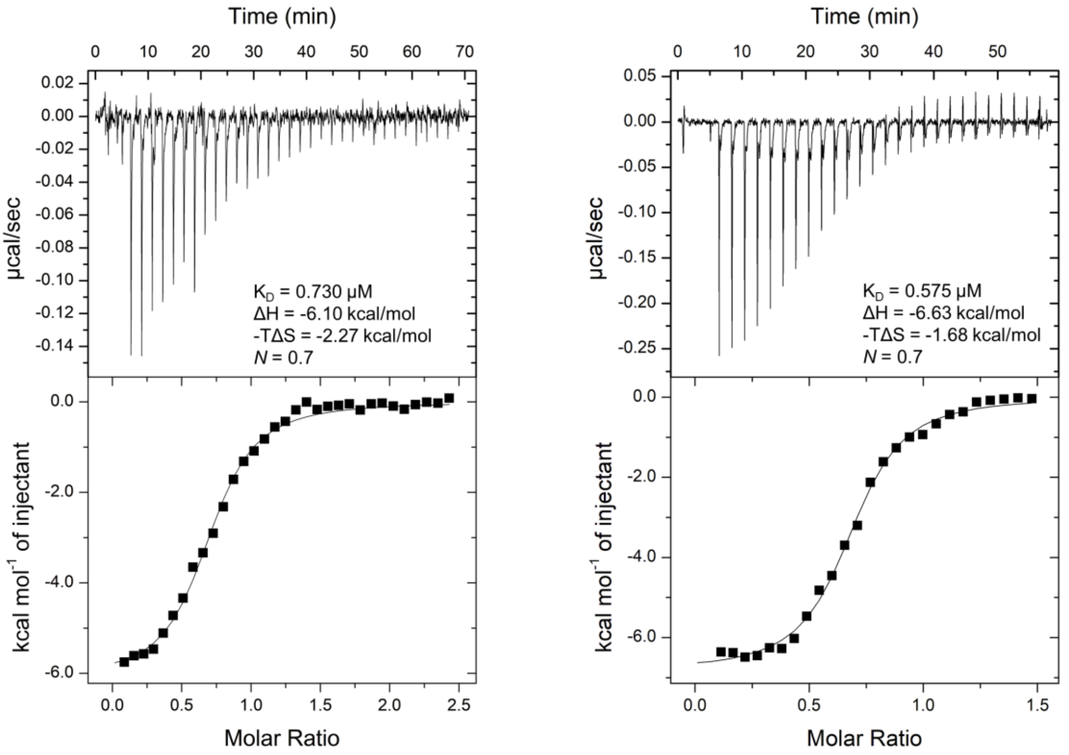

Figure S31. ITC titration data for FABP3 interaction with C18:0 at 25 °C in 20 mM potassium phosphate buffer pH 7.6 and 50 mM KCl supplemented with 0.10% (v/v) Triton™ X-100.

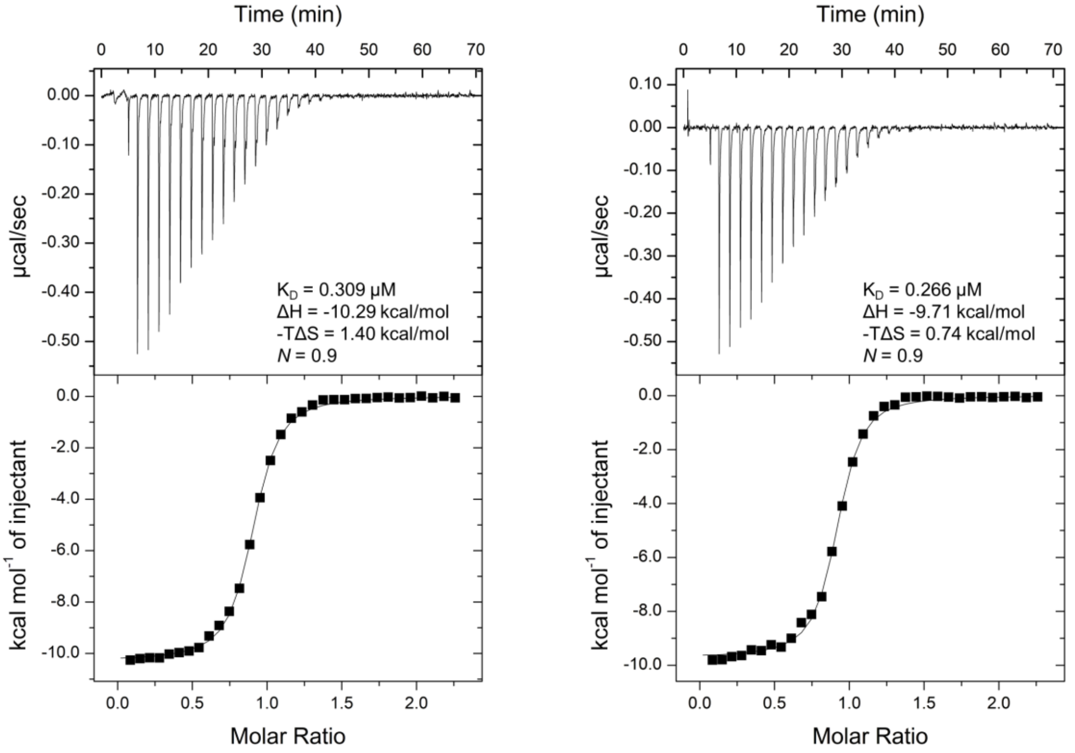

Figure S32. ITC titration data for FABP3 interaction with C18:1(n-9)*c* at 25 °C in 20 mM potassium phosphate buffer pH 7.6 and 50 mM KCl supplemented with 0.10% (v/v) Triton™ X-100.

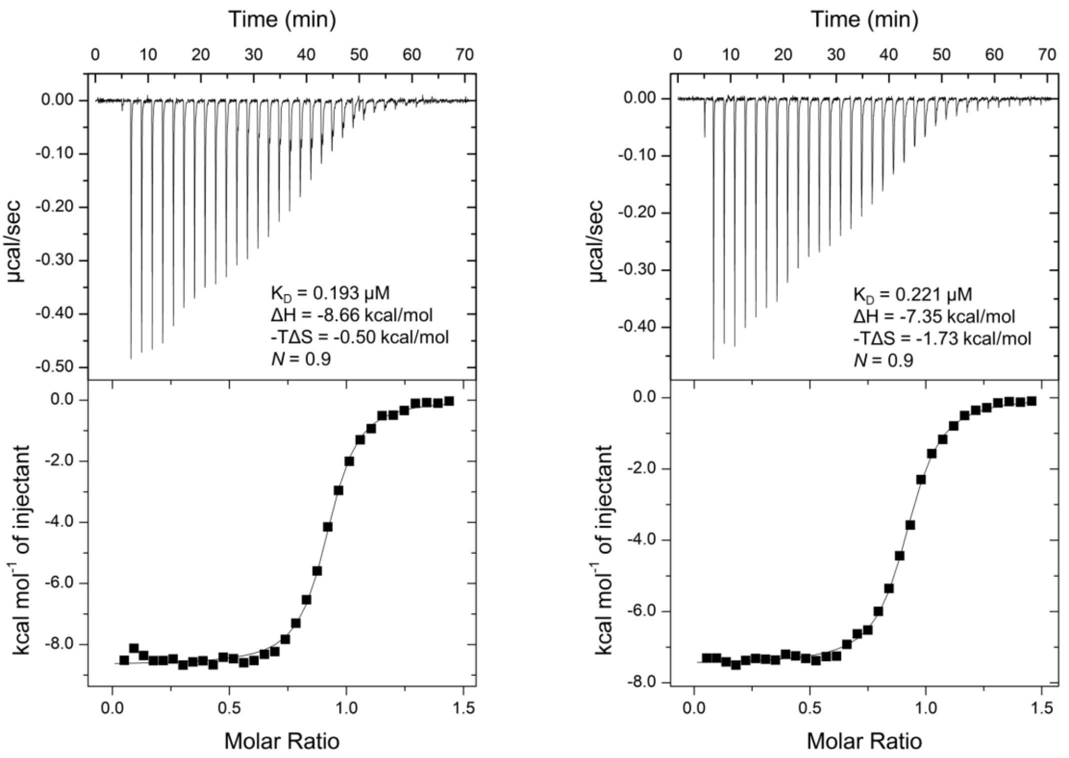

Figure S33. ITC titration data for FABP3 interaction with C18:1(n-9)*t* at 25 °C in 20 mM potassium phosphate buffer pH 7.6 and 50 mM KCl supplemented with 0.10% (v/v) Triton™ X-100.

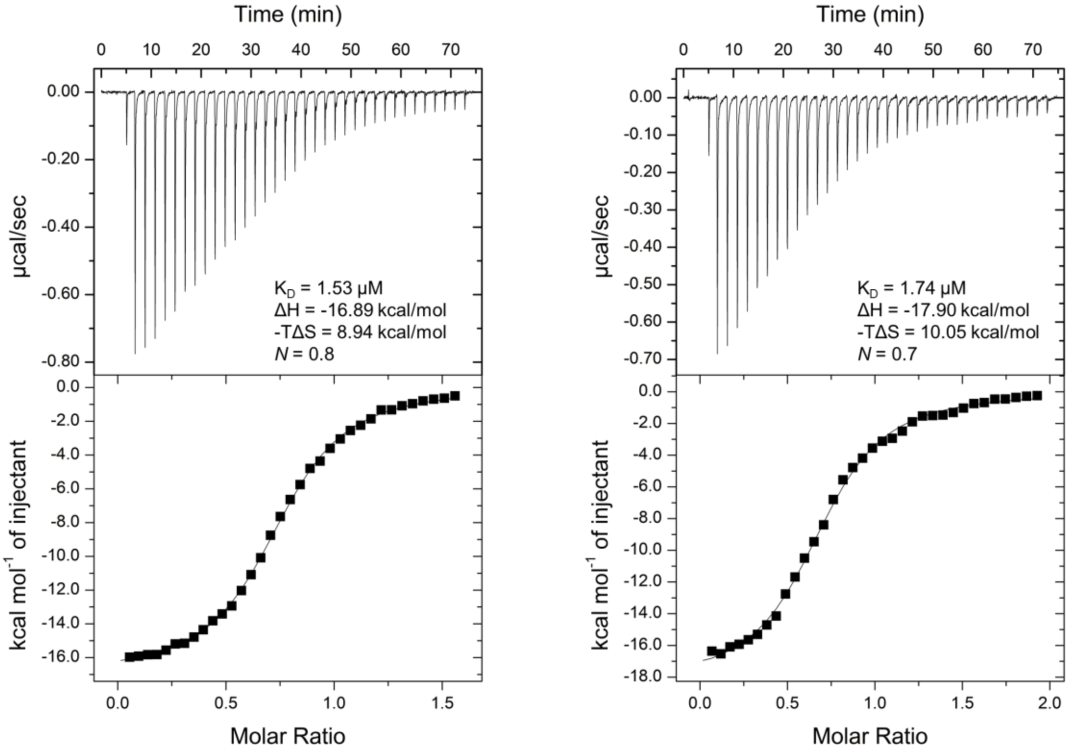

Figure S34. ITC titration data for FABP3 interaction with C20:5(n-3)*c* at 25 °C in 20 mM potassium phosphate buffer pH 7.6 and 50 mM KCl supplemented with 0.10% (v/v) Triton™ X-100.

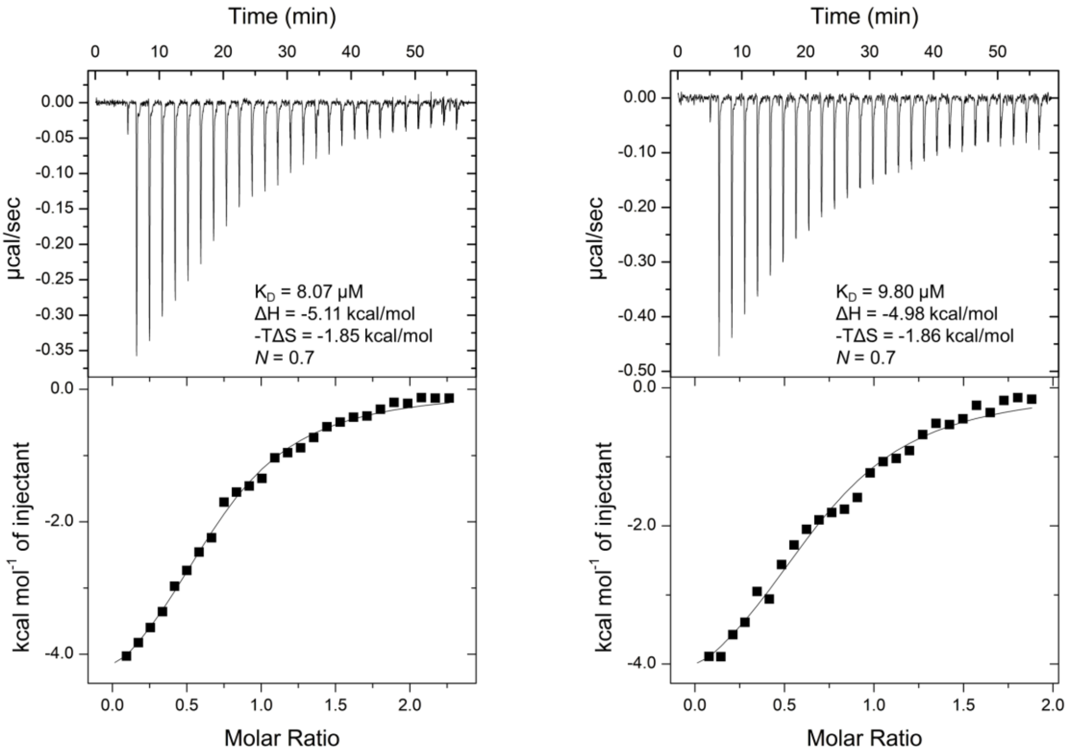

Figure S35. ITC titration data for FABP3 interaction with C16:0-CoA at 25 °C in 20 mM potassium phosphate buffer pH 7.6 and 50 mM KCl.

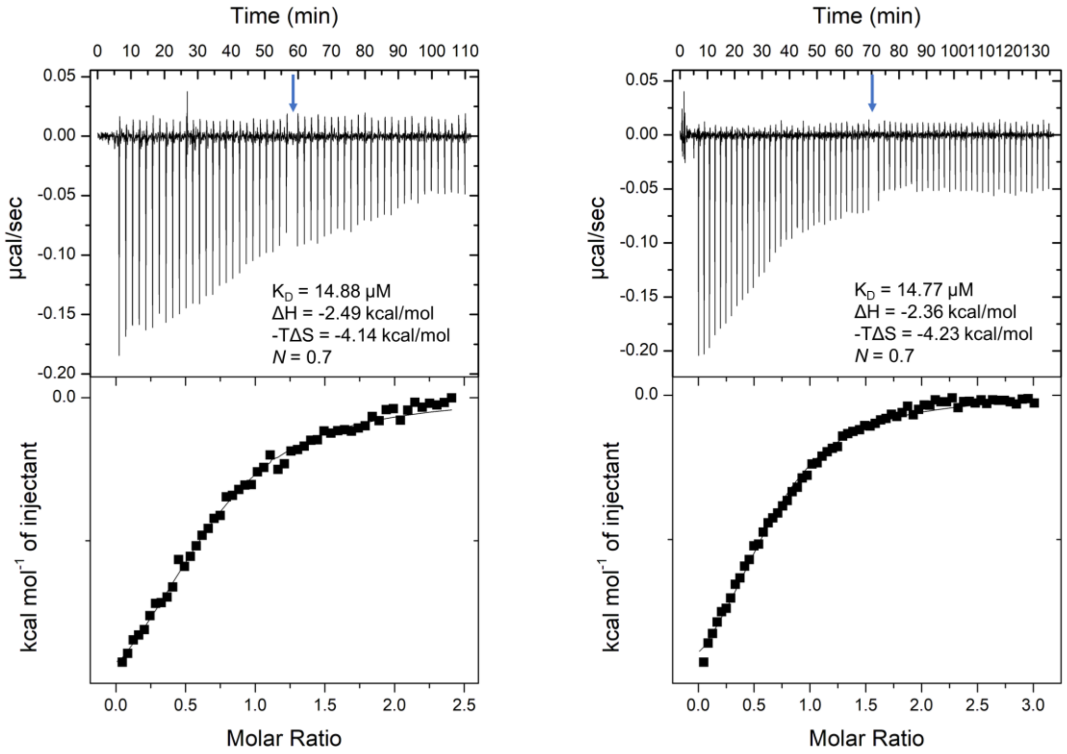

Figure S36. ITC titration data for FABP3 interaction with C20:5(n-3)*c*-carnitine at 25 °C in 20 mM potassium phosphate buffer pH 7.6 and 50 mM KCl. The blue arrow indicates the syringe refill.

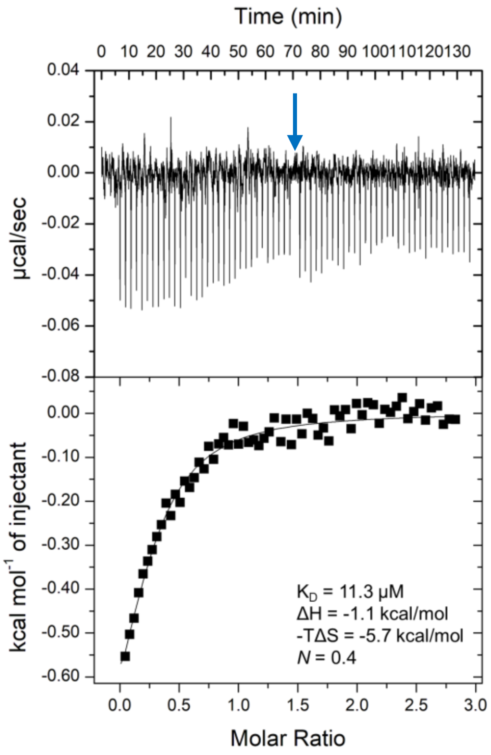

Figure S37. ITC titration data for FABP3 interaction with C14:0-carnitine at 25 °C in 20 mM potassium phosphate buffer pH 7.6 and 50 mM KCl. The blue arrow indicates the syringe refill.

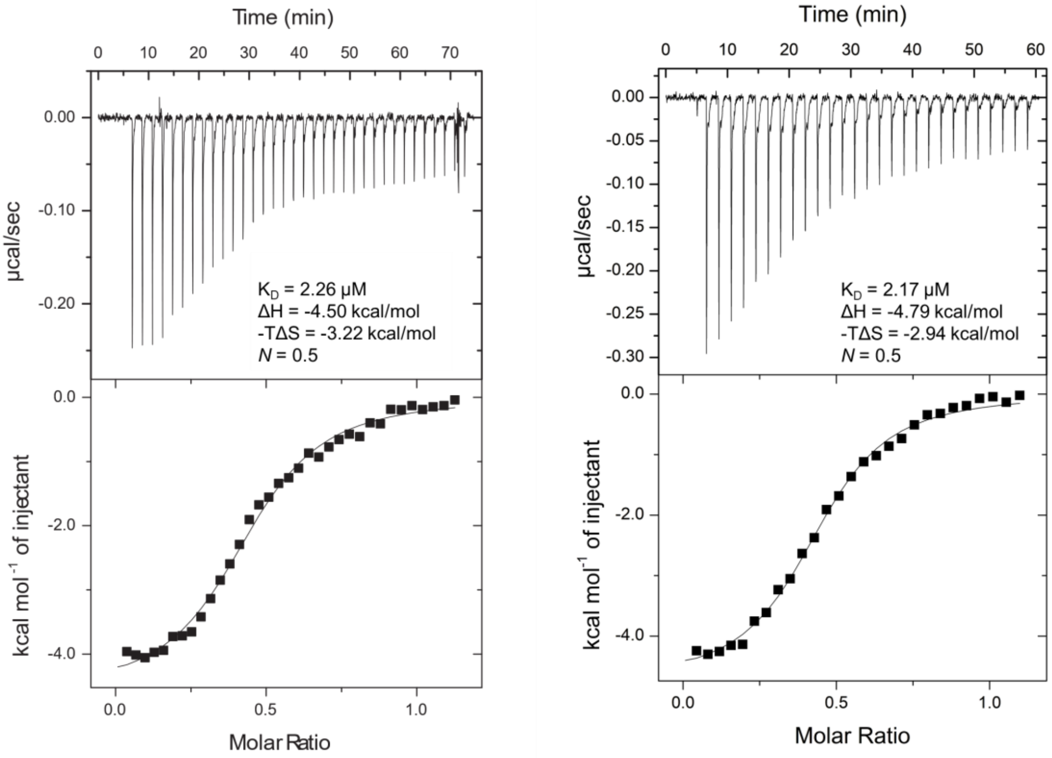

Figure S38. ITC titration data for FABP3 interaction with C18:1(n-9)*c*-carnitine at 25 °C in 20 mM potassium phosphate buffer pH 7.6 and 50 mM KCl.

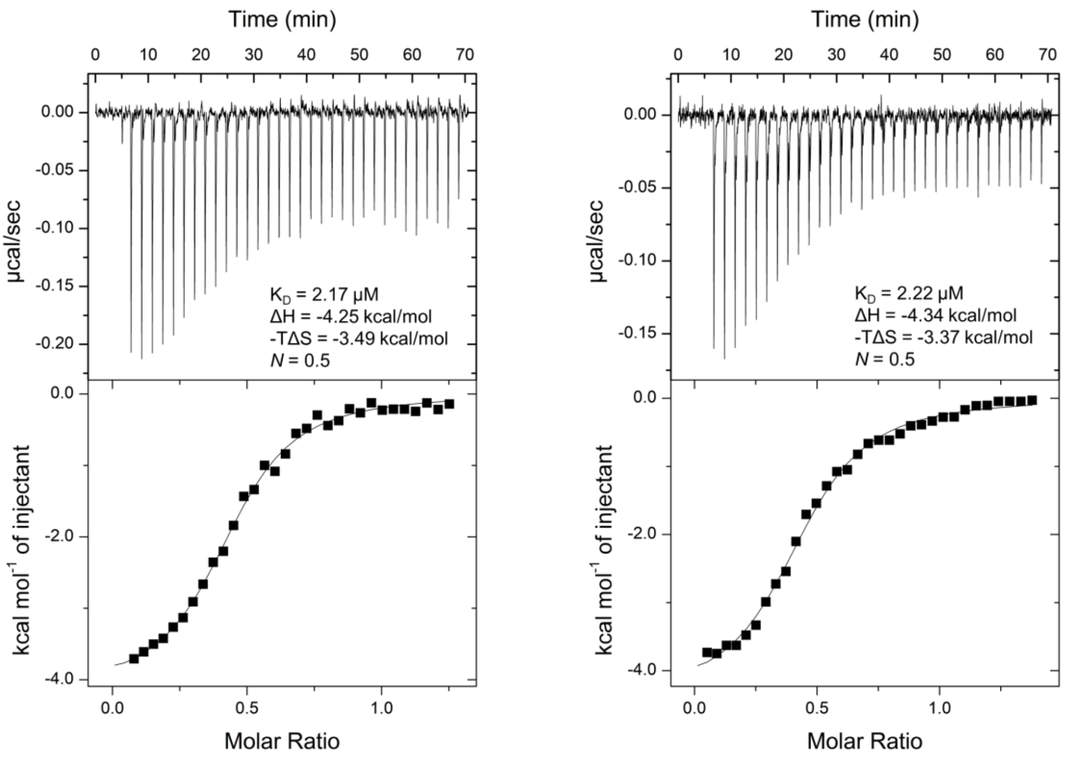

Figure S39. ITC titration data for FABP3 interaction with C18:1(n-9)*t*-carnitine at 25 °C in 20 mM potassium phosphate buffer pH 7.6 and 50 mM KCl.

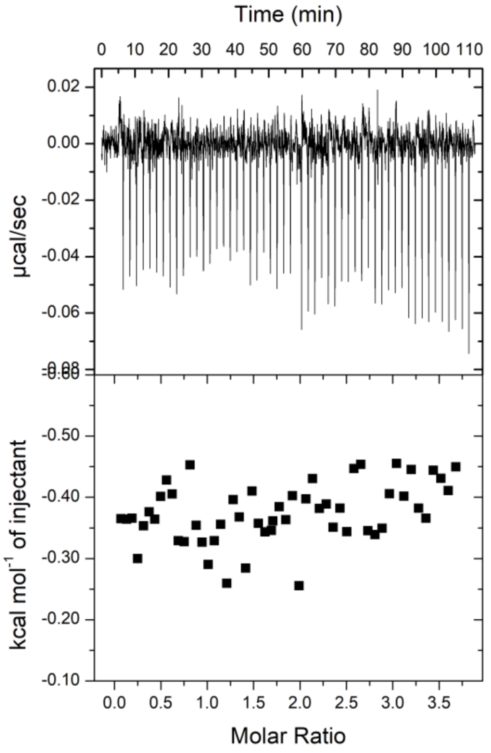

Figure S40. ITC titration data for FABP3 interaction with C16:0-carnitine at 25 °C in 20 mM potassium phosphate buffer pH 7.6 and 50 mM KCl.

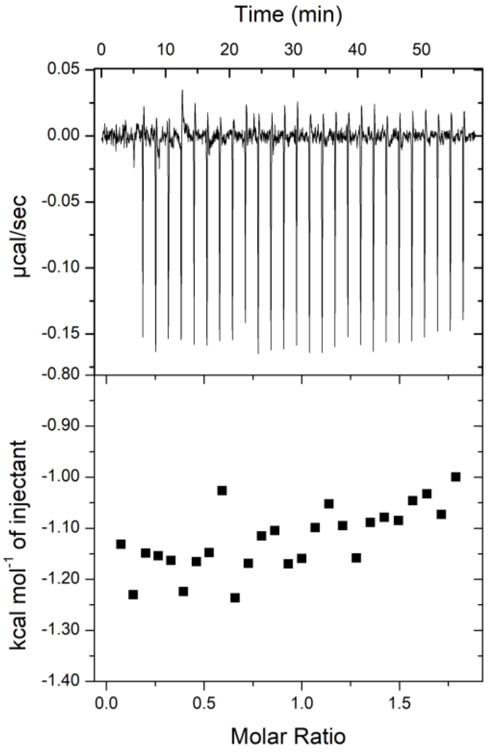

Figure S41. ITC titration data for FABP3 interaction with C8:0-carnitine at 25 °C in 20 mM potassium phosphate buffer pH 7.6 and 50 mM KCl.

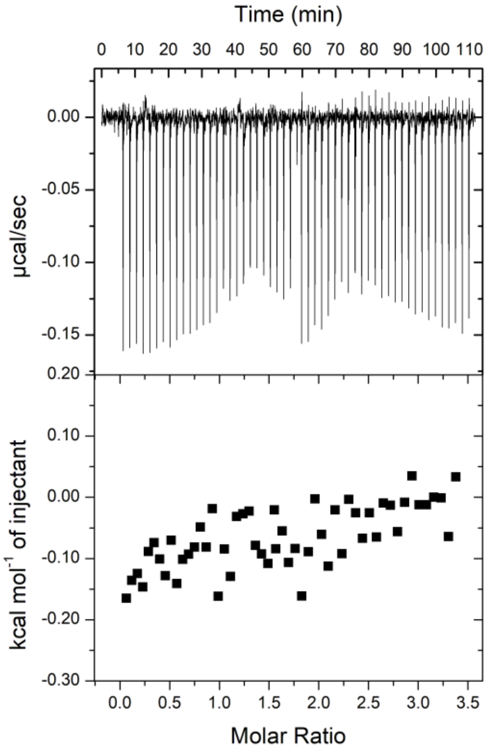

Figure S42. ITC titration data for FABP3 interaction with C12:0-carnitine at 25 °C in 20 mM potassium phosphate buffer pH 7.6 and 50 mM KCl.

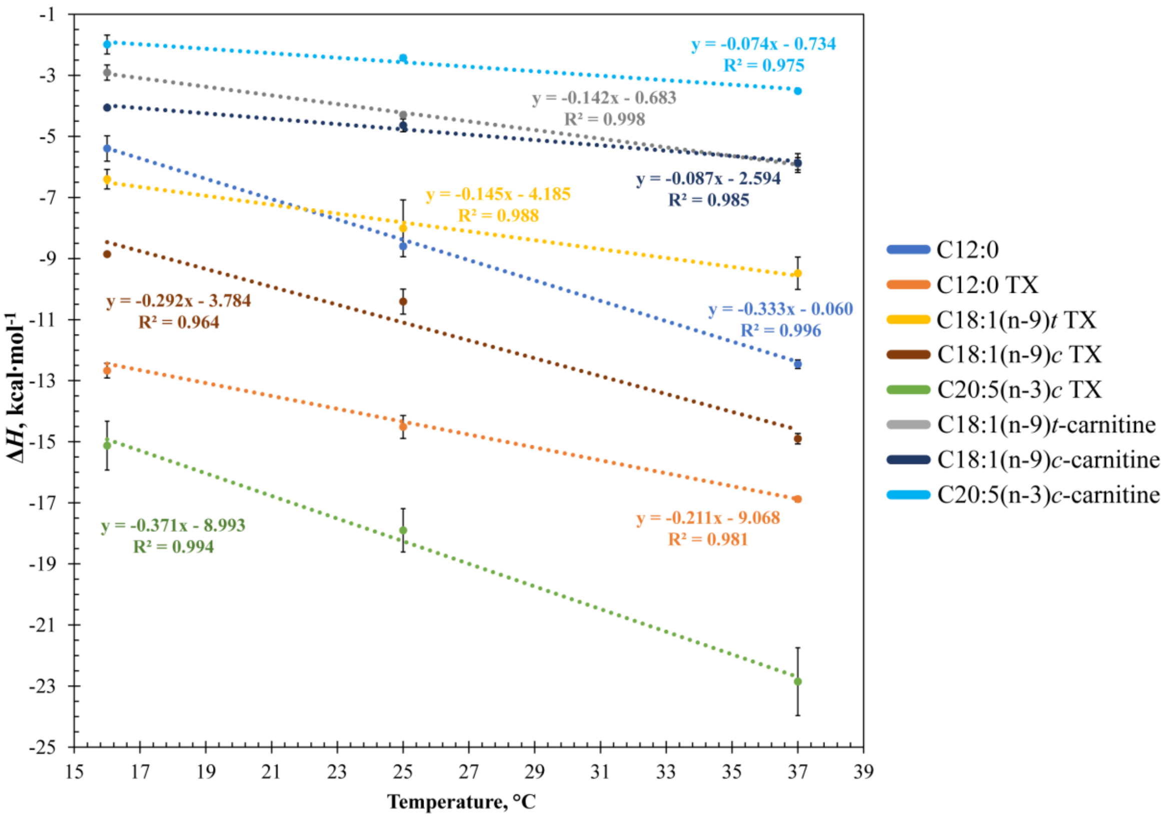

Figure S43. Enthalpy, Δ*H*, values as a function of temperature determined by ITC in 20 mM potassium phosphate buffer pH 7.6 and 50 mM KCl. The slope of each line represents the heat capacity change, ΔC_p, of the FABP3-LCFA or FABP3-LCAC complex.

$$\Delta \boldsymbol{H_T} = \Delta \boldsymbol{H_{T_0}} + \Delta \boldsymbol{C_p}(\boldsymbol{T} - \boldsymbol{T_0}) \quad \text{(S1)}$$

where ΔH_T –enthalpy of ligand binding at temperature T, kcal·mol^{-1};

ΔH_{T_0} – reference enthalpy of ligand binding at temperature T_0, kcal·mol^{-1};

ΔC_p – change in heat capacity, kcal·mol^{-1}·K^{-1};

T – temperature, K;

T_0 – reference temperature, K.

$$\Delta \boldsymbol{S_T} = \Delta \boldsymbol{S_{T_0}} + \Delta \boldsymbol{C_p}\left(\boldsymbol{ln}\frac{\boldsymbol{T}}{\boldsymbol{T_0}}\right) \quad \text{(S2)}$$

where ΔS_T – entropy of ligand binding at temperature T, kcal·mol^{-1};

ΔS_{T_0} – reference entropy of ligand binding at temperature T_0, kcal·mol^{-1};

ΔC_p – change in heat capacity, kcal·mol^{-1}·K^{-1};

T – temperature, K;

T_0 – reference temperature, K.

$$\Delta G_T = \Delta H_T - T\Delta S_T \quad \text{(S3)}$$

where ΔG_T – Gibbs free energy of ligand binding at temperature T, kcal·mol^{-1};

ΔH_T – enthalpy of ligand binding at temperature T, kcal·mol^{-1};

ΔS_T – entropy of ligand binding at temperature T, kcal·mol^{-1};

T – temperature, K.

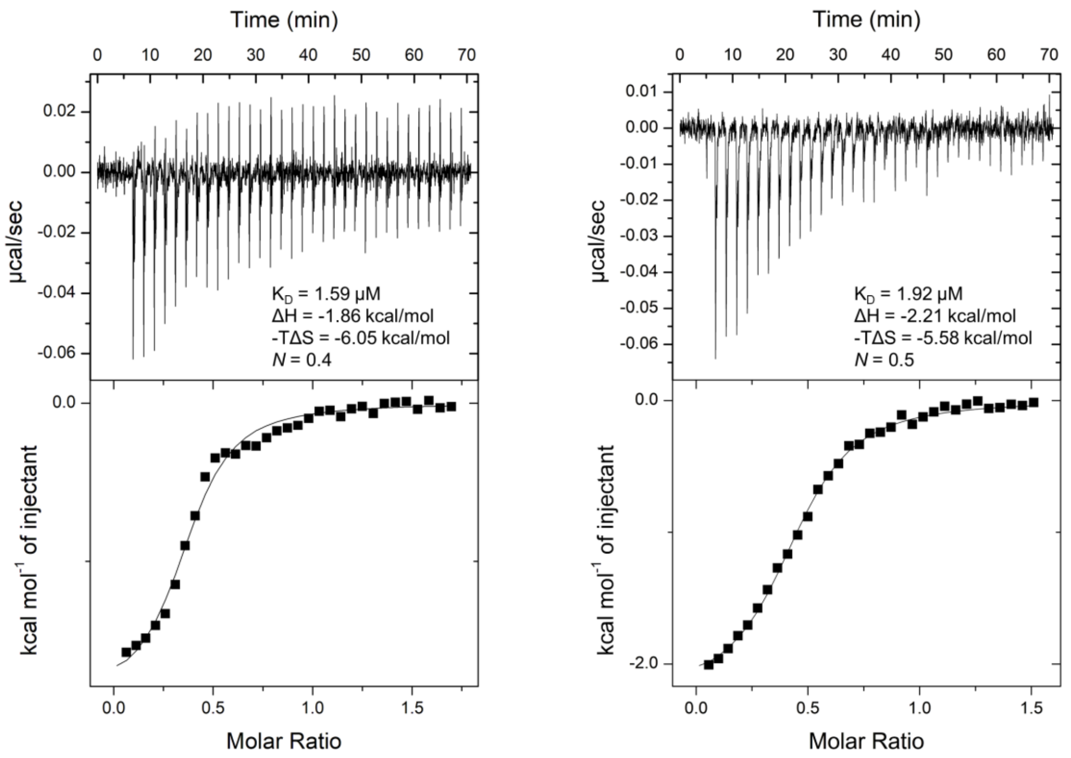

Figure S44. ITC titration data for FABP3 saturated with C8:0 competitive binding with C18:1(n-9)*t*-carnitine at 25 °C in 20 mM potassium phosphate buffer pH 7.6 and 50 mM KCl.

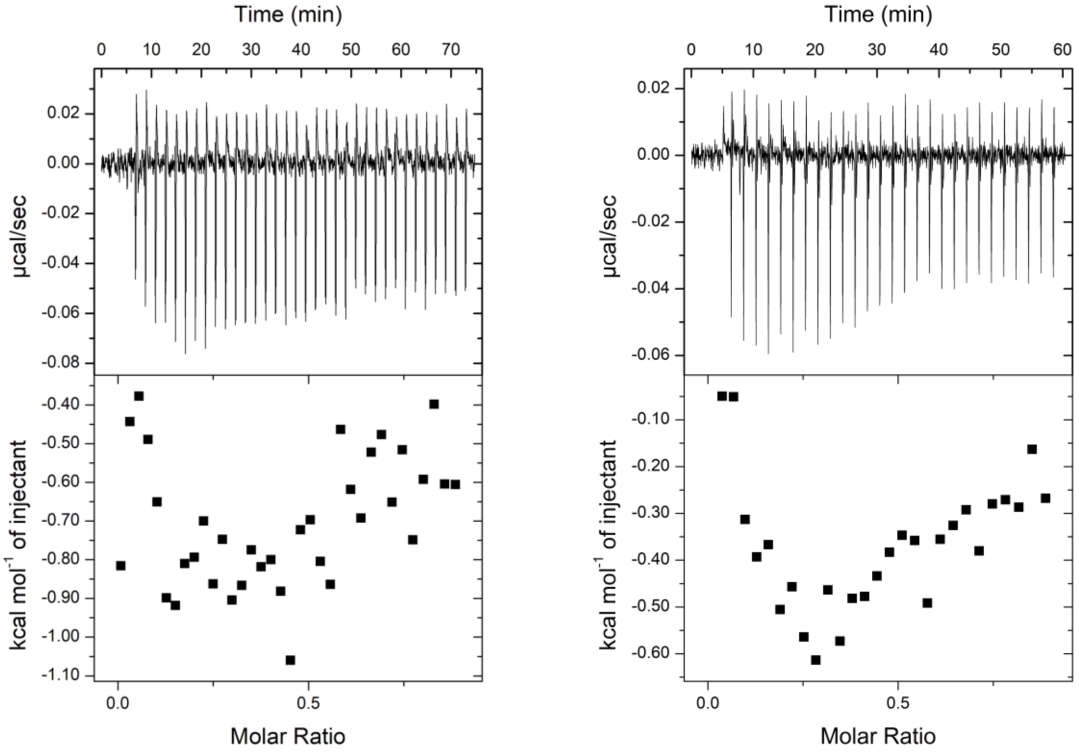

Figure S45. ITC titration data for FABP3 saturated with C10:0 competitive binding with C18:1(n-9)*t*-carnitine at 25 °C in 20 mM potassium phosphate buffer pH 7.6 and 50 mM KCl.

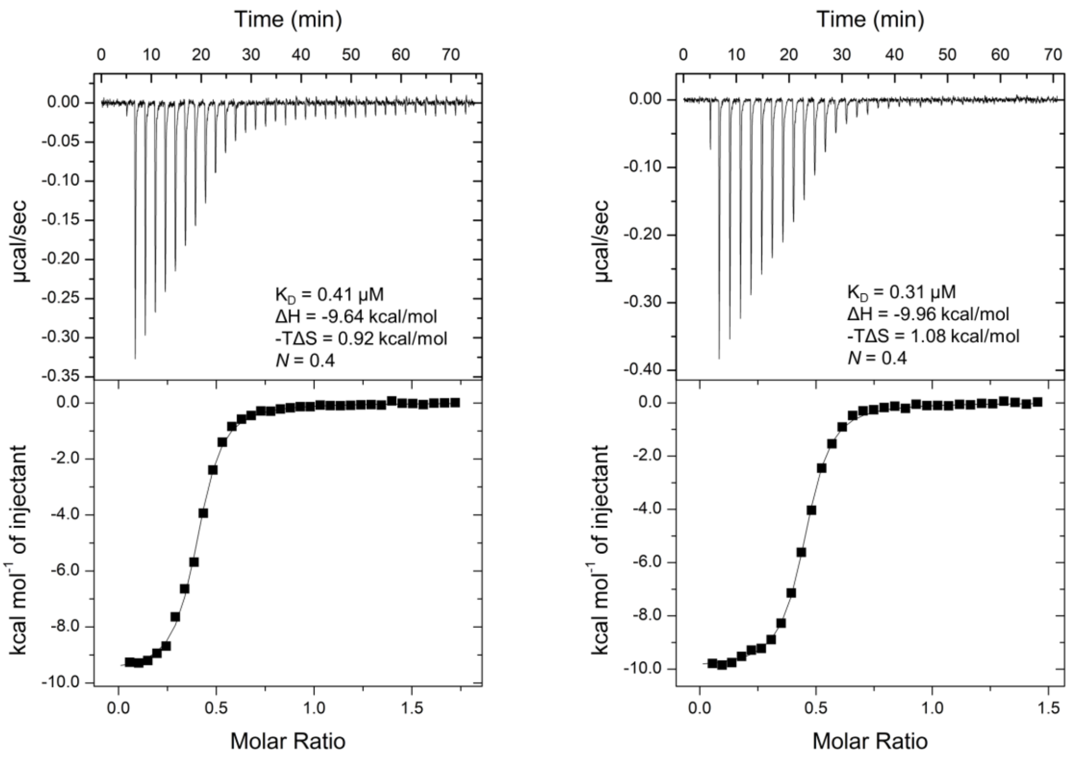

Figure S46. ITC titration data for FABP3 saturated with C18:1(n-9)*t*-carnitine competitive binding with C10:0 at 25 °C in 20 mM potassium phosphate buffer pH 7.6 and 50 mM KCl.

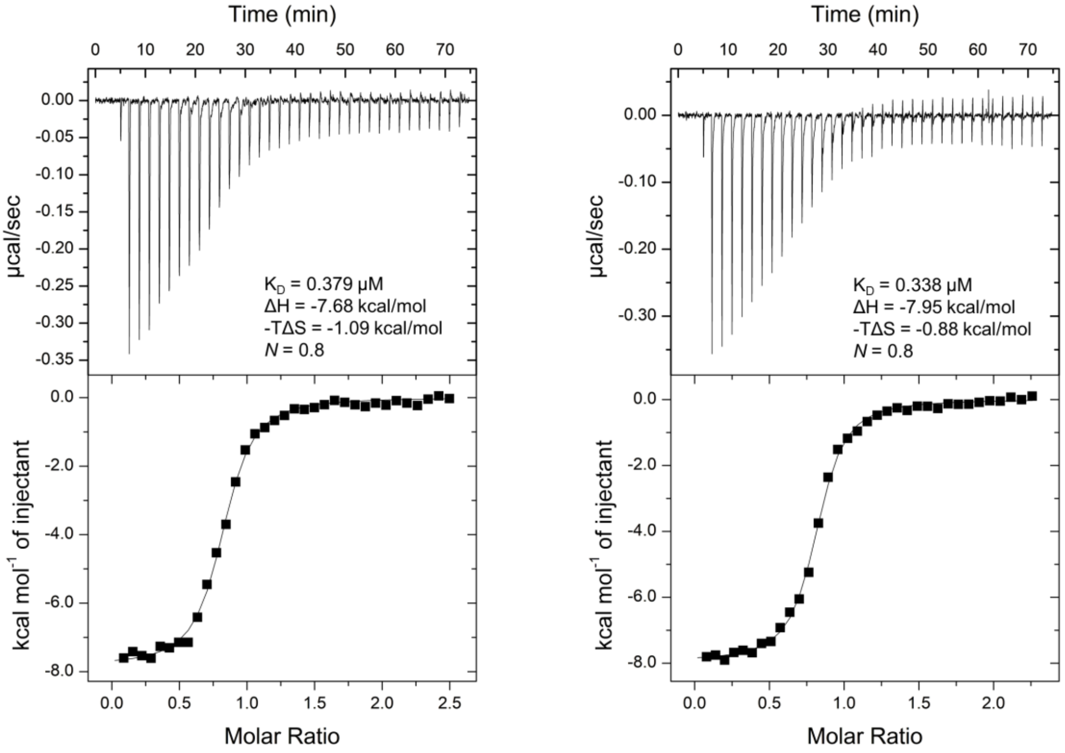

Figure S47. ITC titration data for FABP3 saturated with C18:1(n-9)*t*-carnitine competitive binding with C12:0 at 25 °C in 20 mM potassium phosphate buffer pH 7.6 and 50 mM KCl.

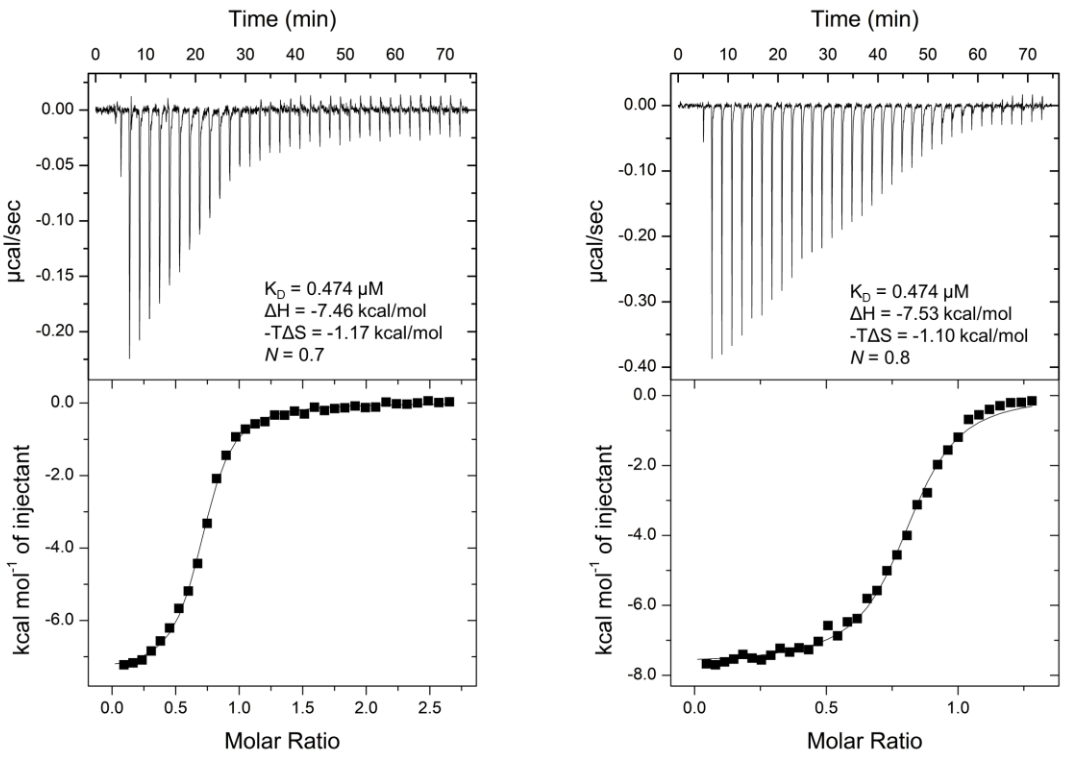

Figure S48. ITC titration data for FABP3 saturated with C18:1(n-9)*t*-carnitine competitive binding with C16:0 at 25 °C in 20 mM potassium phosphate buffer pH 7.6 and 50 mM KCl supplemented with 0.10% (v/v) Triton™ X-100.

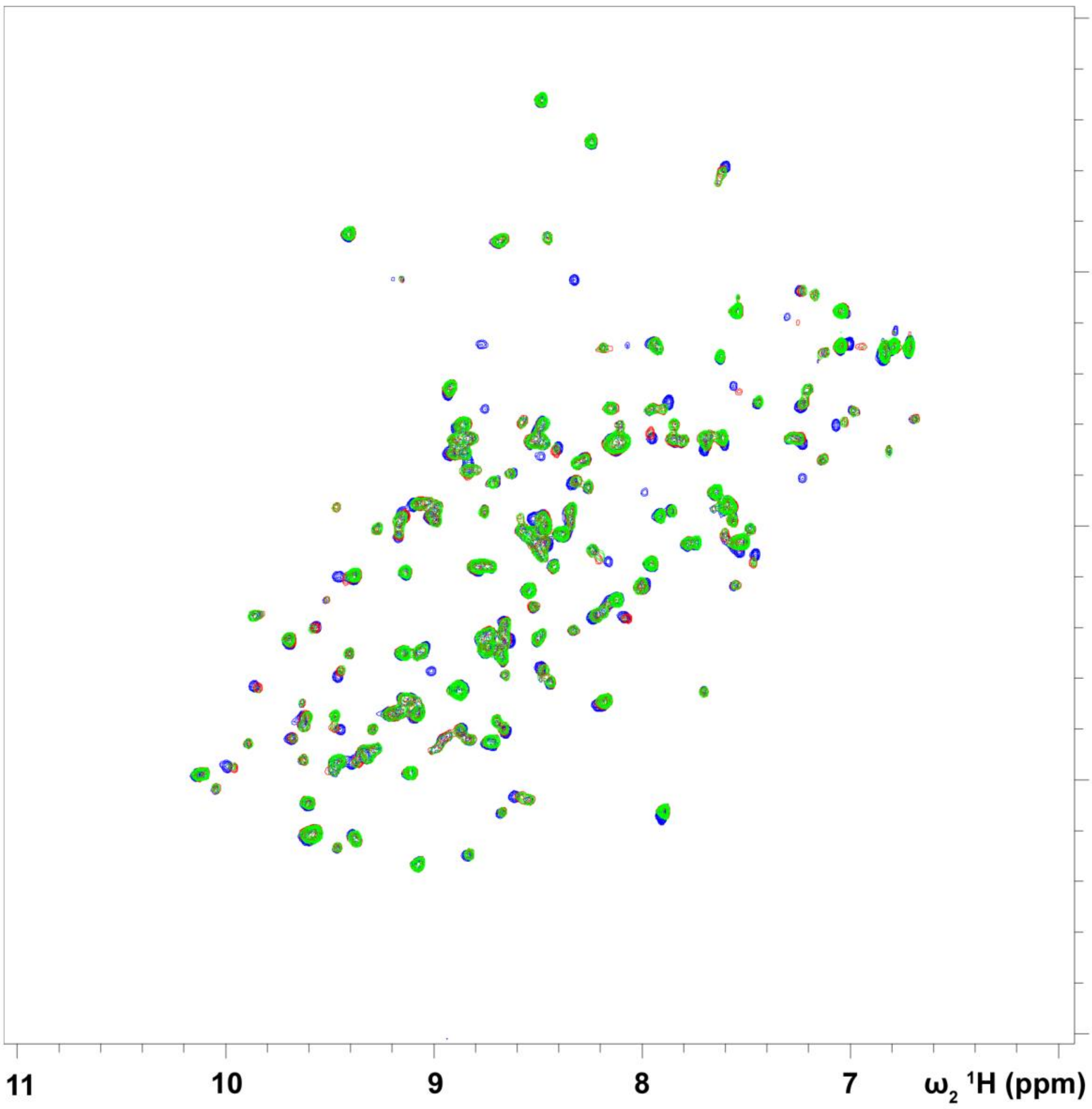

Figure S49. 2D ^{1}H-^{15}N-HSQC spectra of human FABP3 in 20 mM potassium phosphate buffer pH 7.6 and 50 mM KCl. Apo-FABP3 shown in blue, FABP3-C18:1(n-9)*t*-carnitine complex in buffer without additives – in red and with 0.1% (v/v) Triton™ X-100 – in green.

Table S1. ITC Results of the FABP3 Interaction with FA and AC in Competitive Binding Experiments in 20 mM Potassium Phosphate Buffer pH 7.6 and 50 mM KCl at 25 °C.

Compound	C18:1(n-9)*t*-carnitine	C8:0 vs C18:1(n-9)*t*-carnitine		C10:0	C18:1(n-9)*t*-carnitine vs C10:0		C12:0	C18:1(n-9)*t*-carnitine vs C12:0		C16:0 +TX	C18:1(n-9)*t*-carnitine vs C16:0 +TX	
Fitting Model	Reference, One Set of Sites	One Set of Sites	Competitive Binding	Reference	One Set of Sites	Competitive Binding	Reference	One Set of Sites	Competitive Binding	Reference	One Set of Sites	Competitive Binding
K_D, μM	2.19 ± 0.04	1.8 ± 0.2	0.002 ± 0.000	0.252 ± 0.004	0.36 ± 0.07	0.002 ± 0.000	0.20 ± 0.02	0.36 ± 0.03	0.001 ± 0.001	0.14 ± 0.02	0.47 ± 0.00	0.003 ± 0.000
ΔG, $kcal \cdot mol^{-1}$	-7.72 ± 0.02	-7.85 ± 0.08	-11.7 ± 0.1	-9.01 ± 0.01	-8.8 ± 0.1	-11.8 ± 0.1	-9.12 ± 0.06	-8.80 ± 0.04	-12.5 ± 0.9	-9.34 ± 0.07	-8.63 ± 0.01	-11.62 ± 0.05
ΔH, $kcal \cdot mol^{-1}$	-4.29 ± 0.06	-2.0 ± 0.2	-12.0 ± 0.3	-11.09 ± 0.08	-9.8 ± 0.2	-14.1 ± 0.2	-8.60 ± 0.08	-7.8 ± 0.2	-12.1 ± 0.2	-8.7 ± 0.9	-7.49 ± 0.05	-11.77 ± 0.07
$-T\Delta S$, $kcal \cdot mol^{-1}$	-3.43 ± 0.08	-5.8 ± 0.3	0.32 ± 0.09	2.08 ± 0.08	1.0 ± 0.1	0.43 ± 0.03	-0.53 ± 0.02	-1.0 ± 0.1	-0.4 ± 1.1	-0.6 ± 0.8	-1.14 ± 0.05	0.2 ± 0.1
N	0.5	0.5	0.5	1.0	0.5	0.5	1.0	1.0	1.0	1.0	1.0	1.0

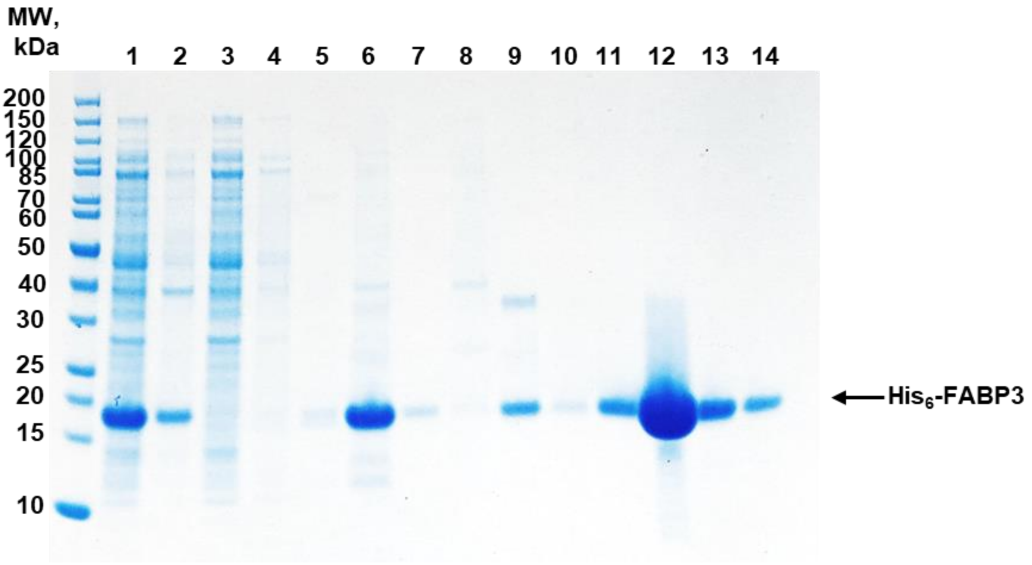

Figure S50. SDS-PAGE analysis of FABP3 purification stages. Lane 1 – supernatant; lane 2 – cell pellets; lanes 3-5 – HisTrap™ flow through; lanes 6-7 – FABP3 eluted from HisTrap™; lanes 8-10 – FABP3 aggregates and lanes 11-14 – FABP3 monomer eluted from Superdex™ 75*pg* 16/600.

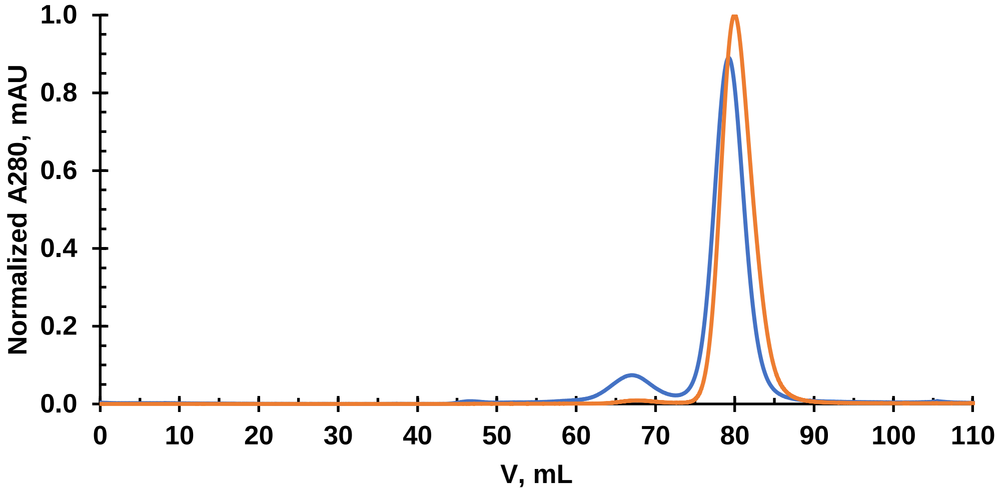

Figure S51. Superposition of size-exclusion chromatograms performed on HiLoad Superdex™ 75*pg* 16/600 for His-tagged (blue) and cleaved (orange) FABP3 normalized by absorption values at 280 nm.

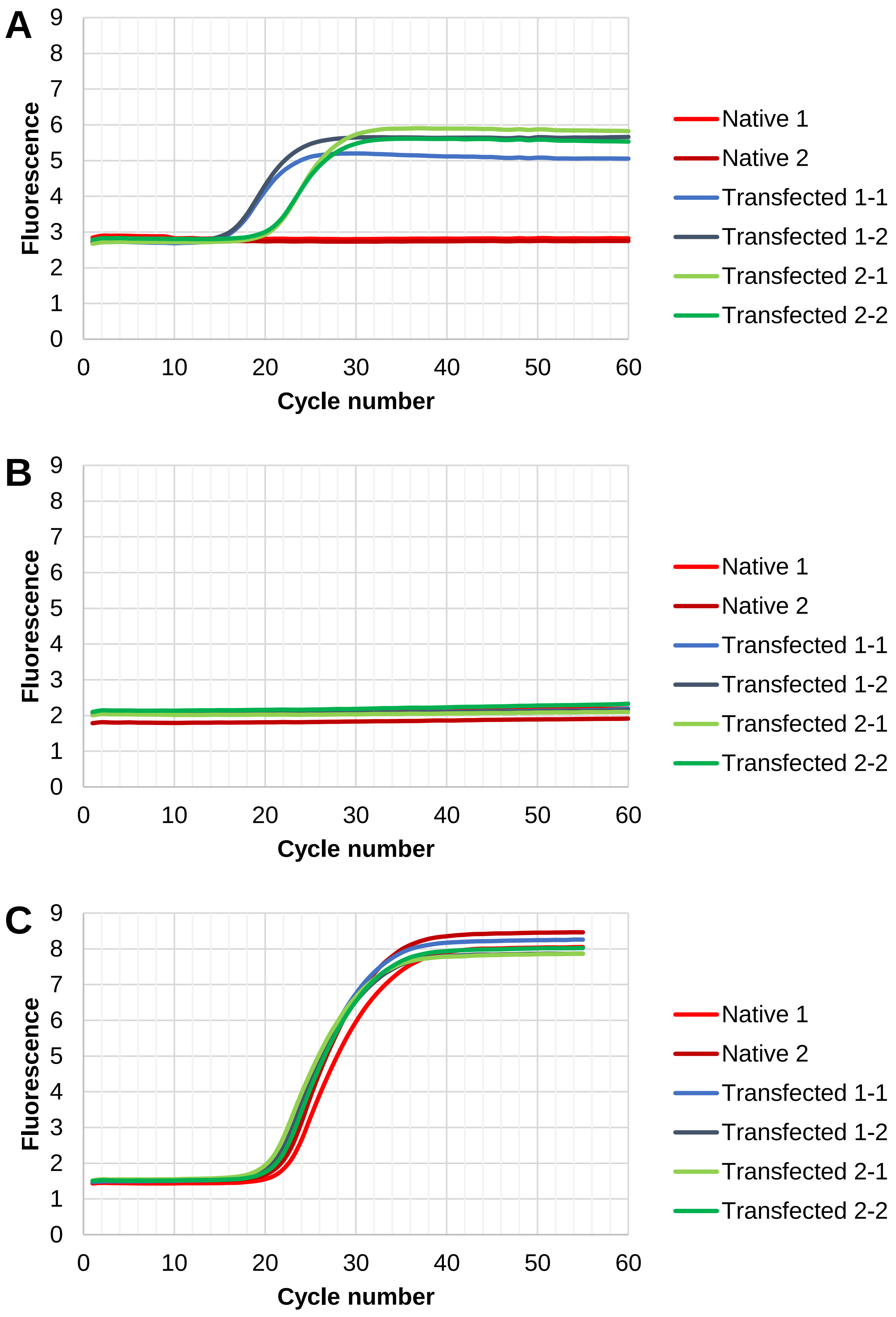

Figure S52. Quantitative PCR analysis of (**A**) mouse-FABP3, (**B**) human-FABP3, and (**C**) human glucose-6-phosphate isomerase (GPI, housekeeping) in native and two sets of transfected PANC-1.

Table S2. Primer sequences and gene accession numbers used in quantitative PCR analysis

Target mRNA	Forward (F) and reverse (R) primer sequences	NCBI accession number
Human FABP3	F: 5'-GCACCTGGAAGCTAGTGGAC-3'	NM_001320996.1
	R: 5'-AGTGTCACAATGGACTTGACC-3'	
Mouse FABP3	F: 5'-TAGGGAGCTAGTTGACGGGA-3'	NM_010174.2
	R: 5'-CGTCGCCTCCTTCTCATAAGT-3'	
Human GPI	F: 5'- GAGCCTTGGTCGCCATGTAT-3'	NM_001184722
	R: 5'- CAGAGGAGGCTGCAGATGAG-3'	

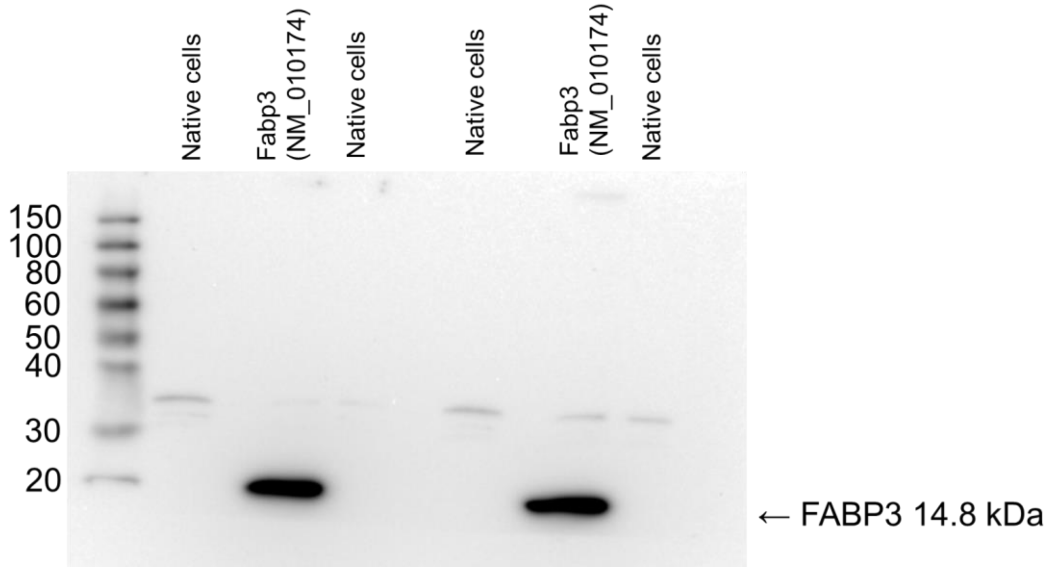

Figure S53. Western Blot image of PANC-1 cells transfected with FABP3 (NM_010174) Mouse Tagged ORF Clone plasmid cat# MR200776 (OriGene Technologies, Inc., Rockville, Maryland, USA) following manufacturer's recommended protocol. Primary antibody: Clone OTI4C5, Anti-DDK (FLAG) monoclonal antibody (Origene, CAT#: TA50011-100) (1:1000). Secondary antibody: Anti-mouse IgG, HRP-linked Antibody CST signaling (#7076) (1:50 000).
